# Fitting parameters and therapies of ODE tumor models with senescence and immune system

**DOI:** 10.1007/s00285-023-02000-9

**Published:** 2023-10-08

**Authors:** F. Guillén-González, E. Sevillano-Castellano, A. Suárez

**Affiliations:** Dpto. Ecuaciones Diferenciales y Análisis Numérico and IMUS, Facultad de Matemáticas, Universidad de Sevilla, C/ Tarfia, S/N, 41012 Sevilla, Spain

**Keywords:** Quasispecies ODE systems, Tumor invasion, Identifiability PCA method, Identifiability Eigenvalues method, Combination of therapies, 34A34, 92B05, 92C50

## Abstract

This work is devoted to introduce and study two quasispecies nonlinear ODE systems that model the behavior of tumor cell populations organized in different states. In the first model, replicative, senescent, extended lifespan, immortal and tumor cells are considered, while the second also includes immune cells. We fit the parameters regulating the transmission between states in order to approximate the outcomes of the models to a real progressive tumor invasion. After that, we study the identifiability of the fitted parameters, by using two sensitivity analysis methods. Then, we show that an adequate reduced fitting process (only accounting to the most identifiable parameters) gives similar results but saving computational cost. Three different therapies are introduced in the models to shrink (progressively in time) the tumor, while the replicative and senescent cells invade. Each therapy is identified to a dimensionless parameter. Then, we make a fitting process of the therapies’ parameters, in various scenarios depending on the initial tumor according to the time when the therapies started. We conclude that, although the optimal combination of therapies depends on the size of initial tumor, the most efficient therapy is the reinforcement of the immune system. Finally, an identifiability analysis allows us to detect a limitation in the therapy outcomes. In fact, perturbing the optimal combination of therapies under an appropriate therapeutic vector produces virtually the same results.

**Supplementary Information:**

The online version contains supplementary material available at 10.1007/s00285-023-02000-9.

## Introduction

The mathematical modelling of biological processes is a challenging topic. In fact, once a model is able to reproduce (in part) a biological process, then it will be possible to check the behaviour of this model in many different situations. Of course, these great variability of situations are not possible to get using in vitro or in vivo experiments. For example, the fitting of some model parameters from experimental data or expected biological behaviour is a very important task, especially when the specific values of these parameters are not known beforehand. In particular, a sensitivity analysis allows us to simplify the fitting problem by eliminating in this process the least identifiable parameters with respect to the system response. On the other hand, for the case of disease models, the computation of optimal therapy combinations can be offered as an adjunct to assist clinical staff decision making. In particular, the mathematical study of the different effects of the therapy combinations can lead to detect the least identifiable therapeutic directions. Then, perturbations of the optimal combination of therapies in these least identifiable directions can produce similar (optimal) effects. This type of information could again help to assist clinical staff decision making, because they can change adequately the combination of therapies producing in practice the same optimal results.

Our main goal in this paper is to study the previous issues in a particular ODE system modeling a tumor invasion in a tissue. For this, our starting point is the ODE quasispecies systems studied in Poyatos and Carnero (2004). In this case, a given tissue is considered in which different cell types, in terms of replicative potential, arise during a tumor proliferation. The most complete model in Poyatos and Carnero (2004) describes a cells population presenting four different states $y_i=y_i(t), i=1,2,3,4,$ interacting in a limited environment: replicative cells $y_1$ (*“normal somatic cells with limited replicative potential”*), senescent cells $y_2$ (*“characterized by terminal growth arrest”*), extended lifespan cells $y_3$ (*“which arises by an extension of lifespan bypassing the senescent limit”*) and immortal cells $y_4$ (*“the final replicative stage experienced by cells before becoming tumor cells”*). Specifically, the system discussed in Poyatos and Carnero (2004) is

1 $$\begin{aligned} \left\{ \begin{array}{lr} y'_1 = (r_1 Q_1 - E ( \underline{y} ) )y_1 \\ y'_2 = - E ( \underline{y} ) y_2 + w_{21}y_1 + w_{23}y_3 \\ y'_3 = (r_3 Q_3 - \delta _3 - E ( \underline{y} ) )y_3 + w_{31}y_1 \\ y'_4 = (r_4 - \delta _4 - E ( \underline{y} ) )y_4 + w_{43}y_3 \end{array} \right. \end{aligned}$$

where

$$\begin{aligned} E ( \underline{y} ) = r_1y_1+(r_3 - \delta _3 ) y_3 + (r_4 - \delta _4 ) y_4. \end{aligned}$$

Here, coefficients $r_i$ and $\delta _i$ are the average growth and death rate of each population $y_i$, $Q_i\in [0,1]$ specifies the probability of $y_i$ cells preserve its proliferative potential, replicative cells could become senescent with rate $w_{21}$ or enter in a state of temporal extension of their lifespan with rate $w_{31}$; $w_{43}$ and $w_{23}$ denote the transition rate of $y_3$ cells to become immortal and senescent, respectively. In fact, the probability of $y_1$ and $y_3$ cells for not preserve its state can be rewritten respectively as

$$\begin{aligned} r_1(1-Q_1)=w_{21}+w_{31}\quad \hbox {and} \quad r_3(1-Q_3)=w_{23}+w_{43}. \end{aligned}$$

Moreover, in order to reduce the numbers of parameters in (1), we introduce two dimensionless positive numbers $\alpha ,\beta \in (0,1)$ such that

$$\begin{aligned} w_{21}= & {} \alpha r_1(1-Q_1),\;w_{31}=(1-\alpha )r_1(1-Q_1),\quad \\ w_{23}= & {} \beta (1-Q_3),\; w_{43}=(1-\beta )r_3(1-Q_3). \end{aligned}$$

**Fig. 1:**
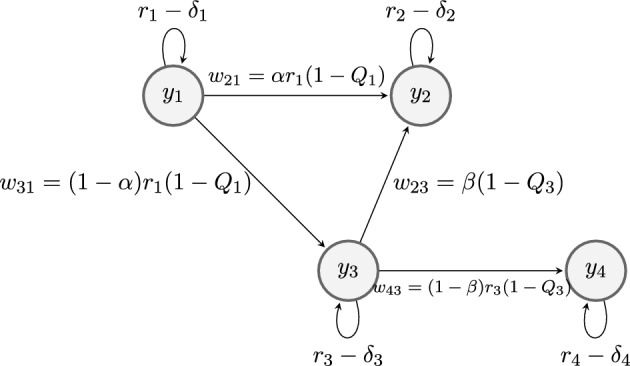
Flow diagram of (1) given in Poyatos and Carnero (2004). System (1) is a compartmental model where proliferative cells ($y_1$) can pass to either senescent ($y_2$) or extended lifespan cells ($y_3$) and extended lifespan cells pass to either senescent or immortal cells ($y_4$)

Then, $\alpha$ (resp. $\beta$) is the ratio of replicative (resp. extended lifespan) cells transforming into senescent ones, with respect to all replicative (resp. extended lifespan) cells that change of state. In Fig. 1 we describe the flow diagram of (1).

To arrive system (1), three models are studied in Poyatos and Carnero (2004): the first one only with replicative and senescent cells, the second one adding the extended lifespand cells and the third one adding the immortal cells:

- The first model, with $(y_1,y_2)$ variables, *“describes tissues without any mutation altering the normal replicative behavior of cells”*. Replicative cells could become senescent, a transition mainly associated to telomere shortening. It is shown in Poyatos and Carnero (2004) that the presence of senescent cells affects to the competition for the space, letting a coexistence equilibrium $(y_1^*,y_2 ^*)$ between both type of cells.
- The second model, with $(y_1,y_2,y_3)$ variables, shows *“different equilibrium populations of extended lifespan cells”*. Indeed, for low or high transmission parameter $1-Q_1$ of the replicative cells, replicative or extended lifespan cells invade, respectively.
- The third model (1), with $(y_1,y_2,y_3, y_4)$ variables, where the main conclusions depend again on $Q_1$. Indeed, for low $Q_1$, immortal cells invade whereas for high $Q_1$ *“immortal cells only invade in a parameter regime of low instability”*. Otherwise, we could have either invasion of extended lifespan cells or *“coexistence of all types of cells in equilibrium”*.

Other examples on biological quasispecies ODE systems can be found in the literature. In Yegorov et al. (2020), a quasispecies model is studied for a microbial population, including effects of growth and degeneration of the total microbes population (calling them *“open quasispecies models”*). On the other hand, a quasispecies ODE system for a finite age-dependent classes of cells interacting with a density of nutrients is analyzed in De Leenheer et al. (2010). In this model, the division rate of cells in senescence classes increases with respect to a growth-limiting nutrient and decreases with respect to an antibiotic treatment. Then, a global stability analysis provides sufficient conditions implying stabilization around a coexistence steady state.

The main objectives (and some of the conclusions) of this paper are the following:

1. Introduce two new models extending the four variables $(y_1,y_2,y_3, y_4)$ model (1) given in Poyatos and Carnero (2004), adding first a fifth state $y_5$ for the tumor cells, see (7) below, and considering after the interaction of these five states with other type of cells, the immune cells ($y_6$), see (8) below. On the other hand, we introduce the application of two therapies in the first model to shrink the tumor, while the replicative and senescent cells dominate: either eliminate directly tumor cells or pass tumor cells to senescent ones. Moreover, the introduction of immune cells in the second model allows us to introduce a third therapy by enforcing the immune system.
2. Fit the parameters that quantify transmission between different states in these two new models, in order to approach (a progressive in time) tumor invasion. In general, this fitting process is very interesting from the biological point of view, because in many real situations the specific values of some parameters are unknown. In addition, we apply two identifiability methods based on the sensibility analysis, namely the Principal Component Analysis (*PCA*) and the *Eigenvalues* method. These methods will allow to detect which of the optimal parameters are no identifiable and therefore the fitting process can be simplified fixing the values of these no identifiable parameters. For instance, in this paper we infer that the five transmission parameters could be reduced to the three most identifiable ones.
3. Fit the optimal combination of therapy parameters to eradicate (progressively in time) the tumor, starting at different states of tumor invasion: early, intermediates and advanced states. Then, we verify that the optimal therapy treatment varies depending on the different states of tumor. For instance, we infer that the therapy strengthening immune cells is always the most effective, and secondly the therapy passing tumor cells to senescent ones is desirable for early states of the tumor, while the therapy eradicating directly tumor cells is desirable for more advanced tumors.
4. Detect the practical dimensionality of the optimal treatment of the therapies, i.e. the number of different outcomes produced by small perturbations of all therapy parameters at the same time. For example, using the eigenvalue identifiability method, we deduce that, although the three therapies do not have negligible effects when varied independently, these therapies could be appropriately modified (in the direction of eigenvectors with very small eigenvalues) while their optimal effects are preserved.

The rest of the paper is organized as follows. In Sect. 2, the two new quasispecies models are described in detail, fitting the transmission parameters via a least square cost functional in Sect. 3. In Sect. 4, the two identifiability methods, PCA and Eigenvalues, are described and applied to reduce the number of the fitted transmission parameters from five to three. We introduce three different therapies in Sect. 5, obtaining optimal combination of therapies and studying the identifiability of each therapy acting separately in Sect. 6. In Sect. 7 we observe a reduction in the dimensionality of the therapies’ results when these are applied jointly. Finally, some conclusions from the biological point of view are given in Sect. 8.

This work has a supplementary material where interested readers can see all computational results.

## Modeling by quasispecies *ODE* systems

### 5-populations model

We consider the time dynamic of a cells population, which adopt five different states, by means of the vectorial variable $\underline{y} \left( t \right) := \left( y_1 \left( t \right) , \dots , y_5 \left( t \right) \right) ^t \in {\mathbb {R}}^5$ in the time interval $t \in [0, T]$. They correspond to replicative, senescent, extended lifespan, immortal and tumor cells, respectively. In fact, each $y_i \left( t \right)$ variable represents the volume fraction of the $i^{th}$ cell population (with respect to the total population) at time *t*, hence they satisfy the following properties, characterizing the so-called (closed) quasispecies models:

2 $$\begin{aligned} \left\{ \begin{aligned} 0 \le y_i \left( t \right) \le 1,&\quad \forall i, \,\,\forall t \in [0, T],\\ \displaystyle \sum ^5_{i=1} y_i(t) = 1,&\quad \forall t \in [0, T].\\ \end{aligned} \right. \end{aligned}$$

We consider the following quasispecies ODEs system:

3 $$\begin{aligned} \left\{ \begin{array}{lr} y'_1 = (r_1 Q_1 -\delta _1 - E (\underline{y} ) )y_1 \\ y'_2 = (r_2 -\delta _2 - E ( \underline{y} ) )y_2 + \alpha r_1(1 - Q_1)y_1 + \beta r_3(1 - Q_3)y_3 + r_5(y_2)(1 - Q_5)y_5 \\ y'_3 = (r_3 Q_3 - \delta _3 - E (\underline{y} ) )y_3 + (1 - \alpha )r_1(1 - Q_1)y_1 \\ y'_4 = (r_4 Q_4 - \delta _4 - E (\underline{y} ) )y_4 + (1 - \beta )r_3(1 - Q_3)y_3 \\ y'_5 = (r_5(y_2) Q_5 - \delta _5 - E ( \underline{y} ) )y_5 + r_4(1 - Q_4)y_4 \\ \end{array} \right. \end{aligned}$$

jointly to the initial condition at $t = 0$,

$$\begin{aligned} \underline{y} \left( 0 \right) =\underline{y}_0:= \left( y_{0_1}, y_{0_2}, y_{0_3}, y_{0_4}, y_{0_5} \right) ^t,\quad \hbox {with } y_{0_i} \ge 0, \quad \forall \, i=1,\ldots ,5. \end{aligned}$$

**Fig. 2:**
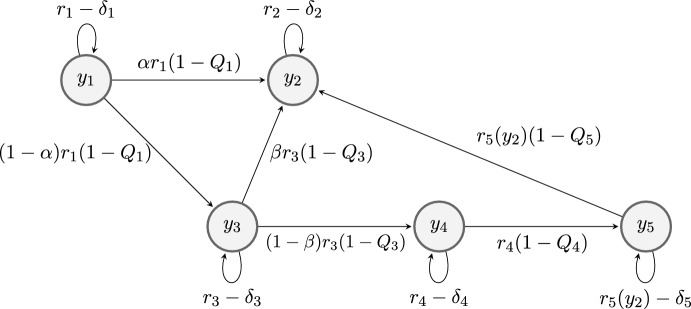
Flow diagram of the 5-populations model. System (3) is a compartmental model where proliferative cells ($y_1$) can pass to either senescent cells ($y_2$) or extended lifespan cells ($y_3$), extended lifespan cells pass to senescent ($y_2$) or immortal cells ($y_4$), and immortal cells become tumor cells ($y_5$). Without therapy tumor cells keep its state ($Q_5=1$), while a therapy where tumor cells pass to senescent cells will be considered ($Q_5<1$)

In Fig. 2 we can see a flow diagram of system (3).

The meaning of the non-negative parameters appearing in the model (3) is:

$$\begin{aligned} \left\{ \begin{array}{l} \begin{array}{rcl} r_i,\delta _i &{}:= &{} \text {birth and death rates of the }i^{th}\text { cell population,} \\ \end{array} \\ \begin{array}{lll} Q_i &{}:= &{} \text {probability of the }i^{th}\text { cells to preserve its state }(Q_i\in (0,1)), \\ \alpha &{}:= &{} \text {ratio of replicative cells transforming into senescent ones} \\ &{} &{} \text {with respect to all replicative cells that change of state }(\alpha \in (0,1)), \\ \beta &{}:= &{} \text {ratio of extended lifespan cells transforming into senescent ones,} \\ &{} &{} \text {with respect to all extended lifespan cells that change of state }(\beta \in (0,1)). \end{array} \end{array} \right. \end{aligned}$$

In fact, $r_i - \delta _i$ is the growth rate of the $i^{th}$ cell population, and $Q_i$ ($i=1,\dots ,5$) and $\alpha ,\beta$ stand for transmission parameters.

In (3), the auxiliary function $E( \underline{y})$ models competition for the space between cells (denoted in Poyatos and Carnero (2004) as *“net rate at which the population dynamics would change”*), which is defined by

4 $$\begin{aligned} E( \underline{y}) = \sum _{i=1}^4 \left( r_i - \delta _i \right) y_i + \left( r_5( y_2 ) - \delta _5 \right) y_5 \equiv \sum _{i=1}^5 \left( r_i - \delta _i \right) y_i \,. \end{aligned}$$

Here, the tumor birth rate $r_5( y_2)$ depends on senescent cells as follows

5 $$\begin{aligned} r_5( y_2) = r_5\left( 1 + \rho \frac{y_2}{y_2 + 1}\right) . \end{aligned}$$

In fact, we are modeling a pro-tumor influence of senescent cells with rate $\rho >0$. This pro-tumor influence can be seen in Vergel et al. (2011), where one can read the sentences *“the senescent cells secrete factors that can disrupt tissue structure and promote cancer progression”* and *“senescent cells might increase the oncogenic potential of tumor cells”*.

Note that (3) is a quasispecies system because satisfies (2). Indeed, it is easy to deduce that each $y_i(t)\ge 0$ for all $i=1,\ldots ,5$ whenever $y_{0_i}\ge 0$ for all $i=1,\ldots ,5$. Moreover, adding all equations in (3) we get:

$$\begin{aligned} \displaystyle \frac{d}{d t} \left( \sum ^5_{i=1} y_i \right) = E ( \underline{y} ) \left( 1 - \sum ^5_{i=1} y_i \right) . \end{aligned}$$

Therefore we deduce that if initially $\sum ^5_{i=1} y_i(0) = \sum ^5_{i=1} y_{0_i} = 1$, then $\sum ^5_{i=1} y_i(t) = 1$ $\forall t \in [0, T]$, hence (2) holds.

In this paper we are going to consider the following biological restrictions:

6 $$\begin{aligned} \left\{ \begin{array}{l} \left. \begin{array}{llll} r_i Q_i - \delta _i &{} > &{} 0 &{} \forall i \in \{ 1, 4, 5 \} \\ r_3 Q_3 - \delta _3 &{} < &{} 0 &{} \\ \end{array} \right\} \begin{array}{l} \text {(the growth rates of all} \\ \text {populations are positive, }\\ \text {except for the extended lifespan cells),} \\ \end{array} \\ \begin{array}{llll} r_2, \delta _2 &{} = &{} 0 &{} \text { (no birth or death of senescent cells),} \\ \delta _1 &{} \approx &{} 0 &{} \text { (scarce death of replicative cells),} \\ \alpha &{} \approx &{} 1 &{} \text { (replicative cells become mainly senescent ones),} \\ \beta &{} \approx &{} 1 &{} \text { (extended lifespan cells become mainly senescent ones),} \\ Q_5 &{} = &{} 1 &{} \text { (tumor cells keep its type).} \\ \end{array} \end{array} \right. \end{aligned}$$

As explained in [6], the progressive shortening of chromosomal ends in each subdivision of cells, provokes that the birth rate of senescent cells vanishes, hence we take $r_2=0$.

With these considerations, model (3) is reduced to:

7 $$\begin{aligned} \left\{ \begin{array}{rcl} y'_1 &{} = &{} (r_1 Q_1 - E(\underline{y}))y_1 \\ y'_2 &{} = &{} - E(\underline{y})y_2 + \alpha r_1(1 - Q_1)y_1 + \beta r_3(1 - Q_3)y_3 \\ y'_3 &{} = &{} (r_3 Q_3 - \delta _3 - E(\underline{y}))y_3 + (1 - \alpha )r_1(1 - Q_1)y_1 \\ y'_4 &{} = &{} (r_4 Q_4 - \delta _4 - E(\underline{y}))y_4 + (1 - \beta )r_3(1 - Q_3)y_3 \\ y'_5 &{} = &{} (r_5(y_2) - \delta _5 - E(\underline{y}))y_5 + r_4(1 - Q_4)y_4 \\ \end{array} \right. \end{aligned}$$

where the functions $E( \underline{y} )$ and $r_5 ( y_2 )$ are given in (4) and (5), respectively.

### 6-populations model

We complete system (7) considering the interaction with the population of immune cells $y_6 = y_6(t)$, arriving to the following extended ODEs system:

8 $$\begin{aligned} \left\{ \begin{array}{rcl} y'_1 &{} = &{} (r_1Q_1 - E(t,\underline{y}))y_1 \\ y'_2 &{} = &{} - E(t,\underline{y})y_2 + \alpha r_1(1 - Q_1)y_1 + \beta r_3(1 - Q_3)y_3 \\ y'_3 &{} = &{} (r_3Q_3 - \delta _3 - E(t,\underline{y}))y_3 + (1 - \alpha )r_1(1 - Q_1)y_1 \\ y'_4 &{} = &{} (r_4Q_4 - \delta _4 - E(t,\underline{y}))y_4 + (1 - \beta )r_3(1 - Q_3)y_3 \\ y'_5 &{} = &{} (r_5( t, y_2, y_6 ) - \delta _5 - E(t,\underline{y}))y_5 + r_4(1 - Q_4)y_4 \\ y'_6 &{} = &{} (r_6(y_5) - \delta _6(y_5) - E(t,\underline{y}))y_6 \\ \end{array} \right. \end{aligned}$$

Now, the auxiliary functions are defined by:

9 $$\begin{aligned} E( t, \underline{y})= & {} \sum _{i=1}^4 (r_i - \delta _i)y_i + (r_5( t, y_2, y_6 ) - \delta _5)y_5 + (r_6(y_5) - \delta _6(y_5)) y_6\nonumber \\\equiv & {} \sum _{i=1}^6 (r_i - \delta _i)y_i, \end{aligned}$$

10 $$\begin{aligned} r_5( t, y_2, y_6 )= & {} r_5 \left( 1 + \rho \frac{y_2}{y_2 + 1} - \sigma \frac{T}{t+T} \frac{y_6}{y_6 + 1} \right) , \end{aligned}$$

11 $$\begin{aligned} r_6( y_5 )= & {} r_6 \left. \frac{2 y_5}{y_5 + 1} \right. , \end{aligned}$$

12 $$\begin{aligned} \delta _6( y_5 )= & {} \delta _6 + \left. \tau \frac{2 y_5}{y_5 + 1} \right. . \end{aligned}$$

In (10), the $y_6$ dependence of $r_5$ shows the anti-tumoral factor of immune cells, where the factor $\frac{T}{t+T}$ stands for the degradation effect through time. Also, the $y_5$ dependence of $r_6$ and $\delta _6$ given in (11) and (12) show the pro-immune and the anti-immune factors of the tumor, respectively. Indeed, the expression given in $r_6(y_5)$ represents the growth of the immune cells with respect to the tumor and $\delta _6(y_5)$ the death of immune cells caused by its fight against the tumor. Consequently, the meaning of the new non-negative parameters is:

13 $$\begin{aligned} \left\{ \begin{array}{rcl} \sigma &{}:= &{} \text {anti-tumoral factor by immune cells;} \\ \tau &{}:= &{} \text {anti-immune factor by tumor cells.} \\ \end{array} \right. \end{aligned}$$

We consider the additional restriction: $r_6 - \delta _6 > 0$.

## Fitting transmission parameters

Our aim is to fit the parameters with more uncertainty, which in this case are the transmission parameters $\left\{ Q_1, Q_3, Q_4, \alpha , \beta \right\}$. For this, we start from a healthy scenario (mainly with replicative cells and a few of immune cells) and we arrive at the tumor invasion. In fact, we choose the following initial and final states:

14 $$\begin{aligned} \left\{ \begin{array}{llllllll} \underline{y}_0 &{} = &{} (0.99, &{}0, &{} 0, &{} 0, &{}0, &{}0.01)^t,\\ \underline{y}_{d} &{} = &{} (0.1, &{}0.1, &{}10^{-7}, &{} 10^{-3}, &{}0.7889999, &{}0.01 )^t. \\ \end{array} \right. \end{aligned}$$

For the 5-populations model, the initial state is fully replicative and the immune part given in (14) adds to the tumor at the final state.

We fix the time interval to $[0,T=10]$ and the following parameter values:

$$\begin{aligned} \left\{ \begin{array}{ll} \underline{r} = (10, 0, 10, 10, 15, 10)^t, &{} \underline{\delta } = (0, 0, 1, 0, 0, 0)^t, \\ \rho = 0.5, \quad \sigma = 0.5, &{} \tau = 0.5, \end{array} \right. \end{aligned}$$

in agreement to the constraints given in (6). At these values, the growth factor for senescent cells disappears and so does the death factor for all cell types except extended lifespan cells. Since the dimensionless parameters $\rho ,\sigma ,\tau \in (0,1)$, we choose the mean value 0.5 for all of them.

For both models (7) and (8), we fit the transmission parameters via an optimal control problem with a cost function $J = J \left( \underline{\theta } \right) = J \left( Q_1, Q_3, Q_4, \alpha , \beta \right)$ defined as:

$$\begin{aligned} J \left( \underline{\theta } \right) = \frac{1}{2}\frac{1}{T} \int ^{T}_{0} \frac{t}{T} \left\| \underline{y} \left( t, \underline{\theta } \right) - \underline{y}_d \left( t \right) \right\| _2^2 \,\, dt. \end{aligned}$$

We take the target function $\underline{y}_d \left( t \right)$ as a linear function between the initial state $\underline{y}_0$ and the final state $\underline{y}_d$ given in (14). The weight *t*/*T* enforces the fitting process at times close to *T*. In practice, we approximate the solution of (7) or (8) and $J \left( \underline{\theta } \right)$ by the trapezium rule associated to a partition $\{ t_k \}^{K}_{k=0}$ of [0, *T*]:

15 $$\begin{aligned} J \left( \underline{\theta } \right) \approx \frac{1}{2}\sum ^{K}_{k=0} \sum _{i} q_{k} \left( {y}_i \left( t_{k}, \underline{\theta } \right) -{y}_{d_{i}} \left( t_{k} \right) \right) ^2. \end{aligned}$$

Here, the weights $q_{k}$ are:

16 $$\begin{aligned} q_{k} = \left\{ \begin{array}{ll} \frac{1}{2 T^2} \left( t_{k+1} - t_{k} \right) t_{k} &{} \text {if }k = 0, \\ \frac{1}{2 T^2} \left( t_{k+1} - t_{k-1} \right) t_{k} &{} \text {if }1\le k< K,\\ \frac{1}{2 T^2} \left( t_{k} - t_{k-1} \right) t_{k} &{} \text {if }k = K. \\ \end{array} \right. \end{aligned}$$

The ranges of fitted parameters are:

$$\begin{aligned} \left\{ \begin{array}{lllllll} Q_i &{} \in &{} [ &{} \varepsilon , &{} 1 - \varepsilon &{} ], &{} \forall i \in \{ 1, 3, 4 \}\\ \alpha &{} \in &{} [ &{} 1 - 10^{-3}, &{} 1 &{} ], \\ \beta &{} \in &{} [ &{} 1 - 10^{-2}, &{} 1 &{} ]. \\ \end{array} \right. \end{aligned}$$

with $\varepsilon = 10^{-2}$, in agreement to the restrictions about $\alpha$ and $\beta$ given in (6).

We use *Matlab* software, specifically the function *ode45* to approach the ODE system (7) and (8) and the function *fmincon* to achieve minimum of (15). Since the optimal control problem can have an important complexity (with different local minima and/or saddle points), we consider a grid with 4 different initializations of each parameter (giving $4^{5} = 1024$ parameter combinations). Then, the best combination of parameters computed and the functional $J \left( \underline{\theta } \right)$ at these optimal parameters are given in Table 1.

**Table 1:** Optimal values of the transmission parameters

Model	${Q_1}$	${Q_3}$	${Q_4}$	${\alpha }$	${\beta }$	*J*
5 Pop.	0.83014	0.09081	0.95000	$1 - 8.8 \cdot 10^{-9}$	$1 - 3.2 \cdot 10^{-12}$	$1.74 \cdot 10^{-2}$
6 Pop.	0.82180	0.08754	0.16769	$1 -1.7 \cdot 10^{-12}$	$1 - 7.0 \cdot 10^{-10}$	$1.46 \cdot 10^{-2}$

First row for the model without immune cells and second row including immune cells. The largest variation occurs for the coefficient $Q_4$, modeling the transmission from immortal to tumor cells

**Fig. 3:**
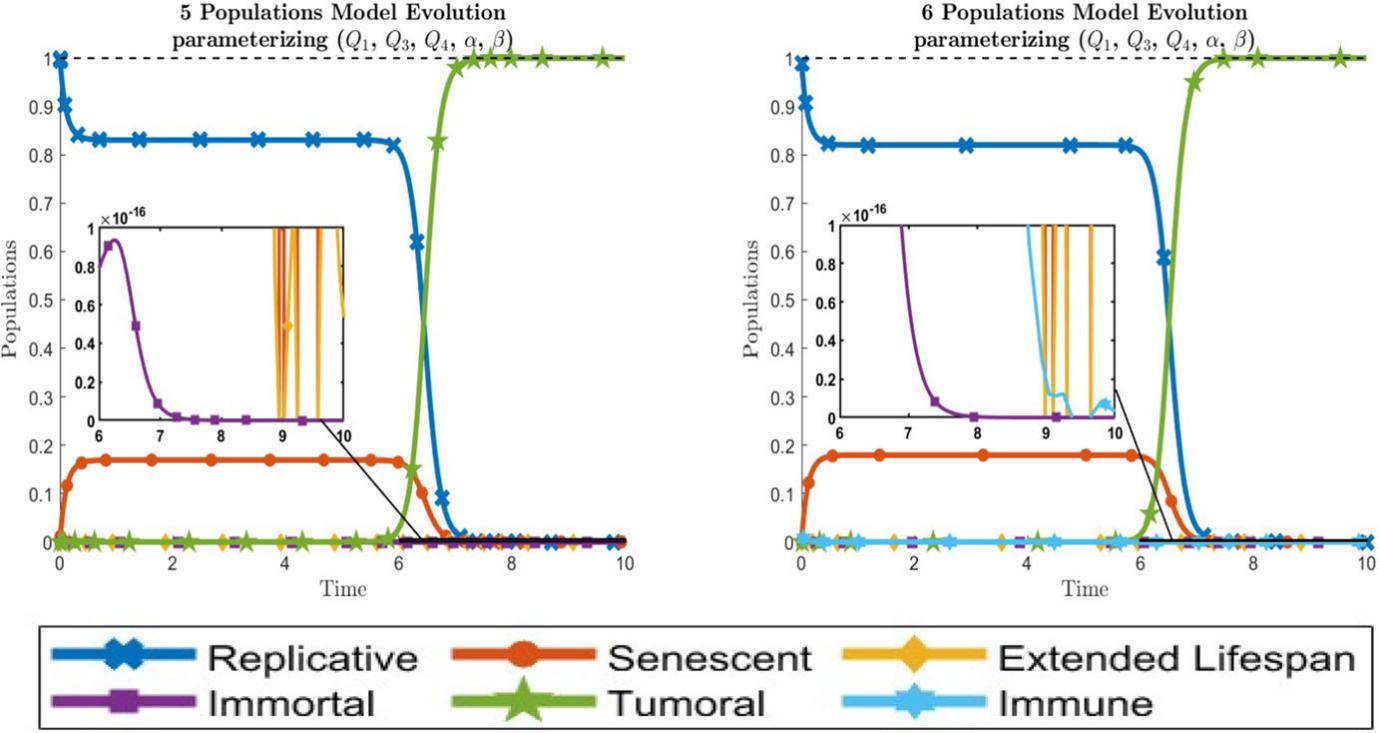
Dynamical behavior of all cell types for the optimal transmission parameters causing the tumor invasion. The left graphic is for the 5-populations model whereas the right graphic is for the 6-populations model. A zoom is also showed to judge the dynamics of low-abundance cell populations such as extended lifespan, immortal or immune cells. Bottom, legend of the six cell types along the paper. Line codes, $y_1$: blue crossed, $y_2$: red circled, $y_3$: yellow diamonded, $y_4$: purple squared, $y_5$: green 5 points stared, $y_6$: cyan 6 points stared (color figure online)

The values of each population at the final time $t=T$ can be found in Supplementary Material A.1. Finally, the time evolution of the populations for each model are represented in Fig. 3.

## Transmission parameters’ identifiability

Once fitted the transmission parameters of the models, we study the identifiability of the optimal parameters, showing which of them are more identifiable and which are more unidentifiable or non-identifiable. We will focus on two methods: the *Principal Component Analysis* (*PCA*) and the *Eigenvalues Method* (see Miao et al. 2011 for more details). Both methods are based on the sensibility analysis, approaching the derivatives of the observed populations with respect to each parameter. In this case, we consider that all populations of the system can be observed. The sensibility derivatives are evaluated either on a set of observation times $\{ t_k \}_{k=1}^ N$ (where experimental data could be given) for the *PCA Method*, or in the partition of [0, *T*] taken in the trapezoidal rule used in (15) to compute the functional cost *J*, for the *Eigenvalue Method*.

Other identifiability methods can be seen in Wieland et al. (2021) and are applied, for instance, in Gabor et al. (2017) and Olufsen and Ottesen (2013). In fact, in Gabor et al. (2017) a methodology is presented for detecting relationships among many parameters, and then an integer optimization is applied to find the largest groups of uncorrelated parameters. In Olufsen and Ottesen (2013) other three different parameter identifiability methods are applied to model heart rate regulation.

### Principal Component Analysis (*PCA*) Method

For this method (explained in pages 21–22 of Miao et al. 2011) we define the following sensitivity coefficients evaluated on a vector of parameters $\underline{\theta }^*$ and normalized by the weights $\sqrt{q_{k}}$:

17 $$\begin{aligned} s^i_{k,j} = \sqrt{q_{k}} \, \frac{\partial y_i \left( t_k, \underline{\theta }^* \right) }{\partial \theta _j} \,\, \forall i,j,k, \end{aligned}$$

where $y_i$ are the observed populations at the model ($i = 1, \ldots , d$); $\theta _j$ the *j*th parameter ($j = 1, \ldots , q$), $t_k$ the *k*th observation time ($k = 1, \dots , N$) and $q_{k}$ the weights at the observation times $t_k$ defined in (16). Then, for each observed population $y_i$, we consider the sensitivity $N \times q$ matrix:

18 $$\begin{aligned} \pmb {S}^i = \left[ \begin{array}{ccc} s^i_{1,1} &{} \cdots &{} s^i_{1,q} \\ \vdots &{} \ddots &{} \vdots \\ s^i_{N,1} &{} \cdots &{} s^i_{N,q} \\ \end{array} \right] \,\, \forall i = 1, \ldots , d. \end{aligned}$$

We will use the *PCA* technique where the (random) variables will be each of the parameters in $\underline{\theta }$ and the entries will be taken at the observation times $\{t_k\}_{k=1}^N$. In order to get the weights (or *“loadings”*) of the original variables (each $j^{th}$ column $s^i_{\cdot ,j}$ of $\pmb {S}^i$) which define the principal components, we will calculate the *eigenvector-eigenvalue* of the covariance matrix of $\pmb {S}^i$. In particular, centering the variables (to get zero mean variables) by

$$\begin{aligned} \pmb {S}^i_c = \left( s^i_{k,j} - \overline{s^i_{\cdot ,j}} \right) _{kj} \end{aligned}$$

where $\overline{s^i_{\cdot ,j}}$ is the mean of the $j^{th}$ column $s^i_{\cdot ,j}$ of $\pmb {S}^i$, then the covariance matrix of $\pmb {S}^i$ is

$$\begin{aligned} Cov \left( \pmb {S}^i \right) = \frac{1}{N-1} \left( \pmb {S}^i_c \right) ^T \pmb {S}^i_c. \end{aligned}$$

Each principal component is associated to each $(\lambda ^i,\underline{\gamma }^i)$ eigenvalue and eigenvector of the matrix $Cov \left( \pmb {S}^i \right)$. We will order the components by their eigenvalues, where the higher $\lambda ^i$ is, the more sensitive data response is with respect to small perturbations through the direction $\underline{\gamma }^i$. In particular, if $\left| \lambda ^i \right|$ is near zero, then the variation of the $y_i$ values is rather flat through the direction $\underline{\gamma }^i$. Let us order the eigenvalues non-decreasingly $0 \le \left| \lambda ^i_1 \right| \le \cdots \le \left| \lambda ^i_q \right|$ and $\underline{\gamma }_1^i, \underline{\gamma }_2^i, \dots , \underline{\gamma }_q^i$ is an orthonormal basis of eigenvectors forming the unitary $q \times q$ matrix:

$$\begin{aligned} \pmb {\Gamma }^i = \left( \underline{\gamma }_1^i, \underline{\gamma }_2^i, \ldots , \underline{\gamma }_q^i \right) = \left[ \begin{array}{ccc} (\gamma _{1}^i)_1 &{} \cdots &{} \gamma _{1,q}^i \\ \vdots &{} \ddots &{} \vdots \\ (\gamma _{1}^i)_q &{} \cdots &{} \gamma _{q,q}^i \\ \end{array} \right] \,\, \forall \, i = 1, \ldots , q. \end{aligned}$$

We will sort the parameter vector $\underline{\theta }=(\theta _1,\dots , \theta _q)$ by their identifiability with respect to each $y ^i$, such that at the end of the process, the ordered parameter remains as $(\theta _{m_1^i},\dots , \theta _{m_q^i})$ where $\theta _{m_1^i}$ and $\theta _{m_q^i}$ will be the least and most identifiable parameters, respectively, with respect to the $y ^i$ values. The sort procedure is as follows:

$$\begin{aligned} m^i_1= & {} \mathop {{{\,\mathrm{{arg\,max}}\,}}}\limits _{1 \le j \le q} \left| (\gamma ^i_{1})_j \right| , \\ m^i_l= & {} \displaystyle \mathop {{{\,\mathrm{{arg\,max}}\,}}}\limits _{1 \le j \le q; \ j \ne m^i_1, \ldots , m^i_{l-1}} \left| (\gamma ^i_{l})_j \right| , \,\,\,\, l =2,\ldots , q. \end{aligned}$$

### Eigenvalues method

This method (explained in page 24 of Miao et al. 2011) uses the elements $$s_{(ki)j} $$, whose definition is the same as $$s^i_{k,j}$$ given in (17), but now evaluated at the partition $$\{t_k\}_{k=1}^{K}$$ of [0, *T*] given in (15). Then, we consider the sensitivity $$d K \times q$$ matrix:

19 $$\begin{aligned} \pmb {S} = \left[ \begin{array}{ccc} s_{(11)1} &{} \cdots &{} s_{(11)q} \\ \vdots &{} \ddots &{} \vdots \\ s_{(1d)1} &{} \cdots &{} s_{(1d)q} \\ \vdots &{} \vdots &{} \vdots \\ s_{(K1)1} &{} \cdots &{} s_{(K1)q} \\ \vdots &{} \ddots &{} \vdots \\ s_{(Kd)1} &{} \cdots &{} s_{(Kd)q} \\ \end{array} \right] . \end{aligned}$$

By considering the linear approximation of $y_i$ along a parameter vector $\underline{\gamma }$,

$$\begin{aligned} y_i(t_k,\underline{\theta }^* + \varepsilon \underline{\gamma }) \approx y_i(t_k,\underline{\theta }^*) +\varepsilon \sum _j \frac{\partial y_i \left( t_k, \underline{\theta }^* \right) }{\partial \theta _j} {\gamma }_j \end{aligned}$$

with $\varepsilon$ any arbitrary small constant, and using that if $\underline{\theta }^*$ is an optimal parameter vector of the cost functional $J(\underline{\theta })$ given in (15), then

$$\begin{aligned} \frac{\partial J}{\partial \theta _j} \left( \underline{\theta }^* \right) =\sum _{k=1} ^K\sum _i \sqrt{q_k} \Big (y_i(t_k, \underline{\theta }^*)-(y_d)_i(t_k) \Big ) s_{(ki)j}(\underline{\theta }^*) = 0,\quad \forall \, j=1,\dots ,q, \end{aligned}$$

we can deduce that any perturbation of the functional cost *J* centered at the minimum $\underline{\theta }^*$ along a vector $\underline{\gamma }$ can be approximated by

20 $$\begin{aligned} J \left( \underline{\theta }^* + \varepsilon \underline{\gamma } \right) - J \left( \underline{\theta }^* \right) \approx \frac{1}{2} \varepsilon ^2 \underline{\gamma }^T \, \pmb {S}^T \pmb {S} \, \underline{\gamma } \end{aligned}$$

where $\pmb {S}$ is the sensitivity matrix given in (19) evaluated at $\underline{\theta }^*$. Therefore, by fixing $(\lambda ,\underline{\gamma })$ an eigenvalue and unit eigenvector of $\pmb {S}^T \pmb {S}$, i.e $\pmb {S}^T \pmb {S} \, \underline{\gamma } = \lambda \underline{\gamma }$ and $\underline{\gamma }^T \underline{\gamma } = 1$, then (20) remains as

$$\begin{aligned} J \left( \underline{\theta }^* + \varepsilon \underline{\gamma } \right) - J \left( \underline{\theta }^* \right) \approx \frac{1}{2} \varepsilon ^2 \lambda \in {\mathcal {O}} \left( \lambda \right) . \end{aligned}$$

Consequently, if $\lambda$ is close to zero, the direction $\underline{\gamma }$ is rather flat with respect to *J*. Then, removing this direction, the optimal problem can be simplified. We will sort the parameters associated to the main component of those directions $\underline{\gamma }$ similarly to the *PCA* technique, by considering $0 \le \left| \lambda _1 \right| \le \cdots \le \left| \lambda _q \right|$ the eigenvalues of $\pmb {S}^T \pmb {S}$ and $( \underline{\gamma }_1, \ldots , \underline{\gamma }_q )$ the corresponding eigenvectors forming an orthonormal basis.

### Transmission parameters’ identifiability

We fix the optimal combination $\underline{\theta }^* = \left( Q_1^*, Q_3^*, Q_4^*, \alpha ^*, \beta ^* \right)$ given in Table 1, and use either the observable times $\{ t_k \}_{k = 1, \dots , N=11} = \{ 0, \dots , 10 \}$ for the *PCA* *Method* or the time partition given in the approximation of the *ode45* program for the *Eigenvalues method*. The eigenvectors and eigenvalues for each method can be seen at Supplementary Material C.1.1 and C.1.2 for 5 and 6-populations models, respectively. Looking at these values, we deduce that the most identifiable parameters of all populations are either first $\beta$ and then $\alpha$ for the 5-populations model or first $\alpha$ and then $\beta$ for the 6-populations model. The identifiability of the other three parameters depends on each population, although for the most numerous populations $y_1$, $y_2$ and $y_5$, the most non-identifiable parameters are $Q_3$ and $Q_4$.

In conclusion, the parameters $Q_3$ and $Q_4$ can be fixed (to simplify the fitting parameter process) whereas the other ones are quite identifiable, with $\beta$ more identifiable than $\alpha$ and $\alpha$ more identifiable than $Q_1$ for the 5-populations model (changing places $\alpha$ and $\beta$ for the 6-populations model).

### Fitting only the identifiable parameters

Once proved that the parameters $Q_3$ and $Q_4$ are unidentifiable near $\underline{\theta }^*$, we fit just the parameters ($Q_1$, $\alpha$, $\beta$), fixing the values of $Q_3$ and $Q_4$ as:

$$\begin{aligned} Q_3 = 0.05,\quad Q_4 = 0.5. \end{aligned}$$

As the cost function is now rewritten as $J = J \left( Q_1, \alpha , \beta \right)$, the number of combinations of the initialization of the parameters is only $4^{3} = 64$ (keeping the 4 different values for each parameter).

**Fig. 4:**
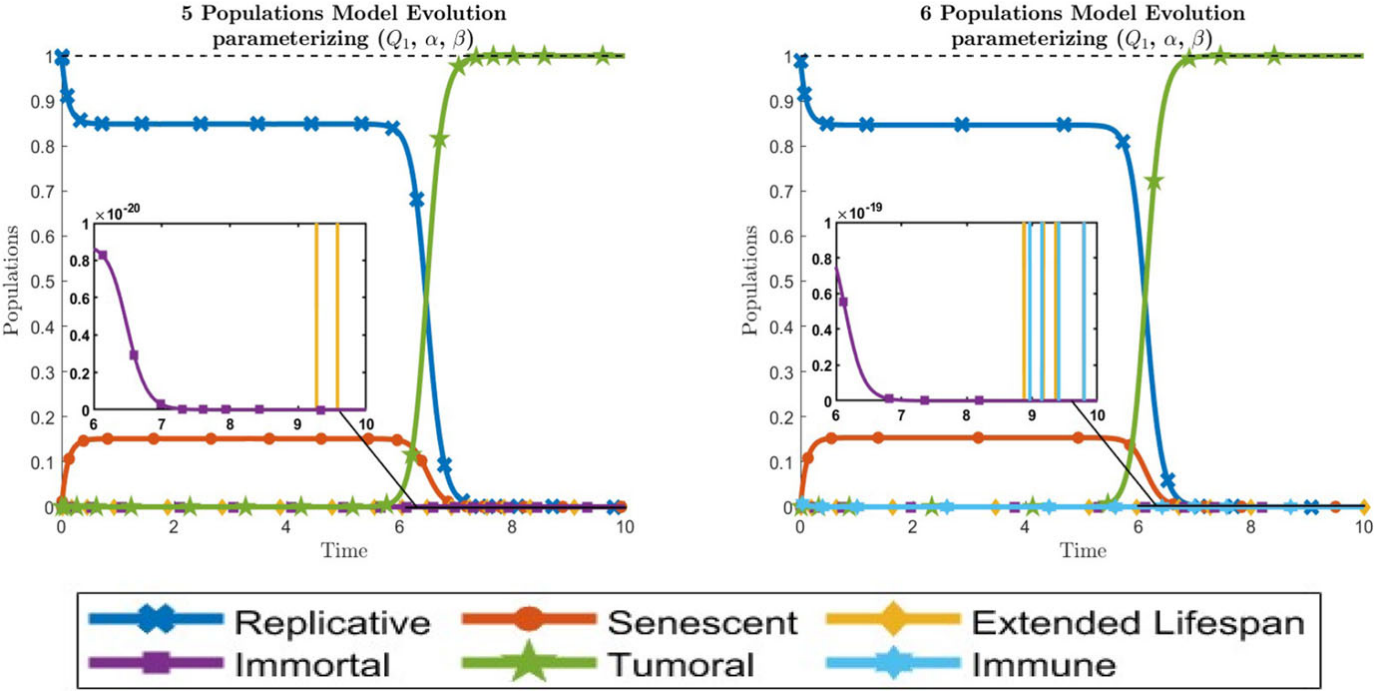
Dynamical behavior of cells for the optimal values given in Table 2 of the parameters $(Q_1, \alpha , \beta )$ causing the tumor invasion. The left graphic is for the 5-populations model whereas the right graphic is for the 6-populations model. Line codes, $y_1$: blue crossed, $y_2$: red circled, $y_3$: yellow diamonded, $y_4$: purple squared, $y_5$: green 5 points stared, $y_6$: cyan 6 points stared (color figure online)

This time, the optimal combination of parameters for each model (and the functional cost values) are given in Table 2 and the evolution of the populations for each model in Fig. 4 (the final values at $t=T$ of each population can be seen at Supplementary Material A.1).

**Table 2:** Optimal values of the identifiable transmission parameters and their functional cost

Model	${Q_1}$	${\alpha }$	${\beta }$	*J*
5 Pop.	0.84872	1–4.7$\cdot 10^{-11}$	1–4.7$\cdot 10^{-10}$	$1.7 \cdot 10^{-2}$
6 Pop.	0.84646	1–9.5$\cdot 10^{-11}$	1–2.7$\cdot 10^{-9}$	$1.5 \cdot 10^{-2}$

Contrary to the results presented in Table 1, since the non-identifiable $Q_4$ is now fixed, there are no significant variations in these optimal parameters

Comparing Figs. 3 and 4 and Tables 1 and 2, we conclude that there are not great differences between fittting $\left( Q_1, Q_3, Q_4, \alpha , \beta \right)$ or just fitting $\left( Q_1, \alpha , \beta \right)$, but with a saving of 1/10 in the computational cost.

## Introduction to therapies

Now, we introduce the following therapies to eradicate (or at least to palliate) the tumor:

$$\begin{aligned} \left\{ \begin{aligned} \pmb {T_1}:=&\, \text {Direct elimination of tumor cells}; \\ \pmb {T_2}:=&\, \text {Transformation from tumor cells to senescent ones}; \\ \pmb {T_3}:=&\, \text {Reinforcement of the immune cells (for the }6\text {-populations model)}. \\ \end{aligned} \right. \end{aligned}$$

### 5-populations model with two therapies

We introduce two dimensionless therapy parameters $\kappa _1\in (0,1)$ and $\kappa _2 = 1 - Q_5\in (0,1)$, modeling the effects of therapies $T_1$ and $T_2$ by means of the following modification of the 5-populations model:

21 $$\begin{aligned} \left\{ \begin{array}{rcl} y'_1 &{} = &{} (r_1Q_1 - E(\underline{y}))y_1 \\ y'_2 &{} = &{} - E(\underline{y})y_2 + \alpha r_1(1 - Q_1)y_1 + \beta r_3(1 - Q_3)y_3 \\ &{}&{}\quad + \left( 1 - \kappa _1 \right) r_5(y_2) ( 1 - Q_5 )y_5 \\ y'_3 &{} = &{} (r_3Q_3 - \delta _3 - E(\underline{y}))y_3 + (1 - \alpha )r_1(1 - Q_1)y_1 \\ y'_4 &{} = &{} (r_4Q_4 - \delta _4 - E(\underline{y}))y_4 + (1 - \beta )r_3(1 - Q_3)y_3 \\ y'_5 &{} = &{} ( \left( 1 - \kappa _1 \right) \left( r_5(y_2) Q_5 - \delta _5 \right) - E(\underline{y}))y_5 + r_4(1 - Q_4)y_4 \end{array} \right. \end{aligned}$$

where the auxiliary function $E ( \underline{y})$ changes by the influence of the $\kappa _1$ parameter:

22 $$\begin{aligned} E ( \underline{y}) = \sum _{i=1}^4 (r_i - \delta _i)y_i + \left( 1 - \kappa _1 \right) ( r_5( y_2 ) - \delta _5 )y_5. \end{aligned}$$

### 6-populations model with three therapies

In addition, we introduce the (dimensionless) parameter $\kappa _3 \in ( 0, 1 )$, modeling the therapy $T_3$, via the following 6-populations model:

23 $$\begin{aligned} \left\{ \begin{array}{rcl} y'_1 &{} = &{} (r_1Q_1 - E(t,\underline{y}))y_1 \\ y'_2 &{} = &{} - E(t,\underline{y})y_2 + \alpha r_1(1 - Q_1)y_1 + \beta r_3(1 - Q_3)y_3\\ &{}&{}\quad + \left( 1 - \kappa _1 \right) r_5(t, y_2, y_6) \left( 1 - Q_5 \right) y_5 \\ y'_3 &{} = &{} (r_3Q_3 - \delta _3 - E(t,\underline{y}))y_3 + (1 - \alpha )r_1(1 - Q_1)y_1 \\ y'_4 &{} = &{} (r_4Q_4 - \delta _4 - E(t,\underline{y}))y_4 + (1 - \beta )r_3(1 - Q_3)y_3 \\ y'_5 &{} = &{} (\left( 1 - \kappa _1 \right) \left( r_5(t, y_2, y_6) Q_5 - \delta _5 \right) - E(t,\underline{y}))y_5 + r_4(1 - Q_4)y_4 \\ y'_6 &{} = &{} (r_6(y_5, \kappa _3) - \delta _6(y_5) - E(t,\underline{y}))y_6 \\ \end{array} \right. \end{aligned}$$

where, due to the influence of therapy $T_3$, the functions $E( t, \underline{y})$, $r_5(t, y_2, y_6)$ and $r_6(y_5, \kappa _3 )$ must change as follows:

24 $$\begin{aligned} E( t, \underline{y})= & {} \sum _{i=1}^4 (r_i - \delta _i)y_i + \left( 1 - \kappa _1 \right) (r_5(t,y_2,y_6) - \delta _5 )y_5 \nonumber \\{} & {} + (r_6( y_5, \kappa _3 ) - \delta _6(y_5))y_6\,, \end{aligned}$$

25 $$\begin{aligned} r_5(t, y_2, y_6)= & {} r_5 \left( 1 + \rho \frac{y_2}{y_2 + 1} - \sigma \frac{T}{t + t^* + T} \frac{y_6}{y_6 + 1} \right) \,, \end{aligned}$$

26 $$\begin{aligned} r_6(y_5, \kappa _3 )= & {} r_6 \left( \frac{2y_5}{y_5 + 1} + \kappa _3 \right) \,. \end{aligned}$$

In (25), $t^*$ denotes the starting time for the application of the therapies.

### Fitting therapy parameters

We start the application of therapies at different times with the corresponding initial tumor (around $10 \%$, $25 \%$, $50 \%$ and $70 \%$ of the total population, respectively). In all cases, we set a desirable final population in which the tumor cells should remain in a insignificant amount (and also the cycling cells: extended lifespan and immortal cells). In fact, it will remain approximately $80\%$ of replicative cells and $20\%$ of senescent ones. More concretely, for the 6-populations model, we take:

$$\begin{aligned} \underline{y}_{d_{T}} = (0.8, 0.1879999, 10^{-7}, 10^{-3}, 10^{-3}, 0.01)^t \end{aligned}$$

and the immune percentage adds to the senescent population at the 5-populations model.

For both models we fit the therapy parameters by using an optimal control problem with a cost function ($J = J \left( \kappa _1, \kappa _2 \right)$ and $J = J \left( \kappa _1, \kappa _2, \kappa _3 \right)$ for the 5 and 6-populations models, respectively). This cost function is defined by a least square distance through time of the linear progression between the initial state and the desirable final state $\underline{y}_{d_{T}}$. We consider the following ranges for the 3 therapy parameters:

$$\begin{aligned} \left\{ \begin{array}{rcrclrrr} \pmb {T_1} &{}:= &{} \kappa _1 &{} \in &{} [ &{} \varepsilon , &{} 1 - \varepsilon &{} ], \\ \pmb {T_2} &{}:= &{} 1 - Q_5 \equiv \kappa _2 &{} \in &{} [ &{} \varepsilon , &{} 1 - \varepsilon &{} ], \\ \pmb {T_3} &{}:= &{} \kappa _3 &{} \in &{} [ &{} \varepsilon , &{} 1 - \varepsilon &{} ]. \\ \end{array} \right. \end{aligned}$$

**Table 3:** Optimal values of the therapy parameters and their functional cost

Init. tumor	Model	${\% T_1}$	${\% T_2}$	${\% T_3}$	*J*
${y_{0_5} \approx 0.1}$	5 Pop.	$36.04 \%$	$63.96 \%$	–	$8.5249 \cdot 10^{-5}$
	6 Pop.	$15.16 \%$	$22.44 \%$	$62.40 \%$	$2.0372 \cdot 10^{-4}$
${y_{0_5} \approx 0.25}$	5 Pop.	$57.30 \%$	$42.70 \%$	–	$1.182 \cdot 10^{-4}$
	6 Pop.	$25.90 \%$	$14.76 \%$	$59.34 \%$	$1.3401 \cdot 10^{-3}$
${y_{0_5} \approx 0.5}$	5 Pop.	$69.84 \%$	$30.16 \%$	–	$3.0848 \cdot 10^{-4}$
	6 Pop.	$30.72 \%$	$12.92 \%$	$56.36 \%$	$3.9927 \cdot 10^{-3}$
${y_{0_5} \approx 0.7}$	5 Pop.	$74.67 \%$	$25.33 \%$	–	$3.7913 \cdot 10^{-4}$
	6 Pop.	$40.55 \%$	$3.33 \%$	$56.12 \%$	$6.0015 \cdot 10^{-3}$

where $\varepsilon = 10^{-2}$. We take 9 different values for the initialization of each therapy parameter, resulting $9^{2} = 81$ and $9^{3} = 729$ combinations for the 5 and 6-populations models, respectively.

**Fig. 5:**
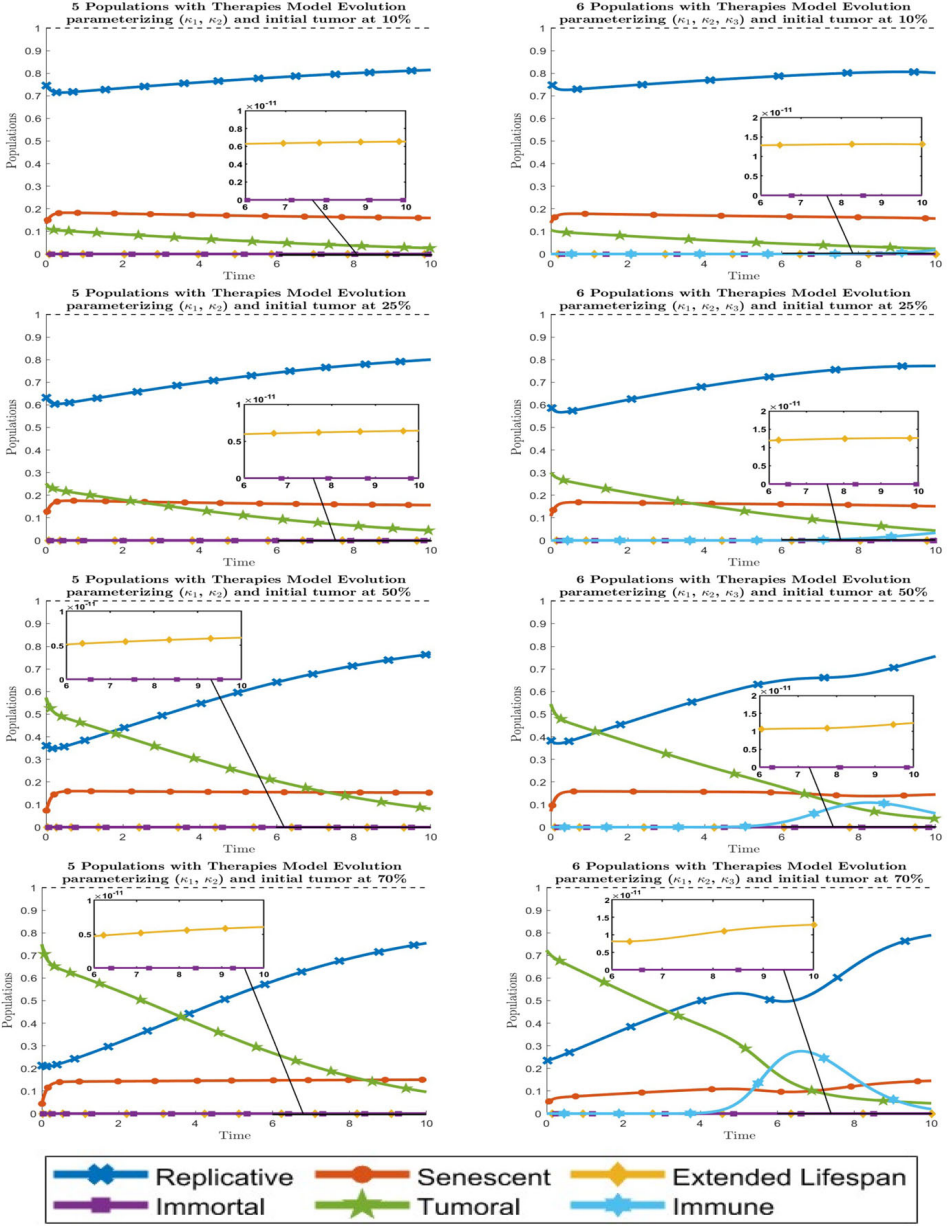
Dynamical behavior of all cell types with optimal therapies. By columns, the left graphic is for the 5-populations model whereas the right one is for the 6-populations model. By rows, the graphics are for the initial tumor $\approx 10\%$, $25\%$, $50\%$ and $70\%$ respectively from top to bottom. Line codes, $y_1$: blue crossed, $y_2$: red circled, $y_3$: yellow diamonded, $y_4$: purple squared, $y_5$: green 5 points stared, $y_6$: cyan 6 points stared (color figure online)

The optimal combination of therapies and its cost function are given in Table 3. The value $t^*$ for the time when therapies started, as well as the initial and the final values of each population, can be seen at Supplementary Material A.2. In view of Table 3, when the initial tumor increases the optimal value of therapy $T_1$ increases whereas the optimal values of $T_2$ and $T_3$ decrease. Moreover, the therapy strengthening immune cells $T_3$ is always the most effective, and secondly the therapy passing tumor cells to senescent ones $T_2$ is desirable for early states of the tumor, while the therapy eradicating directly tumor cells $T_1$ is desirable for more advanced tumors. The evolution of the populations with optimal therapies for each model can be seen in Fig. 5. We remark that the behavior of the cells at each initial tumor stage is rather linear, as intended in the fitting process. But the behaviour for the 6-populations model is somewhat nonlinear, due to the inclusion of the immune population. This nonlinear effect becomes stronger the larger the initial tumor.

## Therapy parameters’ identifiability

Now we study the identifiability of the optimal therapy parameters listed in Table 3 for both models applying both methods (*PCA* and *Eigenvalues*). Similarly to Sect. 4, we consider 4 different stages of the initial tumor ($\approx$ $10\%$, $25\%$, $50\%$ and $70\%$, respectively).

### 5-populations model

The eigenvectors and eigenvalues for the 5-populations model can be found in Supplementary Material C.2.1. Looking at these values, we observe the same pattern for both *PCA* and Eigenvalues methods, reaching the following conclusions:

- For the initial tumor $\approx 10\%$: For all populations, $\kappa _2$ is the most identifiable parameter. However, by observing the components of each eigenvector associated to each eigenvalue we could deduce both therapies are quite identifiable.
- For the initial tumor $\approx 25\%$: Unlike the previous case, $\kappa _1$ is the most identifiable parameter for all populations, although the difference between them is again small.
- For the initial tumor $\approx 50\%$ and $\approx 70\%$: For all populations, $\kappa _1$ is the most identifiable parameter, but this time, the scales of the eigenvector components are different (except for $y_2$ in the second case).

The final conclusion is that therapy $T_2$ is the most identifiable when the initial tumor is small, while $T_1$ is more identifiable the later the tumor invasion is detected.

**Table 4:** Definition of the new therapy parameters $\chi _i$ as linear combinations of the original therapy parameters $\kappa _i$ via the associated eigenvectors

		5-Pop.		6-Pop.		
$y_{0_5} \approx 0.1$	${\chi _i}$	$\chi _1 = \pmb {-7.6 \cdot 10^{ -1} \kappa _1} + 6.4 \cdot 10^{ -1} \kappa _2$		$\chi _1 = \pmb {7.4 \cdot 10^{ -1} \kappa _1} -6.6 \cdot 10^{ -1} \kappa _2 -5.2 \cdot 10^{ -2} \kappa _3$		
		$\chi _2 = 6.4 \cdot 10^{ -1} \kappa _1 \pmb {+ 7.6 \cdot 10^{ -1} \kappa _2}$		$\chi _2 =6.6 \cdot 10^{ -2} \kappa _1 -3.9 \cdot 10^{ -3} \kappa _2 \pmb {+ 9.9 \cdot 10^{ -1} \kappa _3}$		
				$$\chi _3 = -6.6 \cdot 10^{ -1} \kappa _1 \pmb {-7.4 \cdot 10^{ -1} \kappa _2} + 4.1 \cdot 10^{ -2} \kappa _3$$		
	${\lambda _i}$	$\left| \lambda _1 \right| = 3.0 \cdot 10^{ -3}$	$\left| \lambda _2 \right| = 3.5 \cdot 10^{ 1}$	$\left| \lambda _1 \right| = 2.4 \cdot 10^{ -3}$	$\left| \lambda _2 \right| = 1.2 \cdot 10^{ -1}$	$\left| \lambda _3 \right| = 3.9 \cdot 10^{ 1}$
$y_{0_5} \approx 0.25$	${\chi _i}$	$\chi _1 = 6.4 \cdot 10^{ -1} \kappa _1 \pmb {-7.6 \cdot 10^{ -1} \kappa _2}$		$\chi _1 = 5.9 \cdot 10^{ -1} \kappa _1 \pmb {-7.9 \cdot 10^{ -1} \kappa _2} -8.2 \cdot 10^{ -2} \kappa _3$		
		$\chi _2 = \pmb {-7.6 \cdot 10^{-1} \kappa _1} -6.4 \cdot 10^{ -1} \kappa _2$		$\chi _2 = 9.1 \cdot 10^{ -2} \kappa _1 -3.4 \cdot 10^{-2} \kappa _2 \pmb {+ 9.9 \cdot 10^{ -1} \kappa _3}$		
				$$\chi _3 = \pmb {-7.9 \cdot 10^{ -1} \kappa _1} -6.0 \cdot 10^{ -1} \kappa _2 + 5.1 \cdot 10^{ -2} \kappa _3$$		
	${\lambda _i}$	$\left| \lambda _1 \right| = 1.0 \cdot 10^{-2}$	$\left| \lambda _2 \right| = 7.3 \cdot 10^{ 1}$	$\left| \lambda _1 \right| = 1.2 \cdot 10^{ -2}$	$\left| \lambda _2 \right| = 3.0 \cdot 10^{ -1}$	$\left| \lambda _3 \right| = 2.2 \cdot 10^{ 2}$
$y_{0_5} \approx 0.5$	${\chi _i}$	$\chi _1 = \,\, 5.4 \cdot 10^{ -1} \kappa _1 \pmb {-8.4 \cdot 10^{ -1} \kappa _2}$		$\chi _1 = 5.4 \cdot 10^{ -1} \kappa _1 \pmb {-8.3 \cdot 10^{ -1} \kappa _2} -9.963 \cdot 10^{ -2} \kappa _3$		
		$\chi _2 = \pmb {-8.4 \cdot 10^{-1} \kappa _1} -5.4 \cdot 10^{ -1} \kappa _2$		$\chi _2 = 1.1 \cdot 10^{ -1} \kappa _1 -4.7 \cdot 10^{ -2} \kappa _2 \pmb {+ 9.9 \cdot 10^{ -1} \kappa _3}$		
				$$\chi _3 = \pmb {-8.3 \cdot 10^{ -1} \kappa _1} -5.4 \cdot 10^{ -1} \kappa _2 + 6.5 \cdot 10^{ -2} \kappa _3$$		
	${\lambda _i}$	$\left| \lambda _1 \right| = 4.6 \cdot 10^{-2}$	$\left| \lambda _2 \right| = 1.8 \cdot 10^{ 2}$	$\left| \lambda _1 \right| = 3.5 \cdot 10^{ -2}$	$\left| \lambda _2 \right| = 5.9 \cdot 10^{ -1}$	$\left| \lambda _3 \right| = 9.4 \cdot 10^{ 2}$
$y_{0_5} \approx 0.7$	${\chi _i}$	$\chi _1 = \,\, 5.0 \cdot 10^{ -1} \kappa _1 \pmb {-8.6 \cdot 10^{ -1} \kappa _2}$		$\chi _1 = 4.2 \cdot 10^{ -1} \kappa _1 \pmb {-9.0 \cdot 10^{ -1} \kappa _2} -2.9 \cdot 10^{ -2} \kappa _3$		
		$\chi _2 = \pmb {-8.6 \cdot 10^{-1} \kappa _1} -5.0 \cdot 10^{ -1} \kappa _2$		$\chi _2 =8.5 \cdot 10^{ -2} \kappa _1 + 7.4 \cdot 10^{-3} \kappa _2 \pmb {+ 9.9 \cdot 10^{ -1} \kappa _3}$		
				$$\chi _3 = \pmb {-9.0 \cdot 10^{ -1} \kappa _1} -4.2 \cdot 10^{ -1} \kappa _2 + 8.0 \cdot 10^{ -2} \kappa _3$$		
	${\lambda _i}$	$\left| \lambda _1 \right| = 7.9 \cdot 10^{ -2}$	$\left| \lambda _2 \right| = 2.2 \cdot 10^{ 2}$	$\left| \lambda _1 \right| =5.2 \cdot 10^{ -2}$	$\left| \lambda _2 \right| = 7.7 \cdot 10^{ -1}$	$\left| \lambda _3 \right| = 3.9 \cdot 10^{ 2}$

In each case, the most identifiable therapy is written in bold

### 6-populations model

The eigenvectors and eigenvalues for this model can be seen at Supplementary Material C.2.2. We observe again the same pattern for both *PCA* and Eigenvalue methods, arriving at the following conclusions:

- For the initial tumor $\approx 10\%$: For all populations, $\kappa _2$ is the most identifiable and $\kappa _1$ the most non-identifiable, although all three therapies are quite identifiable acting separately.
- For the initial tumor $\approx 25\%$, $\approx 50\%$ and $\approx 70\%$: Unlike the previous case, $\kappa _1$ is the most identifiable and $\kappa _2$ the most non-identifiable for all populations (except for $y_2$), although the three therapies are again identifiable acting separately.

Moreover, therapy $T_1$ becomes more identifiable the higher the initial tumor value.

## Application of linear combinations of therapies

According to the previous Section, none of the therapy parameters are non-identifiable acting separately. But, if we look at the eigenvalues and eigenvectors of the applied identifiability methods, there is always one eigenvector direction of perturbation (even two in some cases) of the optimal combination of therapies that is completely non-identifiable. This leads us to the idea of considering new therapy parameters as linear combinations of the original ones via the eigenvectors of the *Eigenvalues Method*. With this idea, we will obtain perturbations of the optimal therapy maintaining the (optimal) results. These new therapy parameters (that we will denote as $\chi _i$ from now on) are linear combinations of the original therapy parameters $\kappa _i$, and they are given in Table 4 (as well as the corresponding eigenvalues).

In view of Table 4, we can reach the following conclusions:

- For the 5-populations model, the most non-identifiable parameter $\chi _1$ is the combination of both therapies $T_1$ and $T_2$ (one countering the other), whose $T_1$ component decreases as the initial tumor increases whereas the $T_2$ component increases. The contrary occurs for the most identifiable parameter $\chi _2$, combining both therapies $T_1$ and $T_2$.
- For the 6-populations model, the behavior of both the most and the least identifiable parameters is analogous to the 5-populations model, but having a little presence of $T_3$ in both of them. In addition, the middle identifiable therapy is mainly $T_3$.

Now, we fit the modified therapy parameters $\chi _i$, verifying that by fixing at least the most non-identifiable parameter $\chi _1$, the (optimal) results are practically unchanged.

### 5-populations model

First of all, taking into account that $\kappa _i \in ( 0, 1 )$, $i=1,2$, we define the domain of each new therapy parameter $\chi _i$ at each tumor initial value, computing the minimum and maximum value of $\chi _i$ as $\kappa _1,\kappa _2\in [0,1]$ (in fact, these values are the minimum and maximum of $(\kappa _1,\kappa _2)$ in vertices (0, 0), (0, 1), (1, 0), (1, 1), see Table 5).

**Table 5:** Ranges of the new therapy parameters $\chi _i$ for the 5-populations model

	${\chi _1}$				${\chi _2}$			
${y_{0_5} \approx 0.1}$	[	$-7.6436 \cdot 10^{ -1},$	$6.4479 \cdot 10^{ -1}$	]	[	0,	$1.4091 \cdot 10^{ 0}$	]
${y_{0_5} \approx 0.25}$	[	$-7.6765 \cdot 10^{ -1},$	$6.4086 \cdot 10^{ -1}$	]	[	$-1.4085 \cdot 10^{ 0},$	0	]
${y_{0_5} \approx 0.5}$	[	$-8.3872 \cdot 10^{ -1},$	$5.4456 \cdot 10^{ -1}$	]	[	$-1.3833 \cdot 10^{ 0},$	0	]
${y_{0_5} \approx 0.7}$	[	$-8.6483 \cdot 10^{ -1},$	$5.0207 \cdot 10^{ -1}$	]	[	$-1.3669 \cdot 10^{ 0},$	0	]

We now fit these new therapy parameters as in Sect. 5. Furthermore, as we know that the identifiability of these new therapy parameters is ascending the higher is their index, we no only fit the best combination of $(\chi _1, \chi _2)$ but we also fit the best $\chi _2$ after fixing $\chi _1$ to different values. To do so, we take the values 0 or the middle value of its range at each case (that we will denote as ${\tilde{\chi }}_1$ from now on).

We take again 9 different values of each parameter as initialization, resulting $9^{2} = 81$ or $9^{1} = 9$ combinations, when 2 or 1 parameter are fitted, respectively.

The best combinations of new therapy parameters for each case and for each tumor initial value (as well as the cost function) are given in Table 6. We remark that, we obtain practically the same results in all cases, which justifies the reduction from 2 to 1 in the number of therapies to fit. The final values of each population for each tumor initial value can be found in Supplementary Material A.2 and A.3.1, respectively.

**Table 6:** Optimal values of the new therapy parameters and their functional cost for the 5-populations model

		${\chi _1}$	${\chi _2}$	*J*
${y_{0_5} \approx 0.1}$	${( \chi _1, \chi _2 )}$	$7.6387 \cdot 10^{ -2}$	$3.9862 \cdot 10^{ -1}$	$8.5249 \cdot 10^{ -5}$
	${( 0, \chi _2 )}$	0	$3.9603 \cdot 10^{ -1}$	$8.948 \cdot 10^{ -5}$
	${( {\tilde{\chi }}_1, \chi _2 )}$	$-5.9786 \cdot 10^{ -2}$	$3.9008 \cdot 10^{ -1}$	$9.8547 \cdot 10^{ -5}$
${y_{0_5} \approx 0.25}$	${( \chi _1, \chi _2 )}$	$2.2114 \cdot 10^{ -2}$	$-4.002 \cdot 10^{ -1}$	$1.182 \cdot 10^{ -4}$
	${( 0, \chi _2 )}$	0	$-3.9994 \cdot 10^{ -1}$	$1.1934 \cdot 10^{ -4}$
	${( {\tilde{\chi }}_1, \chi _2 )}$	$-6.3395 \cdot 10^{ -2}$	$-3.9663 \cdot 10^{ -1}$	$1.3577 \cdot 10^{ -4}$
${y_{0_5} \approx 0.5}$	${( \chi _1, \chi _2 )}$	$7.0146 \cdot 10^{ -2}$	$-4.1282 \cdot 10^{ -1}$	$3.0848 \cdot 10^{ -4}$
	${( 0, \chi _2 )}$	0	$-4.1061 \cdot 10^{ -1}$	$3.6264 \cdot 10^{ -4}$
	${( {\tilde{\chi }}_1, \chi _2 )}$	$-1.4708 \cdot 10^{ -1}$	$-3.9051 \cdot 10^{ -1}$	$8.4001 \cdot 10^{ -4}$
${y_{0_5} \approx 0.7}$	${( \chi _1, \chi _2 )}$	$8.4923 \cdot 10^{ -2}$	$-4.2109 \cdot 10^{ -1}$	$3.7913 \cdot 10^{ -4}$
	${( 0, \chi _2 )}$	0	$-4.1792 \cdot 10^{ -1}$	$5.1798 \cdot 10^{ -4}$
	${( {\tilde{\chi }}_1, \chi _2 )}$	$-1.8138 \cdot 10^{ -1}$	$-3.8706 \cdot 10^{ -1}$	$1.7195 \cdot 10^{ -3}$

Making the corresponding change of variables, we give the combinations of original therapy parameters $\kappa _i$ at Supplementary material, C.2.1, in Table 14.

**Fig. 6:**
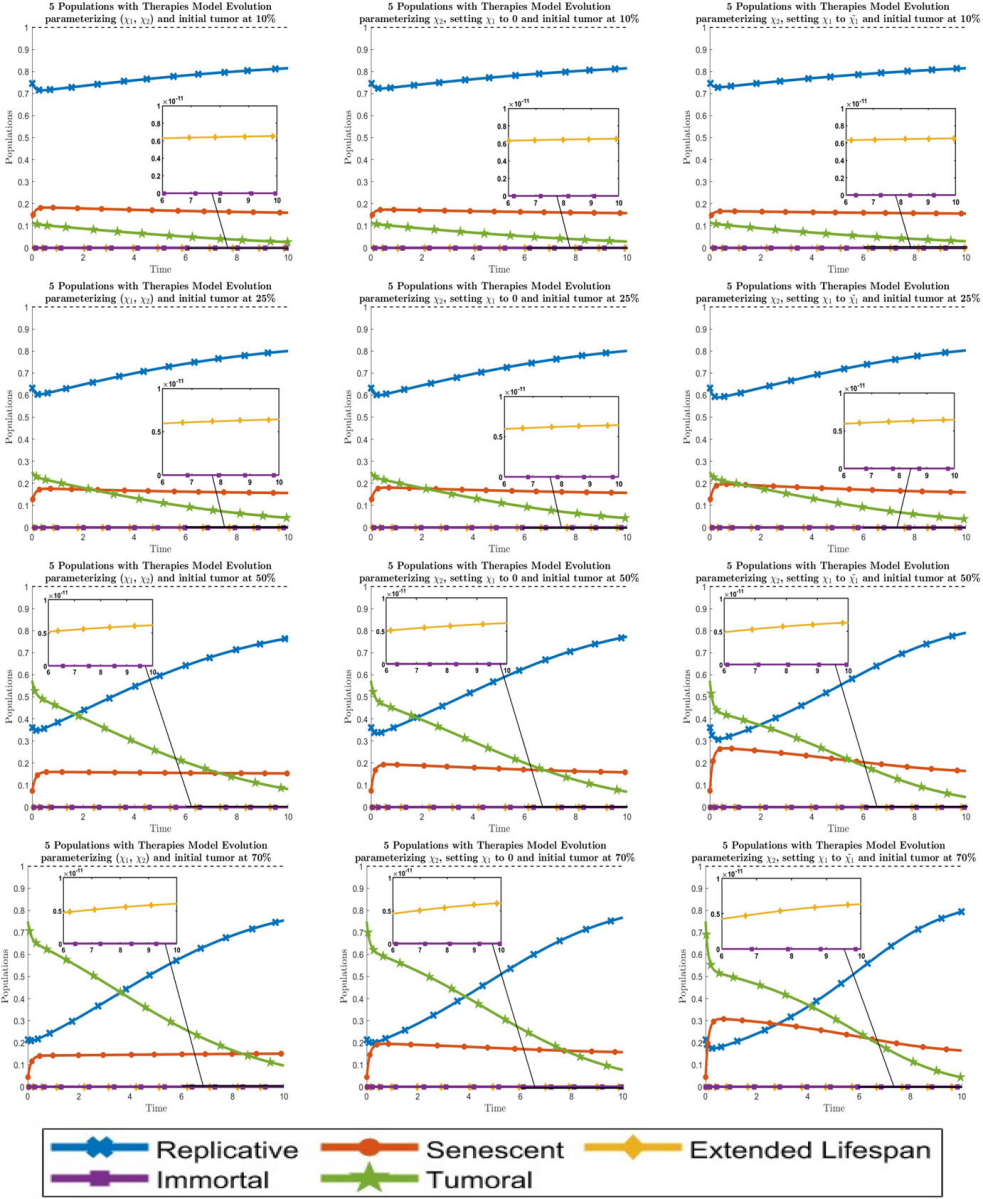
Dynamical behavior of the 5 cell types with the optimal values of the new therapy parameters $\chi _i$. By columns, the left one is when both parameters are fitted, the middle one is when $\chi _1$ is set to 0 and the right one is when $\chi _1$ is set to ${\tilde{\chi }}_1$. By rows, the graphics are for the initial values of the tumor population $\approx 10 \%$, $25 \%$, $50 \%$ and $70 \%$ respectively from top to bottom. Line codes, $y_1$: blue crossed, $y_2$: red circled, $y_3$: yellow diamonded, $y_4$: purple squared, $y_5$: green 5 points stared (color figure online)

The evolution of the 5-populations with optimal therapies can be seen in Fig. 6. Comparing results in Fig. 6 when $\chi _1$ is fixed, we see that the choice $\chi _1 = 0$ is clearly better than $\chi _1 = {\tilde{\chi }}_1$. One possible explanation for this is that the identifiability of the parameter $\chi _1$ for all the variants (which are detected by their respectively eigenvalues) is almost zero.

### 6-populations model

Similarly to the 5-populations model, the ranges of the new therapy parameter $\chi _i$ at each tumor initial value are given in Table 7.

**Table 7:** Ranges of the new therapy parameters $\chi _i$ for the 6-populations model

Init. tumor	${\chi _1}$	${\chi _2}$	${\chi _3}$
${y_{0_5} \approx 0.1}$	$[-7.2 \cdot 10^{ -1},7.4 \cdot 10^{ -1}]$	$[-3.9 \cdot 10^{ -3},1.1 \cdot 10^{ 0}]$	$[-1.4 \cdot 10^{ 0},4.1 \cdot 10^{ -2}]$
${y_{0_5} \approx 0.25}$	$[-8.8 \cdot 10^{ -1},5.9 \cdot 10^{ -1}]$	$-3.5 \cdot 10^{ -2},1.1 \cdot 10^{ 0}]$	$[-1.4 \cdot 10^{ 0},5.1 \cdot 10^{ -2}]$
${y_{0_5} \approx 0.5}$	$[-9.3 \cdot 10^{ -1},5.4 \cdot 10^{ -1}]$	$[-4.8 \cdot 10^{ -2},1.102 \cdot 10^{ 0}]$	$[-1.4 \cdot 10^{ 0},6.5 \cdot 10^{ -2}]$
${y_{0_5} \approx 0.7}$	$[-9.3 \cdot 10^{ -1},4.2 \cdot 10^{ -1}]$	$[0\cdot 10^{ 0}, 1.1 \cdot 10^{ 0}]$	$[-1.3 \cdot 10^{ 0}, 8.0 \cdot 10^{ -2}]$

We study different situations: the 3 parameters one, fitting the best combination of $(\chi _1, \chi _2, \chi _3)$; the 2 parameters ones, fitting $(\chi _2, \chi _3)$ with different fixed values of $\chi _1$ (0 and ${\tilde{\chi }}_1$) and the 1 parameter case, fitting $\chi _3$ once fixed $(\chi _1, \chi _2)$ to different values: (0, 0), $(0, {\tilde{\chi }}_2)$, $({\tilde{\chi }}_1, 0)$ and $({\tilde{\chi }}_1, {\tilde{\chi }}_2)$.

We again take 9 different values for the initialization of each parameter, considering $9^{3} = 729$, $9^{2} = 81$ and $9^{1} = 9$ combinations when we fit 3, 2 or 1 parameter, respectively.

The best combinations of new therapy parameters $\chi _i$ (as well as the cost function) are given in Table 8 (where again the fixed parameters are written slightly lighter). The corresponding combinations of original therapy parameters $\kappa _i$ are given at Supplementary Material C.2.2, Table 15 (of course, in the 3 parameters setting, the same results as in Sect. 5 are obtained). We remark that, in the 1 parameter case, by taking the values $(\chi _1,\chi _2)=(0, 0)$ and $(\chi _1,\chi _2)=({\tilde{\chi }}_1, 0)$, the problem became infeasible, hence we can not take $\chi _2$ to 0.

**Table 8:** Optimal values of the new therapy parameters $\chi _i$ and their functional cost *J* for the 6-populations model

		${\chi _1}$	${\chi _2}$	${\chi _3}$	*J*
${y_{0_5} \approx 0.1}$	${( \chi _1, \chi _2, \chi _3 )}$	$-1.0301 \cdot 10^{ -1}$	$9.3763 \cdot 10^{ -1}$	$-3.5966 \cdot 10^{ -1}$	$2.0372 \cdot 10^{ -4}$
	${( 0, \chi _2, \chi _3 )}$	0	$9.3992 \cdot 10^{ -1}$	$-3.5463 \cdot 10^{ -1}$	$2.0871 \cdot 10^{ -4}$
	${( {\tilde{\chi }}_1, \chi _2, \chi _3 )}$	$1.2322 \cdot 10^{ -2}$	$9.54 \cdot 10^{ -2}$	$-3.9217 \cdot 10^{ -1}$	$2.0975 \cdot 10^{ -4}$
	${( 0, 0, \chi _3 )}$	0	0	–	–
	${( 0, {\tilde{\chi }}_2, \chi _3 )}$	0	$5.3024 \cdot 10^{ -1}$	$-3.7334 \cdot 10^{ -1}$	$2.1052 \cdot 10^{ -4}$
	${( {\tilde{\chi }}_1, 0, \chi _3 )}$	$1.2322 \cdot 10^{ -2}$	0	–	–
	${( {\tilde{\chi }}_1, {\tilde{\chi }}_2, \chi _3 )}$	$1.2322 \cdot 10^{ -2}$	$5.3024 \cdot 10^{ -1}$	$-3.7231 \cdot 10^{ -1}$	$2.1155 \cdot 10^{ -4}$
${y_{0_5} \approx 0.25}$	${( \chi _1, \chi _2, \chi _3 )}$	$-1.6002 \cdot 10^{ -2}$	$8.3488 \cdot 10^{ -1}$	$-3.6273 \cdot 10^{ -1}$	$1.3401 \cdot 10^{ -3}$
	${( 0, \chi _2, \chi _3 )}$	0	$8.3514 \cdot 10^{ -1}$	$-3.6262 \cdot 10^{ -1}$	$1.3407 \cdot 10^{ -3}$
	${( {\tilde{\chi }}_1, \chi _2, \chi _3 )}$	$-1.4057 \cdot 10^{ -1}$	$8.3509 \cdot 10^{ -1}$	$-3.5543 \cdot 10^{ -1}$	$1.3786 \cdot 10^{ -3}$
	${( 0, 0, \chi _3 )}$	0	0	–	–
	${( 0, {\tilde{\chi }}_2, \chi _3 )}$	0	$5.256 \cdot 10^{ -1}$	$-3.7846 \cdot 10^{ -1}$	$1.3643 \cdot 10^{ -3}$
	${( {\tilde{\chi }}_1, 0, \chi _3 )}$	$-1.4057 \cdot 10^{ -1}$	0	–	–
	${( {\tilde{\chi }}_1, {\tilde{\chi }}_2, \chi _3 )}$	$-1.4057 \cdot 10^{ -1}$	$5.256 \cdot 10^{ -1}$	$-3.6781 \cdot 10^{ -1}$	$1.4252 \cdot 10^{ -3}$
${y_{0_5} \approx 0.5}$	${( \chi _1, \chi _2, \chi _3 )}$	$2.4564 \cdot 10^{ -3}$	$7.4186 \cdot 10^{ -1}$	$-3.6677 \cdot 10^{ -1}$	$3.9927 \cdot 10^{ -3}$
	${( 0, \chi _2, \chi _3 )}$	0	$7.4186 \cdot 10^{ -1}$	$-3.6677 \cdot 10^{ -1}$	$3.9928 \cdot 10^{ -3}$
	${( {\tilde{\chi }}_1, \chi _2, \chi _3 )}$	$-1.9749 \cdot 10^{ -1}$	$7.4943 \cdot 10^{ -1}$	$-3.4815 \cdot 10^{ -1}$	$4.3268 \cdot 10^{ -3}$
	${( 0, 0, \chi _3 )}$	0	0	–	–
	${( 0, {\tilde{\chi }}_2, \chi _3 )}$	0	$5.2711 \cdot 10^{ -1}$	$-3.8004 \cdot 10^{ -1}$	$4.3838 \cdot 10^{ -3}$
	${( {\tilde{\chi }}_1, 0, \chi _3 )}$	$-1.9749 \cdot 10^{ -1}$	0	–	–
	${( {\tilde{\chi }}_1, {\tilde{\chi }}_2, \chi _3 )}$	$-1.9749 \cdot 10^{ -1}$	$5.2711 \cdot 10^{ -1}$	$-3.5732 \cdot 10^{ -1}$	$4.7991 \cdot 10^{ -3}$
${y_{0_5} \approx 0.7}$	${( \chi _1, \chi _2, \chi _3 )}$	$1.4222 \cdot 10^{ -1}$	$6.7159 \cdot 10^{ -1}$	$-3.786 \cdot 10^{ -1}$	$6.0016 \cdot 10^{ -3}$
	${( 0, \chi _2, \chi _3 )}$	0	$6.7575 \cdot 10^{ -1}$	$-3.7112 \cdot 10^{ -1}$	$6.2225 \cdot 10^{ -3}$
	${( {\tilde{\chi }}_1, \chi _2, \chi _3 )}$	$-2.5444 \cdot 10^{ -1}$	$7.3344 \cdot 10^{ -1}$	$-3.0703 \cdot 10^{ -1}$	$7.6647 \cdot 10^{ -3}$
	${( 0, 0, \chi _3 )}$	0	0	–	–
	${( 0, {\tilde{\chi }}_2, \chi _3 )}$	0	$5.4455 \cdot 10^{ -1}$	$-3.8172 \cdot 10^{ -1}$	$7.606 \cdot 10^{ -3}$
	${( {\tilde{\chi }}_1, 0, \chi _3 )}$	$-2.5444 \cdot 10^{ -1}$	0	–	–
	${( {\tilde{\chi }}_1, {\tilde{\chi }}_2, \chi _3 )}$	$-2.5444 \cdot 10^{ -1}$	$5.4455 \cdot 10^{ -1}$	$-3.1794 \cdot 10^{ -1}$	$8.8578 \cdot 10^{ -3}$

The fixed parameters are written slightly lighter

**Fig. 7:**
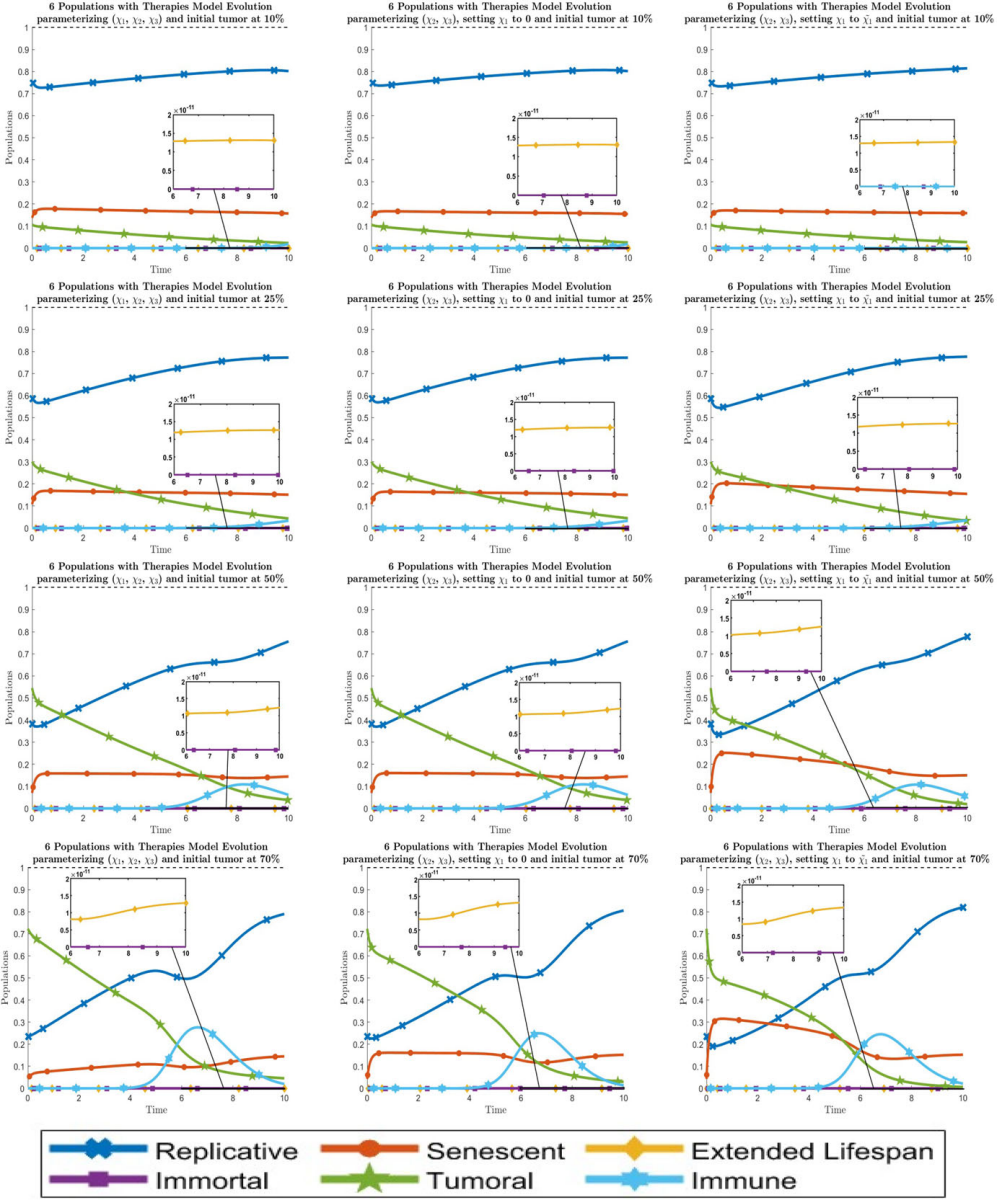
Dynamical behavior of all six cell types with the optimal values of the new therapy parameters $\chi _i$. By columns, the left one is when all parameters are fitted, the middle one is when $\chi _1$ is set to 0 and the right one is when $\chi _1$ is set to ${\tilde{\chi }}_1$. By rows, the graphics are for the initial values of the tumor population $\approx 10 \%$, $25 \%$, $50 \%$ and $70 \%$ respectively from top to bottom. Line codes, $y_1$: blue crossed, $y_2$: red circled, $y_3$: yellow diamonded, $y_4$: purple squared, $y_5$: green 5 points stared, $y_6$: cyan 6 points stared (color figure online)

**Fig. 8:**
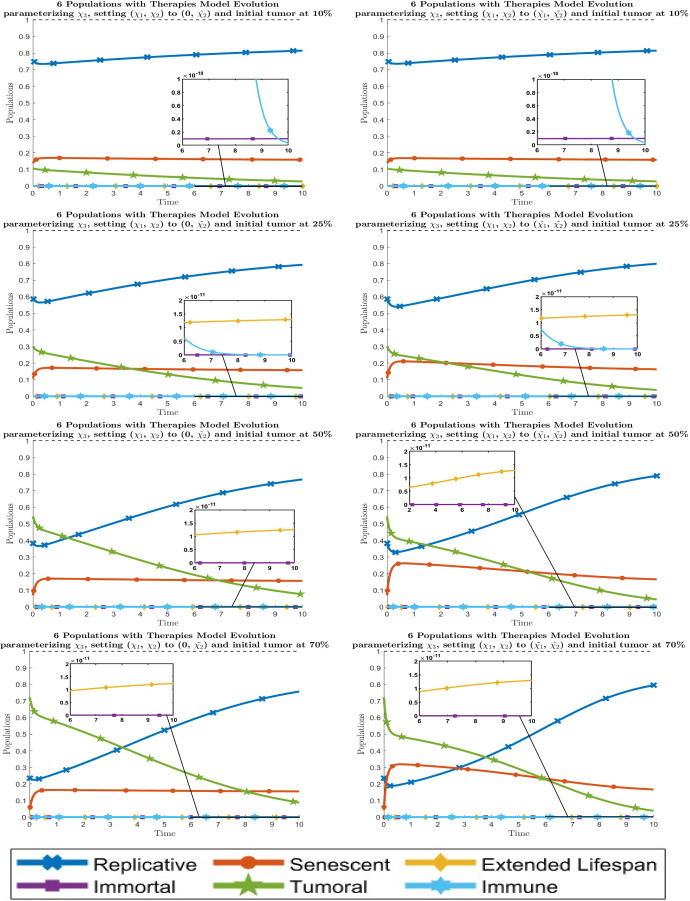
Dynamical behavior of all six cell types with the optimal values of the new therapy parameters $\chi _i$. By columns, the graphics are for setting the parameters $( \chi _1, \chi _2 )$ to $( 0, {\tilde{\chi }}_2 )$ and $( {\tilde{\chi }}_1, {\tilde{\chi }}_2 )$ respectively from left to right. By rows, the graphics are for the initial values of the tumor population $\approx 10 \%$, $25 \%$, $50 \%$ and $70 \%$ respectively from top to bottom. Line codes, $y_1$: blue crossed, $y_2$: red circled, $y_3$: yellow diamonded, $y_4$: purple squared, $y_5$: green 5 points stared, $y_6$: cyan 6 points stared (color figure online)

**Fig. 9:**
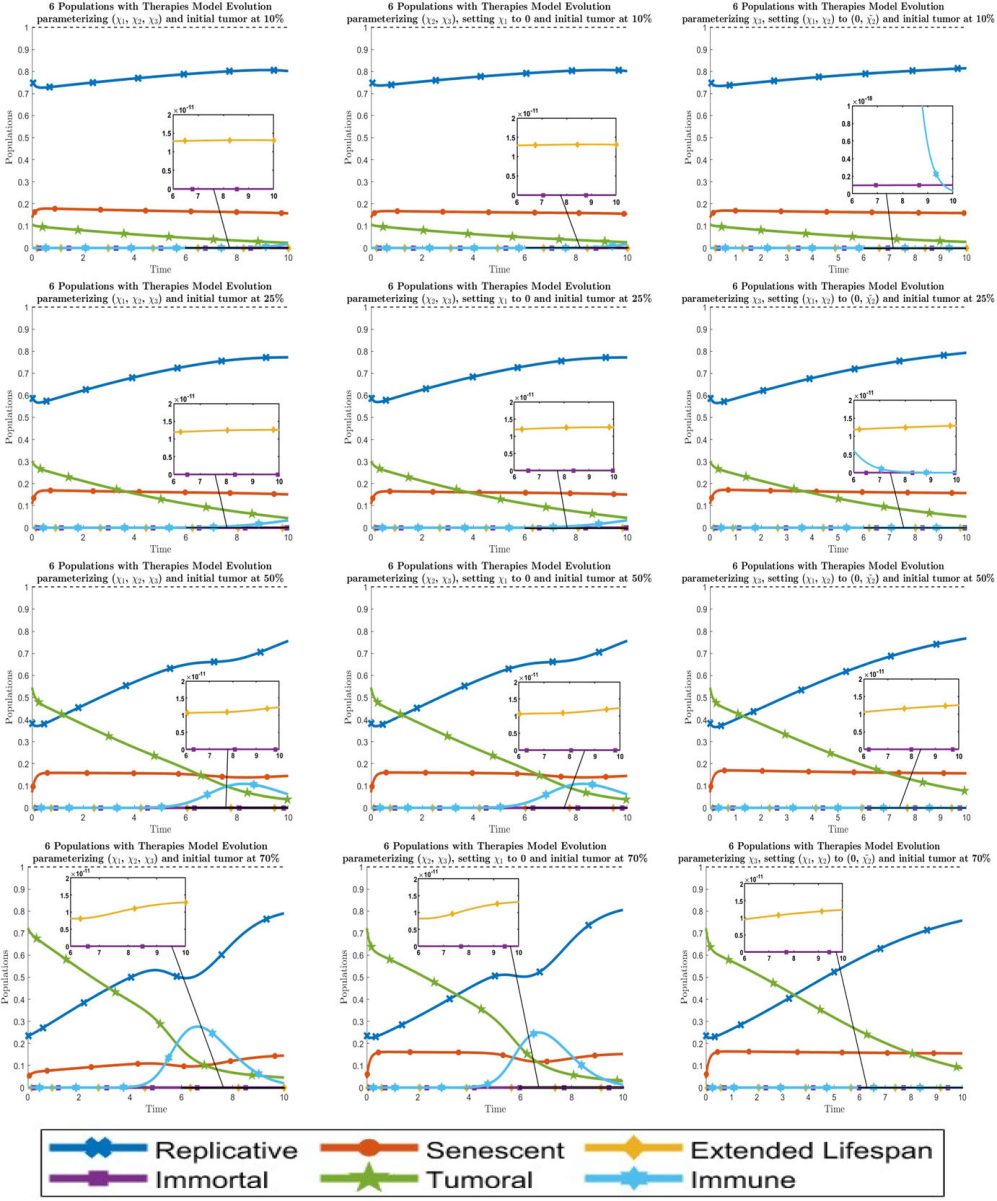
Dynamical behavior of all six cell types with the optimal values of the new therapy parameters $\chi _i$. By columns, the left one is when all parameters are fitted, the middle one is when $\chi _1 = 0$ and the right one is when $(\chi _1, \chi _2) = (0, {\tilde{\chi }}_2)$. By rows, the graphics are for initial tumor population $\approx 10 \%$, $25 \%$, $50 \%$ and $70 \%$ respectively from top to bottom. Line codes, $y_1$: blue crossed, $y_2$: red circled, $y_3$: yellow diamonded, $y_4$: purple squared, $y_5$: green 5 points stared, $y_6$: cyan 6 points stared (color figure online)

The evolution of the 6-populations with optimal therapies $\chi _i$ can be seen in Figs. 7 and 8. By looking at these results, we can say that the behavior fitting 2 parameters is analogous to the one at the 5-populations model fitting 1 parameter, which justifies the reduction from 3 to 2 in the number of therapies to fit. On the other hand, for the 1 parameter situation, it is only possible to take $\chi _2$ to ${\tilde{\chi }}_2$ although this is not recommended because it leads to the absence of the immune population, giving us similar results as the 5-populations model. To sum up, we show together the best situation for each number of parameters $\chi _i$ in Fig. 9.

## Conclusions

In this work we have proposed and studied two quasispecies ODE systems that model the behaviour of different cell states of a tumor, including or not their interaction with immune cells. We have fitted the biological transmission parameters to approximate the model results to a real situation of progressive tumor invasion. Moreover, we have analyzed the identifiability of the optimal transmission parameters using two sensibility analysis methods: *PCA* *Method* and *Eigenvalues Method*. It is concluded that two of the five transmission parameters, namely $Q_3$ and $Q_4$, are non-identifiable, which is justified fitting only the other three parameters obtaining similar results and saving significantly the computational cost. From a biological point of view, this means that during the process of progressive growing of the tumor, modifications in the fractions of change of state from extended lifespan to immortal cells ($Q_3$) and from immortal to tumor cells ($Q_4$) produce practically the same results.

Afterwards, we have introduced three different therapies, which are adjusted starting at different stages of initial tumor, to progressively reduce the tumor cells while replicative and senescent cells invade. More concretely, our study reaches the following main conclusions:

1. For the 5-populations model, if the tumor is treated at an early stage, with a small size, the best is Therapy 2 (passing the tumor cells into senescent cells, i.e., reinforcing the transition to senescence), and the more advanced the initial tumor is, the more effective Therapy 1 is (directly eliminate the tumor cells).
2. In the case of the 6-populations model, when immune cells are also taken into account, the most efficient therapy is always Therapy 3 (reinforcement of the immune cells), regardless of the initial tumor size. Again, as in the previous model, Therapy 2 is more effective in the early stages of the tumor, and the more advanced the initial tumor, the more efficient Therapy 1 is.

On the other hand, using identifiability analysis to the optimal therapy parameters, we conclude that any therapy parameter is non-identifiable by itself, which means that each therapy produces non-negligible effects acting independently. Moreover, we have considered new therapy parameters as linear combinations of the original ones, noting that the size of the variability of therapy outcomes is reduced by at least one. Indeed, there exists an eigenvector direction with very small eigenvalue, so that by perturbing the optimal combinations of therapies in this direction, the results can be practically maintained. This implies that the optimal combinations of therapies can be modified obtaining similar effects.

### Supplementary Information

Below is the link to the electronic supplementary material.

Supplementary file 1 (pdf 2529 KB)
