# Supplementary material for: Fitting parameters and therapies of ODE tumor models with senescence and immune system
Source: J Math Biol. 2023 Oct 8;87(5):67. doi: 10.1007/s00285-023-02000-9 (PMC10560657; doi:10.1007/s00285-023-02000-9)
Supplement: Supplementary file 1 — (pdf 2529 KB) [file 285_2023_2000_MOESM1_ESM.pdf]

Supplementary material

A Initial and final values

In this Supplementary Material section, we provide the information about the initial and the final state of each population (as well as the starting time t^* of therapies) for each model, with or without therapy.

A.1 Tumor Invasion

The value at the final time of each population for each model using the optimal parameter combinations (calculated for all parameters at Section 2 and only for the identifiable parameters at Subsection 4.2) are given in Table 9:

		$y_1(T)$	$y_2(T)$	$y_3(T)$	$y_4(T)$	$y_5(T)$	$y_6(T)$
**5 Param.**	**5 Pop.**	$5.2782 \cdot 10^{-8}$	$1.0798 \cdot 10^{-8}$	$9.4267 \cdot 10^{-17}$	$9.4544 \cdot 10^{-24}$	≈ 1	–
	**6 Pop.**	$3.4428 \cdot 10^{-8}$	$7.4634 \cdot 10^{-9}$	$1.2771 \cdot 10^{-20}$	$1.2586 \cdot 10^{-29}$	≈ 1	$1.5662 \cdot 10^{-18}$
**3 Param.**	**5 Pop.**	$2.5872 \cdot 10^{-8}$	$4.6065 \cdot 10^{-9}$	$1.8391 \cdot 10^{-19}$	$2.8933 \cdot 10^{-28}$	≈ 1	–
	**6 Pop.**	$6.8356 \cdot 10^{-7}$	$1.2379 \cdot 10^{-7}$	$1.0159 \cdot 10^{-17}$	$8.7597 \cdot 10^{-26}$	≈ 1	$2.6844 \cdot 10^{-17}$

Table 9: Populations at the final time for the optimal values of the transmission parameters.

A.2 Therapies

The value at the initial time t^* at which therapies were initiated and the value of each population at t^*, for both models and for each tumor initial value studied in Section 5 are listed in Table 10.

		t^*	$y0_1$	$y0_2$	$y0_3$	$y0_4$	$y0_5$	$y0_6$
$y0_5 \approx 0.1$	**5 Pop.**	6.2125	$7.5037 \cdot 10^{-1}$	$1.3374 \cdot 10^{-1}$	$6.023 \cdot 10^{-12}$	$7.8243 \cdot 10^{-21}$	$1.1588 \cdot 10^{-1}$	–
	**6 Pop.**	5.8458	$7.5671 \cdot 10^{-1}$	$1.3726 \cdot 10^{-1}$	$1.2337 \cdot 10^{-11}$	$9.1854 \cdot 10^{-20}$	$1.0602 \cdot 10^{-1}$	$2.5763 \cdot 10^{-10}$
$y0_5 \approx 0.25$	**5 Pop.**	6.341	$6.3487 \cdot 10^{-1}$	$1.1316 \cdot 10^{-1}$	$5.0959 \cdot 10^{-12}$	$6.6199 \cdot 10^{-21}$	$2.5197 \cdot 10^{-1}$	–
	**6 Pop.**	6.0208	$5.9171 \cdot 10^{-1}$	$1.0733 \cdot 10^{-1}$	$9.6469 \cdot 10^{-12}$	$7.1825 \cdot 10^{-20}$	$3.0096 \cdot 10^{-1}$	$7.8351 \cdot 10^{-11}$
$y0_5 \approx 0.5$	**5 Pop.**	6.5352	$3.6171 \cdot 10^{-1}$	$6.447 \cdot 10^{-2}$	$2.9033 \cdot 10^{-12}$	$3.7716 \cdot 10^{-21}$	$5.7382 \cdot 10^{-1}$	–
	**6 Pop.**	6.164	$3.8495 \cdot 10^{-1}$	$6.9827 \cdot 10^{-2}$	$6.276 \cdot 10^{-12}$	$4.6727 \cdot 10^{-20}$	$5.4522 \cdot 10^{-1}$	$3.4079 \cdot 10^{-11}$
$y0_5 \approx 0.7$	**5 Pop.**	6.6517	$2.1231 \cdot 10^{-1}$	$3.7841 \cdot 10^{-2}$	$1.7041 \cdot 10^{-12}$	$2.2138 \cdot 10^{-21}$	$7.4985 \cdot 10^{-1}$	–
	**6 Pop.**	6.2762	$2.3457 \cdot 10^{-1}$	$4.2549 \cdot 10^{-2}$	$3.8243 \cdot 10^{-12}$	$2.8473 \cdot 10^{-20}$	$7.2288 \cdot 10^{-1}$	$1.8259 \cdot 10^{-11}$

Table 10: Initial time and values of the populations when therapies are applied.

Also, the value at the final time of each population of each model and for each tumor initial value using the optimal combination of parameters are given in Table 11.

		$y_1(T)$	$y_2(T)$	$y_3(T)$	$y_4(T)$	$y_5(T)$	$y_6(T)$
$y0_5 \approx 0.1$	**5 Pop.**	$8.1445 \cdot 10^{-1}$	$1.5942 \cdot 10^{-1}$	$6.5373 \cdot 10^{-12}$	$8.4925 \cdot 10^{-21}$	$2.6126 \cdot 10^{-2}$	–
	**6 Pop.**	$8.0183 \cdot 10^{-1}$	$1.5692 \cdot 10^{-1}$	$1.3073 \cdot 10^{-11}$	$9.733 \cdot 10^{-20}$	$2.3075 \cdot 10^{-2}$	$1.8168 \cdot 10^{-2}$
$y0_5 \approx 0.25$	**5 Pop.**	$8.0024 \cdot 10^{-1}$	$1.563 \cdot 10^{-1}$	$6.4232 \cdot 10^{-12}$	$8.3443 \cdot 10^{-21}$	$4.3461 \cdot 10^{-2}$	–
	**6 Pop.**	$7.7213 \cdot 10^{-1}$	$1.5108 \cdot 10^{-1}$	$1.2588 \cdot 10^{-11}$	$9.3725 \cdot 10^{-20}$	$4.3485 \cdot 10^{-2}$	$3.3303 \cdot 10^{-2}$
$y0_5 \approx 0.5$	**5 Pop.**	$7.6582 \cdot 10^{-1}$	$1.5269 \cdot 10^{-1}$	$6.147 \cdot 10^{-12}$	$7.9854 \cdot 10^{-21}$	$8.1484 \cdot 10^{-2}$	–
	**6 Pop.**	$7.5644 \cdot 10^{-1}$	$1.4454 \cdot 10^{-1}$	$1.2333 \cdot 10^{-11}$	$9.1821 \cdot 10^{-20}$	$3.7762 \cdot 10^{-2}$	$6.1258 \cdot 10^{-2}$
$y0_5 \approx 0.7$	**5 Pop.**	$7.5441 \cdot 10^{-1}$	$1.4975 \cdot 10^{-1}$	$6.0554 \cdot 10^{-12}$	$7.8664 \cdot 10^{-21}$	$9.5837 \cdot 10^{-2}$	–
	**6 Pop.**	$7.9002 \cdot 10^{-1}$	$1.4497 \cdot 10^{-1}$	$1.288 \cdot 10^{-11}$	$9.5897 \cdot 10^{-20}$	$4.5316 \cdot 10^{-2}$	$1.9689 \cdot 10^{-2}$

Table 11: Final values of the populations for the optimal therapy parameters.

A.3 New therapies parameters

The models associated with these data are discussed in Section 7. The initial value of each population, as well as t^*, for both models and for each tumor initial value are the same as those in Supplementary Material A.2.

A.3.1 5-populations model

The final value of each population for each tumor initial value using the different parameter combinations are given in Table 12.

		$y_1(T)$	$y_2(T)$	$y_3(T)$	$y_4(T)$	$y_5(T)$
$y_{0_5} \approx 0.1$	(χ_1, χ_2)	$8.1446 \cdot 10^{-1}$	$1.5944 \cdot 10^{-1}$	$6.5374 \cdot 10^{-12}$	$8.4926 \cdot 10^{-21}$	$2.6097 \cdot 10^{-2}$
	$(0, \chi_2)$	$8.1415 \cdot 10^{-1}$	$1.574 \cdot 10^{-1}$	$6.5348 \cdot 10^{-12}$	$8.4894 \cdot 10^{-21}$	$2.8449 \cdot 10^{-2}$
	$(\tilde{\chi}_1, \chi_2)$	$8.1435 \cdot 10^{-1}$	$1.5565 \cdot 10^{-1}$	$6.5364 \cdot 10^{-12}$	$8.4914 \cdot 10^{-21}$	$3 \cdot 10^{-2}$
$y_{0_5} \approx 0.25$	(χ_1, χ_2)	$8.0024 \cdot 10^{-1}$	$1.563 \cdot 10^{-1}$	$6.4232 \cdot 10^{-12}$	$8.3442 \cdot 10^{-21}$	$4.3461 \cdot 10^{-2}$
	$(0, \chi_2)$	$8.0081 \cdot 10^{-1}$	$1.5719 \cdot 10^{-1}$	$6.4278 \cdot 10^{-12}$	$8.3502 \cdot 10^{-21}$	$4.2003 \cdot 10^{-2}$
	$(\tilde{\chi}_1, \chi_2)$	$8.0299 \cdot 10^{-1}$	$1.5941 \cdot 10^{-1}$	$6.4453 \cdot 10^{-12}$	$8.373 \cdot 10^{-21}$	$3.7601 \cdot 10^{-2}$
$y_{0_5} \approx 0.5$	(χ_1, χ_2)	$7.6582 \cdot 10^{-1}$	$1.5269 \cdot 10^{-1}$	$6.147 \cdot 10^{-12}$	$7.9854 \cdot 10^{-21}$	$8.1486 \cdot 10^{-2}$
	$(0, \chi_2)$	$7.7296 \cdot 10^{-1}$	$1.577 \cdot 10^{-1}$	$6.2042 \cdot 10^{-12}$	$8.0598 \cdot 10^{-21}$	$6.9345 \cdot 10^{-2}$
	$(\tilde{\chi}_1, \chi_2)$	$7.9055 \cdot 10^{-1}$	$1.6393 \cdot 10^{-1}$	$6.3455 \cdot 10^{-12}$	$8.2433 \cdot 10^{-21}$	$4.5521 \cdot 10^{-2}$
$y_{0_5} \approx 0.7$	(χ_1, χ_2)	$7.5441 \cdot 10^{-1}$	$1.4975 \cdot 10^{-1}$	$6.0554 \cdot 10^{-12}$	$7.8664 \cdot 10^{-21}$	$9.5836 \cdot 10^{-2}$
	$(0, \chi_2)$	$7.6684 \cdot 10^{-1}$	$1.5692 \cdot 10^{-1}$	$6.1552 \cdot 10^{-12}$	$7.9961 \cdot 10^{-21}$	$7.6237 \cdot 10^{-2}$
	$(\tilde{\chi}_1, \chi_2)$	$7.9357 \cdot 10^{-1}$	$1.6423 \cdot 10^{-1}$	$6.3697 \cdot 10^{-12}$	$8.2747 \cdot 10^{-21}$	$4.2201 \cdot 10^{-2}$

Table 12: Final values of the populations for the optimal values of the new therapy parameters for the 5-populations model.

A.3.2 6-populations model

The results are similar to the 5-populations model.

B Sensitivity analysis

We study the derivatives of the populations with respect to the parameters.

B.1 Tumor invasion

Figures 10 and 11 show the derivatives with respect to the parameters $\{Q_1, Q_3, Q_4, \alpha, \beta\}$, which lead us to the tumor invasion at their optimal values, for the 5 and 6-populations models, respectively (it is important to note not only the shape but also the scale of the graphics).

Looking at Figures 10 and 11, we can deduce that the derivatives with respect to Q_4 and Q_3 are rather small (showing the little identifiability of parameters Q_4 and Q_3) whereas derivatives with respect to α and β are dominant.

We can appreciate that all the derivatives have a similar shape, presenting the greatest variation at the beginning of the tumor invasion and showing that an increase of any of these parameters benefits the replicative population (and to a lesser extent the senescent

		$y_1(T)$	$y_2(T)$	$y_3(T)$	$y_4(T)$	$y_5(T)$	$y_6(T)$
$y_{0_5} \approx 0.1$	(χ_1,χ_2,χ_3)	$8.0183 \cdot 10^{-1}$	$1.5692 \cdot 10^{-1}$	$1.3072 \cdot 10^{-11}$	$9.733 \cdot 10^{-20}$	$2.308 \cdot 10^{-2}$	$1.8172 \cdot 10^{-2}$
	$(0,\chi_2,\chi_3)$	$8.0157 \cdot 10^{-1}$	$1.5445 \cdot 10^{-1}$	$1.3068 \cdot 10^{-11}$	$9.7298 \cdot 10^{-20}$	$2.5713 \cdot 10^{-2}$	$1.827 \cdot 10^{-2}$
	$(\tilde{\chi}_1,\chi_2,\chi_3)$	$8.1446 \cdot 10^{-1}$	$1.5845 \cdot 10^{-1}$	$1.3278 \cdot 10^{-11}$	$9.8863 \cdot 10^{-20}$	$2.709 \cdot 10^{-2}$	$4.2482 \cdot 10^{-24}$
	$(0,0,\chi_3)$	–	–	–	–	–	–
	$(0,\tilde{\chi}_2,\chi_3)$	$8.1454 \cdot 10^{-1}$	$1.5812 \cdot 10^{-1}$	$1.328 \cdot 10^{-11}$	$9.8872 \cdot 10^{-20}$	$2.735 \cdot 10^{-2}$	$3.4815 \cdot 10^{-20}$
	$(\tilde{\chi}_1,0,\chi_3)$	–	–	–	–	–	–
	$(\tilde{\chi}_1,\tilde{\chi}_2,\chi_3)$	$8.1452 \cdot 10^{-1}$	$1.5782 \cdot 10^{-1}$	$1.3279 \cdot 10^{-11}$	$9.887 \cdot 10^{-20}$	$2.7658 \cdot 10^{-2}$	$3.3651 \cdot 10^{-20}$
$y_{0_5} \approx 0.25$	(χ_1,χ_2,χ_3)	$7.7211 \cdot 10^{-1}$	$1.5107 \cdot 10^{-1}$	$1.2588 \cdot 10^{-11}$	$9.3723 \cdot 10^{-20}$	$4.3474 \cdot 10^{-2}$	$3.334 \cdot 10^{-2}$
	$(0,\chi_2,\chi_3)$	$7.7167 \cdot 10^{-1}$	$1.5041 \cdot 10^{-1}$	$1.2581 \cdot 10^{-11}$	$9.3669 \cdot 10^{-20}$	$4.4663 \cdot 10^{-2}$	$3.3261 \cdot 10^{-2}$
	$(\tilde{\chi}_1,\chi_2,\chi_3)$	$7.7642 \cdot 10^{-1}$	$1.5498 \cdot 10^{-1}$	$1.2658 \cdot 10^{-11}$	$9.4246 \cdot 10^{-20}$	$3.3516 \cdot 10^{-2}$	$3.5077 \cdot 10^{-2}$
	$(0,0,\chi_3)$	–	–	–	–	–	–
	$(0,\tilde{\chi}_2,\chi_3)$	$7.928 \cdot 10^{-1}$	$1.571 \cdot 10^{-1}$	$1.2925 \cdot 10^{-11}$	$9.6234 \cdot 10^{-20}$	$5.0096 \cdot 10^{-2}$	$1.6707 \cdot 10^{-15}$
	$(\tilde{\chi}_1,0,\chi_3)$	–	–	–	–	–	–
	$(\tilde{\chi}_1,\tilde{\chi}_2,\chi_3)$	$7.9947 \cdot 10^{-1}$	$1.6239 \cdot 10^{-1}$	$1.3034 \cdot 10^{-11}$	$9.7043 \cdot 10^{-20}$	$3.8139 \cdot 10^{-2}$	$1.7705 \cdot 10^{-15}$
$y_{0_5} \approx 0.5$	(χ_1,χ_2,χ_3)	$7.5644 \cdot 10^{-1}$	$1.4454 \cdot 10^{-1}$	$1.2333 \cdot 10^{-11}$	$9.1821 \cdot 10^{-20}$	$3.7762 \cdot 10^{-2}$	$6.1259 \cdot 10^{-2}$
	$(0,\chi_2,\chi_3)$	$7.5678 \cdot 10^{-1}$	$1.4466 \cdot 10^{-1}$	$1.2338 \cdot 10^{-11}$	$9.1862 \cdot 10^{-20}$	$3.7511 \cdot 10^{-2}$	$6.1047 \cdot 10^{-2}$
	$(\tilde{\chi}_1,\chi_2,\chi_3)$	$7.775 \cdot 10^{-1}$	$1.5015 \cdot 10^{-1}$	$1.2676 \cdot 10^{-11}$	$9.4376 \cdot 10^{-20}$	$1.945 \cdot 10^{-2}$	$5.2907 \cdot 10^{-2}$
	$(0,0,\chi_3)$	–	–	–	–	–	–
	$(0,\tilde{\chi}_2,\chi_3)$	$7.6803 \cdot 10^{-1}$	$1.5623 \cdot 10^{-1}$	$1.2521 \cdot 10^{-11}$	$9.3227 \cdot 10^{-20}$	$7.5744 \cdot 10^{-2}$	$4.2049 \cdot 10^{-10}$
	$(\tilde{\chi}_1,0,\chi_3)$	–	–	–	–	–	–
	$(\tilde{\chi}_1,\tilde{\chi}_2,\chi_3)$	$7.898 \cdot 10^{-1}$	$1.657 \cdot 10^{-1}$	$1.2876 \cdot 10^{-11}$	$9.587 \cdot 10^{-20}$	$4.4504 \cdot 10^{-2}$	$1.6573 \cdot 10^{-10}$
$y_{0_5} \approx 0.7$	(χ_1,χ_2,χ_3)	$7.8948 \cdot 10^{-1}$	$1.4489 \cdot 10^{-1}$	$1.2871 \cdot 10^{-11}$	$9.5831 \cdot 10^{-20}$	$4.5838 \cdot 10^{-2}$	$1.9787 \cdot 10^{-2}$
	$(0,\chi_2,\chi_3)$	$8.0533 \cdot 10^{-1}$	$1.5175 \cdot 10^{-1}$	$1.313 \cdot 10^{-11}$	$9.7755 \cdot 10^{-20}$	$2.9349 \cdot 10^{-2}$	$1.3572 \cdot 10^{-2}$
	$(\tilde{\chi}_1,\chi_2,\chi_3)$	$8.203 \cdot 10^{-1}$	$1.5228 \cdot 10^{-1}$	$1.3374 \cdot 10^{-11}$	$9.9572 \cdot 10^{-20}$	$6.2125 \cdot 10^{-3}$	$2.121 \cdot 10^{-2}$
	$(0,0,\chi_3)$	–	–	–	–	–	–
	$(0,\tilde{\chi}_2,\chi_3)$	$7.5718 \cdot 10^{-1}$	$1.5487 \cdot 10^{-1}$	$1.2345 \cdot 10^{-11}$	$9.1911 \cdot 10^{-20}$	$8.7941 \cdot 10^{-2}$	$7.642 \cdot 10^{-6}$
	$(\tilde{\chi}_1,0,\chi_3)$	–	–	–	–	–	–
	$(\tilde{\chi}_1,\tilde{\chi}_2,\chi_3)$	$7.9518 \cdot 10^{-1}$	$1.6655 \cdot 10^{-1}$	$1.2964 \cdot 10^{-11}$	$9.6523 \cdot 10^{-20}$	$3.8269 \cdot 10^{-2}$	$9.0879 \cdot 10^{-8}$

Table 13: Final values of the populations for the optimal values of the new therapy parameters for the 6-populations model.

population) whereas it is detrimental for the tumor population. It also shows that there is a transient stability before the tumor invasion where increasing the parameters increases the replicative population while the senescent population decreases but to a lesser extent than the other (also only seen in the Q_1 graphic).

B.2 Therapies

We show the derivatives with respect to therapy parameters for each model.

B.2.1 5-populations model

The derivatives of the populations with respect to each therapy parameter for this model at each tumor initial value can be seen in Figure 12.

Observe that the scale for both parameters increases with the tumor initial value and the derivatives with respect to parameter κ_1 have a greater scale than with respect to the parameter κ_2 in all cases.

- Increasing the parameter κ_1 benefits the replicative population while it is detrimental mainly for the tumor and slightly to the senescent one (which is even less the greater is the tumor initial value), increasing to its absolute maximum value quickly and decreasing gradually over time in all cases.

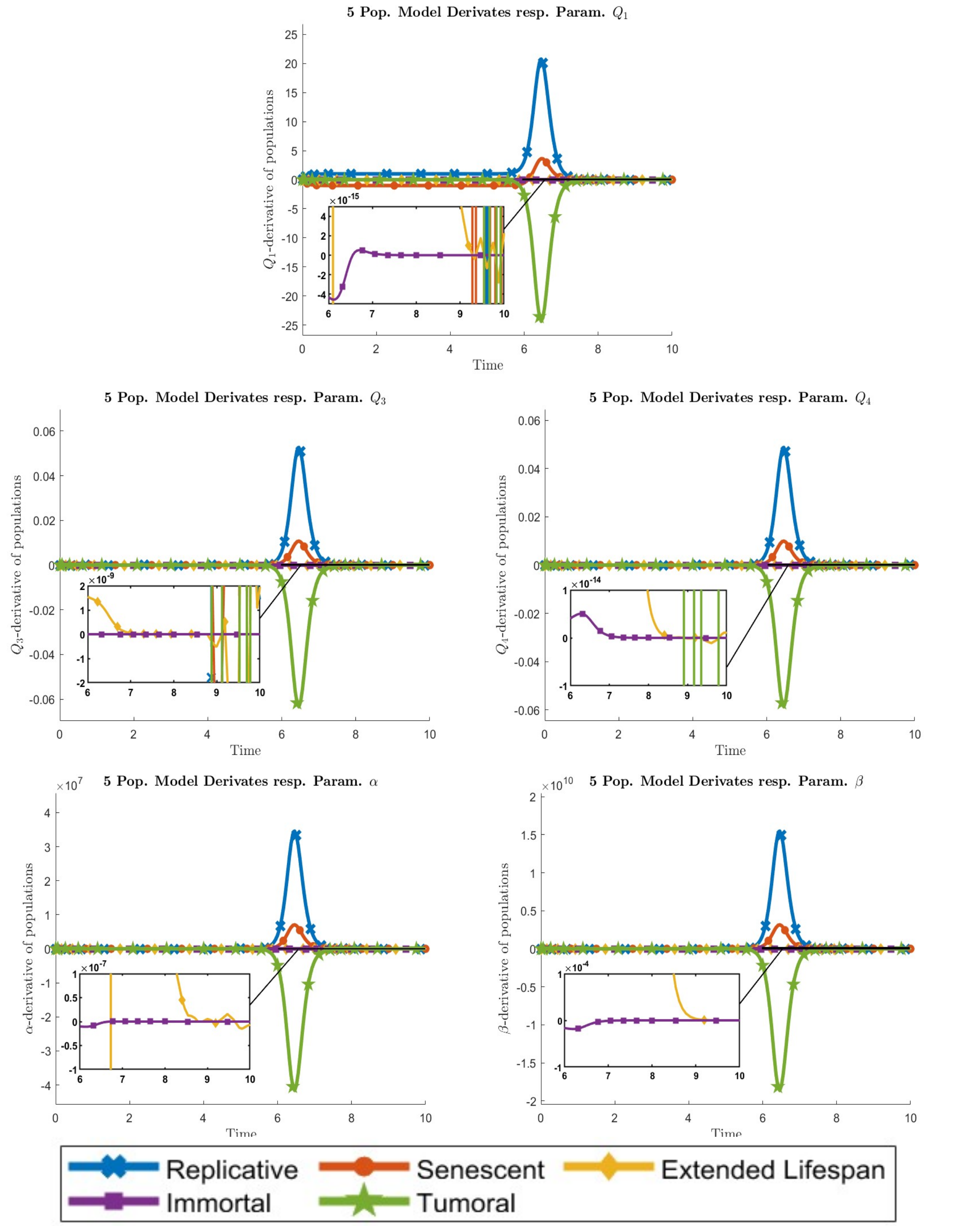

Figure 10: Dynamical behavior of the derivatives of the five cell types for each transmission parameter. The upper graphic is for Q_1, the middle-left one for Q_3, the middle-right one for Q_4, the bottom-left one for α and the bottom-right one for β. Line codes, y_1: blue crossed, y_2: red circled, y_3: yellow diamonded, y_4: purple squared, y_5: green 5 points stared.

- Increasing the parameter κ_2 causes a similar effect on the populations as κ_1 except for the senescent cells, which initially feels beneficial (which remains in time longer the greater the tumor initial value is) and, at a certain time, it starts to decrease, reaching negative values but in lesser extent the greater is the tumor initial value.

B.2.2 6-populations model

The derivatives of the populations with respect to each therapy parameter at each tumor initial value can be seen in Figure 13.

We can deduce that the scale for all the parameters increases with respect to the initial tumor (except for κ_2, whose scale decrease for initial tumor $\approx 70\%$).

- Increasing the parameter κ_1 causes the following effects:
  - For the replicative population, the derivative increases all the time except at the end, when the benefit becomes pronounced. The higher the initial tumor, the earlier the change in trend occurs, and it also occurs after the benefits start to wane and disappear.
  - For the senescent population, it causes different behaviors over time (some of which can only be observed when the initial tumor is large enough). Initially, it causes a small decrease but will eventually disappear and then a slightly larger increase will start but will also go away eventually.
  - For the tumor population, the behavior is similar to that of the senescent population, but always remaining below, highlighting the case of low initial tumor where its detrimental effect is considerable, contrasting with the benefit for the replicative population, except for the last moments, when the immune population replaces the tumor.
  - For the immune population, it causes a detrimental effect, but for low initial tumor its abrupt change occurs only at the end and the bigger the initial tumor is the earlier this abrupt change occurs, which reaches its limit and begins to decrease (precipitously too) until it disappears.
- Increasing the parameter κ_2 has, in form, practically the same effects as the previous parameter κ_1.
- Increasing the parameter κ_3 has no effect on the populations at the beginning, but in the end it has a precipitous benefit for the immune population and an almost analogous effect for the replicative but this time detrimental. It is also detrimental, to a lesser extent, for both senescent and tumor populations. Moreover, the higher the initial tumor, the earlier this strong variation occurs, reaching its limits and beginning to decrease until it disappears, except for the tumor population that remains a bit and the replicative one, which not only disappears its harmful effect but also begins to be slightly beneficial. The increase of the initial tumor also shortens the difference between the detrimental effect of replicative and tumor populations.

C Eigenvectors and Eigenvalues

We provide the eigenvectors and eigenvalues for each identifiability method and for each model, with or without therapy.

C.1 Tumor Invasion

C.1.1 5-populations model

- ***PCA* Method**
  - y_1 population:

$$\underline{\gamma}_1^1 = (6.8739 \cdot 10^{-8}, -1.5435 \cdot 10^{-1}, \mathbf{9.8802 \cdot 10^{-1}}, -6.9106 \cdot 10^{-10}, -3.8716 \cdot 10^{-13})^t$$

$$\underline{\gamma}_2^1 = (2.8952 \cdot 10^{-7}, \mathbf{-9.8802 \cdot 10^{-1}}, -1.5435 \cdot 10^{-1}, 5.6699 \cdot 10^{-9}, -4.3503 \cdot 10^{-12})^t$$

$$\underline{\gamma}_3^1 = (\mathbf{\approx -1}, -2.9665 \cdot 10^{-7}, 2.3229 \cdot 10^{-8}, 5.1695 \cdot 10^{-4}, -6.3025 \cdot 10^{-7})^t$$

$$\underline{\gamma}_4^1 = (-5.1695 \cdot 10^{-4}, -5.6487 \cdot 10^{-9}, -1.5459 \cdot 10^{-9}, \mathbf{\approx -1}, 1.2209 \cdot 10^{-3})^t$$

$$\underline{\gamma}_5^1 = (9.0795 \cdot 10^{-10}, 2.3518 \cdot 10^{-12}, 1.6132 \cdot 10^{-12}, 1.2209 \cdot 10^{-3}, \mathbf{\approx 1})^t$$

$|\lambda_1^1| = 2.6009 \cdot 10^{-16}$, $|\lambda_2^1| = 1.6151 \cdot 10^{-15}$, $|\lambda_3^1| = 7.1578 \cdot 10^{-3}$, $|\lambda_4^1| = 4.5353 \cdot 10^{3}$, $|\lambda_5^1| = 6.4713 \cdot 10^{16}$.

  - y_2 population:

$$\underline{\gamma}_1^2 = (1.0003 \cdot 10^{-8}, -2.4595 \cdot 10^{-2}, \mathbf{-9.9970 \cdot 10^{-1}}, 1.7198 \cdot 10^{-9}, -4.2925 \cdot 10^{-13})^t$$

$$\underline{\gamma}_2^2 = (2.3209 \cdot 10^{-7}, \mathbf{-9.9970 \cdot 10^{-1}}, 2.4595 \cdot 10^{-2}, 6.3775 \cdot 10^{-9}, -5.475 \cdot 10^{-12})^t$$

$$\underline{\gamma}_3^2 = (\mathbf{-9.9999 \cdot 10^{-1}}, -2.3229 \cdot 10^{-7}, -4.2980 \cdot 10^{-9}, -3.8317 \cdot 10^{-3}, 4.6787 \cdot 10^{-6})^t$$

$$\underline{\gamma}_4^2 = (3.8317 \cdot 10^{-3}, -5.5279 \cdot 10^{-9}, -1.546 \cdot 10^{-9}, \mathbf{-9.9999 \cdot 10^{-1}}, 1.2209 \cdot 10^{-3})^t$$

$$\underline{\gamma}_5^2 = (4.5463 \cdot 10^{-10}, 2.3519 \cdot 10^{-12}, 1.6132 \cdot 10^{-12}, 1.2209 \cdot 10^{-3}, \mathbf{1.0000 \cdot 10^{0}})^t$$

$|\lambda_1^2| = 1.2482 \cdot 10^{-17}$, $|\lambda_2^2| = 1.3996 \cdot 10^{-15}$, $|\lambda_3^2| = 7.1469 \cdot 10^{-3}$, $|\lambda_4^2| = 1.8986 \cdot 10^{2}$, $|\lambda_5^2| = 2.7093 \cdot 10^{15}$.

  - y_3 population:

$$\underline{\gamma}_1^3 = (-7.0618 \cdot 10^{-6}, 4.1839 \cdot 10^{-9}, \mathbf{\approx -1}, 3.6654 \cdot 10^{-13}, 1.6186 \cdot 10^{-12})^t$$

$$\underline{\gamma}_2^3 = (\mathbf{\approx 1}, -2.7201 \cdot 10^{-3}, -7.0618 \cdot 10^{-6}, -5.2782 \cdot 10^{-8}, -7.7424 \cdot 10^{-10})^t$$

$$\underline{\gamma}_3^3 = (2.7201 \cdot 10^{-3}, \mathbf{\approx 1}, -1.5025 \cdot 10^{-8}, 4.1507 \cdot 10^{-8}, 7.3782 \cdot 10^{-10})^t$$

$$\underline{\gamma}_4^3 = (5.2673 \cdot 10^{-8}, -4.1654 \cdot 10^{-8}, 4.1416 \cdot 10^{-15}, \mathbf{9.9998 \cdot 10^{-1}}, 6.0223 \cdot 10^{-3})^t$$

$$\underline{\gamma}_5^3 = (4.5503 \cdot 10^{-10}, -4.8908 \cdot 10^{-10}, 1.6131 \cdot 10^{-12}, -6.0223 \cdot 10^{-3}, \mathbf{9.9998 \cdot 10^{-1}})^t$$

$|\lambda_1^3| = 3.565{\cdot}10^{-33}$, $|\lambda_2^3| = 5.1562{\cdot}10^{-22}$, $|\lambda_3^3| = 2.1262{\cdot}10^{-18}$, $|\lambda_4^3| = 2.7163{\cdot}10^{-4}$, $|\lambda_5^3| = 2.0659{\cdot}10^{-1}$.

– y_4 population:

$$\underline{\gamma}_1^4 = (2.4687 \cdot 10^{-4}, \ \approx \mathbf{-1}, \ 4.8558 \cdot 10^{-4}, \ 2.2229 \cdot 10^{-9}, \ -6.0372 \cdot 10^{-13})^t$$

$$\underline{\gamma}_2^4 = (6.9349 \cdot 10^{-1}, \ 5.2105 \cdot 10^{-4}, \ \mathbf{7.2047 \cdot 10^{-1}}, \ -7.3435 \cdot 10^{-7}, \ 2.6275 \cdot 10^{-10})^t$$

$$\underline{\gamma}_3^4 = (\mathbf{7.2047 \cdot 10^{-1}}, \ -1.5888 \cdot 10^{-4}, \ -6.9349 \cdot 10^{-1}, \ -1.6211 \cdot 10^{-7}, \ -1.8495 \cdot 10^{-10})^t$$

$$\underline{\gamma}_4^4 = (-6.2606 \cdot 10^{-7}, \ -2.5798 \cdot 10^{-9}, \ -4.1665 \cdot 10^{-7}, \ \approx \mathbf{-1}, \ 3.6302 \cdot 10^{-4})^t$$

$$\underline{\gamma}_5^4 = (1.783 \cdot 10^{-10}, \ 1.6649 \cdot 10^{-13}, \ -1.6631 \cdot 10^{-10}, \ 3.6302 \cdot 10^{-4}, \ \approx \mathbf{1})^t$$

$|\lambda_1^4| = 1.9977{\cdot}10^{-41}$, $|\lambda_2^4| = 3.6972{\cdot}10^{-39}$, $|\lambda_3^4| = 1.4388{\cdot}10^{-34}$, $|\lambda_4^4| = 5.1416{\cdot}10^{-22}$, $|\lambda_5^4| = 8.1341{\cdot}10^{-13}$.

– y_5 population:

$$\underline{\gamma}_1^5 = (2.5336 \cdot 10^{-5}, \ \mathbf{9.9154 \cdot 10^{-1}}, \ -1.2984 \cdot 10^{-1}, \ 2.3661 \cdot 10^{-10}, \ -2.4324 \cdot 10^{-12})^t$$

$$\underline{\gamma}_2^5 = (-2.9983 \cdot 10^{-6}, \ -1.2984 \cdot 10^{-1}, \ \mathbf{-9.9154 \cdot 10^{-1}}, \ 1.5991 \cdot 10^{-9}, \ -4.5097 \cdot 10^{-14})^t$$

$$\underline{\gamma}_3^5 = (\approx \mathbf{1}, \ -2.5511 \cdot 10^{-5}, \ 3.167 \cdot 10^{-7}, \ 2.2176 \cdot 10^{-4}, \ -2.7159 \cdot 10^{-7})^t$$

$$\underline{\gamma}_4^5 = (2.2176 \cdot 10^{-4}, \ -5.6304 \cdot 10^{-9}, \ -1.5461 \cdot 10^{-9}, \ \approx \mathbf{-1}, \ 1.2209 \cdot 10^{-3})^t$$

$$\underline{\gamma}_5^5 = (8.3095 \cdot 10^{-10}, \ 2.3518 \cdot 10^{-12}, \ 1.6132 \cdot 10^{-12}, \ 1.2209 \cdot 10^{-3}, \ \approx \mathbf{1})^t$$

$|\lambda_1^5| = 1.2185{\cdot}10^{-16}$, $|\lambda_2^5| = 4.351{\cdot}10^{-16}$, $|\lambda_3^5| = 3.9904{\cdot}10^{-8}$, $|\lambda_4^5| = 6.5797{\cdot}10^{3}$, $|\lambda_5^5| = 9.3904{\cdot}10^{16}$.

Therefore, the biological parameters ordered from least identifiable to most identifiable are:
$Q_4, Q_3, Q_1, \alpha, \beta$ for y_1 and y_2,
$Q_3, Q_4, Q_1, \alpha, \beta$ for y_4 and y_5,
$Q_4, Q_1, Q_3, \alpha, \beta$ for y_3.

- **Eigenvalues Method**

$$\underline{\gamma}_1 = (-4.1985 \cdot 10^{-7}, \ -1.5996 \cdot 10^{-1}, \ \mathbf{9.8712 \cdot 10^{-1}}, \ -6.4778 \cdot 10^{-10}, \ -4.2336 \cdot 10^{-13})^t$$

$$\underline{\gamma}_2 = (-1.688 \cdot 10^{-7}, \ \mathbf{-9.8712 \cdot 10^{-1}}, \ -1.5996 \cdot 10^{-1}, \ 1.1342 \cdot 10^{-9}, \ 1.1903 \cdot 10^{-12})^t$$

$$\underline{\gamma}_3 = (\approx \mathbf{-1}, \ 2.3378 \cdot 10^{-7}, \ -3.8744 \cdot 10^{-7}, \ -4.3764 \cdot 10^{-7}, \ 1.3916 \cdot 10^{-9})^t$$

$$\underline{\gamma}_4 = (4.3764 \cdot 10^{-7}, \ -1.0161 \cdot 10^{-9}, \ -8.207 \cdot 10^{-10}, \ \approx \mathbf{-1}, \ 1.2201 \cdot 10^{-3})^t$$

$$\underline{\gamma}_5 = (8.5766 \cdot 10^{-10}, \ 2.3466 \cdot 10^{-12}, \ 1.6102 \cdot 10^{-12}, \ 1.2201 \cdot 10^{-3}, \ \approx \mathbf{1})^t$$

$|\lambda_1| = 1.0396{\cdot}10^{-11}$, $|\lambda_2| = 8.385{\cdot}10^{-11}$, $|\lambda_3| = 3.9762{\cdot}10^{-1}$, $|\lambda_4| = 3.2842{\cdot}10^{7}$, $|\lambda_5| = 3.3321{\cdot}10^{19}$.

As a consequence, the biological parameters, ordered from least identifiable to most identifiable are:

Q_4, Q_3, Q_1, α, β.

C.1.2 6-populations model

- ***PCA*** **Method**

  – y_1 population:

$$\underline{\gamma}_1^1 = (-1.6513 \cdot 10^{-8}, \quad 7.9362 \cdot 10^{-2}, \quad \mathbf{-9.9685 \cdot 10^{-1}}, \quad -1.0002 \cdot 10^{-15}, \quad -9.812 \cdot 10^{-12})^t$$

$$\underline{\gamma}_2^1 = (6.3223 \cdot 10^{-8}, \quad \mathbf{-9.9685 \cdot 10^{-1}}, \quad -7.9362 \cdot 10^{-2}, \quad -1.8352 \cdot 10^{-13}, \quad 1.159 \cdot 10^{-9})^t$$

$$\underline{\gamma}_3^1 = (\mathbf{\approx 1}, \quad 6.4334 \cdot 10^{-8}, \quad -1.1443 \cdot 10^{-8}, \quad -1.1152 \cdot 10^{-7}, \quad 5.2496 \cdot 10^{-4})^t$$

$$\underline{\gamma}_4^1 = (-5.2496 \cdot 10^{-4}, \quad 1.1223 \cdot 10^{-9}, \quad 8.8206 \cdot 10^{-11}, \quad -2.1215 \cdot 10^{-4}, \quad \mathbf{\approx 1})^t$$

$$\underline{\gamma}_5^1 = (1.4857 \cdot 10^{-10}, \quad 6.2421 \cdot 10^{-14}, \quad 1.8755 \cdot 10^{-15}, \quad \mathbf{\approx 1}, \quad 2.1215 \cdot 10^{-4})^t$$

$|\lambda_1^1| = 1.1945{\cdot}10^{-17}$, $|\lambda_2^1| = 2.2712{\cdot}10^{-16}$, $|\lambda_3^1| = 7.1694{\cdot}10^{-3}$, $|\lambda_4^1| = 5.632{\cdot}10^{3}$, $|\lambda_5^1| = 2.2734{\cdot}10^{18}$.

  – y_2 population:

$$\underline{\gamma}_1^2 = (-1.6948 \cdot 10^{-8}, \quad \mathbf{9.8498 \cdot 10^{-1}}, \quad 1.7269 \cdot 10^{-1}, \quad 2.4703 \cdot 10^{-13}, \quad -1.4557 \cdot 10^{-9})^t$$

$$\underline{\gamma}_2^2 = (-9.5153 \cdot 10^{-10}, \quad -1.7269 \cdot 10^{-1}, \quad \mathbf{9.8498 \cdot 10^{-1}}, \quad -2.9913 \cdot 10^{-14}, \quad 1.831 \cdot 10^{-10})^t$$

$$\underline{\gamma}_3^2 = (\mathbf{9.9999 \cdot 10^{-1}}, \quad 1.6524 \cdot 10^{-8}, \quad 3.8637 \cdot 10^{-9}, \quad 7.603 \cdot 10^{-7}, \quad -3.5843 \cdot 10^{-3})^t$$

$$\underline{\gamma}_4^2 = (3.5843 \cdot 10^{-3}, \quad 1.5247 \cdot 10^{-9}, \quad 8.4889 \cdot 10^{-11}, \quad -2.1215 \cdot 10^{-4}, \quad \mathbf{9.9999 \cdot 10^{-1}})^t$$

$$\underline{\gamma}_5^2 = (1.2764 \cdot 10^{-10}, \quad 6.2417 \cdot 10^{-14}, \quad 1.8755 \cdot 10^{-15}, \quad \mathbf{\approx 1}, \quad 2.1215 \cdot 10^{-4})^t$$

$|\lambda_1^2| = 1.1623{\cdot}10^{-16}$, $|\lambda_2^2| = 1.4734{\cdot}10^{-16}$, $|\lambda_3^2| = 7.0521{\cdot}10^{-3}$, $|\lambda_4^2| = 2.56{\cdot}10^{2}$, $|\lambda_5^2| = 1.0690{\cdot}10^{17}$.

  – y_3 population:

$$\underline{\gamma}_1^3 = (-3.5037 \cdot 10^{-7}, \quad -1.2167 \cdot 10^{-12}, \quad \mathbf{\approx 1}, \quad 3.1945 \cdot 10^{-18}, \quad -8.6094 \cdot 10^{-12})^t$$

$$\underline{\gamma}_2^3 = (\mathbf{\approx -1}, \quad 2.0676 \cdot 10^{-7}, \quad -3.5037 \cdot 10^{-7}, \quad 9.8805 \cdot 10^{-12}, \quad 6.375 \cdot 10^{-7})^t$$

$$\underline{\gamma}_3^3 = (2.0747 \cdot 10^{-7}, \quad \mathbf{\approx 1}, \quad 1.2991 \cdot 10^{-12}, \quad 1.7039 \cdot 10^{-8}, \quad 1.1231 \cdot 10^{-3})^t$$

$$\underline{\gamma}_4^3 = (-6.3727 \cdot 10^{-7}, \quad 1.1231 \cdot 10^{-3}, \quad -8.8313 \cdot 10^{-12}, \quad -1.6273 \cdot 10^{-7}, \quad \mathbf{\approx -1})^t$$

$$\underline{\gamma}_5^3 = (-9.7733 \cdot 10^{-12}, \quad 1.6856 \cdot 10^{-8}, \quad 1.1919 \cdot 10^{-18}, \quad \mathbf{\approx -1}, \quad 1.6275 \cdot 10^{-7})^t$$

$|\lambda_1^3| = 7.6857{\cdot}10^{-33}$, $|\lambda_2^3| = 1.8976{\cdot}10^{-29}$, $|\lambda_3^3| = 1.344{\cdot}10^{-18}$, $|\lambda_4^3| = 1.4093{\cdot}10^{-14}$, $|\lambda_5^3| = 3.0865{\cdot}10^{-4}$.

– y_4 population:

$$\underline{\gamma}_1^4 = (\boldsymbol{\approx -1},\ 3.4458 \cdot 10^{-6},\ 2.0092 \cdot 10^{-11},\ 1.3843 \cdot 10^{-11},\ -1.1902 \cdot 10^{-13})^t$$

$$\underline{\gamma}_2^4 = (-3.4458 \cdot 10^{-6},\ \boldsymbol{\approx -1},\ -2.8046 \cdot 10^{-7},\ -1.4634 \cdot 10^{-8},\ 4.5035 \cdot 10^{-8})^t$$

$$\underline{\gamma}_3^4 = (-2.0196 \cdot 10^{-11},\ 2.8101 \cdot 10^{-7},\ \boldsymbol{-9.9658 \cdot 10^{-1}},\ -8.2376 \cdot 10^{-2},\ 6.7888 \cdot 10^{-3})^t$$

$$\underline{\gamma}_4^4 = (1.215 \cdot 10^{-11},\ 6.5583 \cdot 10^{-9},\ -8.2600 \cdot 10^{-2},\ \boldsymbol{9.9555 \cdot 10^{-1}},\ -4.5276 \cdot 10^{-2})^t$$

$$\underline{\gamma}_5^4 = (7.2412 \cdot 10^{-13},\ 4.3469 \cdot 10^{-8},\ 3.0289 \cdot 10^{-3},\ 4.5682 \cdot 10^{-2},\ \boldsymbol{9.9895 \cdot 10^{-1}})^t$$

$|\lambda_1^4| = 3.3786{\cdot}10^{-45}$, $|\lambda_2^4| = 7.8774{\cdot}10^{-37}$, $|\lambda_3^4| = 4.6901{\cdot}10^{-24}$, $|\lambda_4^4| = 2.835{\cdot}10^{-22}$, $|\lambda_5^4| = 7.9484{\cdot}10^{-21}$.

– y_5 population:

$$\underline{\gamma}_1^5 = (3.6241 \cdot 10^{-6},\ -6.2608 \cdot 10^{-1},\ \boldsymbol{7.7976 \cdot 10^{-1}},\ 3.7286 \cdot 10^{-14},\ -9.1053 \cdot 10^{-13})^t$$

$$\underline{\gamma}_2^5 = (5.2809 \cdot 10^{-6},\ \boldsymbol{-7.7976 \cdot 10^{-1}},\ -6.2608 \cdot 10^{-1},\ 5.0193 \cdot 10^{-14},\ -5.2359 \cdot 10^{-12})^t$$

$$\underline{\gamma}_3^5 = (\boldsymbol{\approx -1},\ -6.3868 \cdot 10^{-6},\ -4.8033 \cdot 10^{-7},\ -4.271 \cdot 10^{-8},\ 2.02 \cdot 10^{-4})^t$$

$$\underline{\gamma}_4^5 = (2.02 \cdot 10^{-4},\ 1.2855 \cdot 10^{-9},\ 9.4458 \cdot 10^{-11},\ -2.1215 \cdot 10^{-4},\ \boldsymbol{\approx 1})^t$$

$$\underline{\gamma}_5^5 = (1.4484 \cdot 10^{-10},\ 6.242 \cdot 10^{-14},\ 1.8755 \cdot 10^{-15},\ \boldsymbol{\approx 1},\ 2.1215 \cdot 10^{-4})^t$$

$|\lambda_1^5| = 6.0608{\cdot}10^{-17}$, $|\lambda_2^5| = 3.079{\cdot}10^{-16}$, $|\lambda_3^5| = 2.5517{\cdot}10^{-5}$, $|\lambda_4^5| = 7.68{\cdot}10^{3}$, $|\lambda_5^5| = 3.3663{\cdot}10^{18}$.

– y_6 population:

$$\underline{\gamma}_1^6 = (-7.6852 \cdot 10^{-8},\ 6.5199 \cdot 10^{-2},\ \boldsymbol{-9.9787 \cdot 10^{-1}},\ -6.5551 \cdot 10^{-10},\ 3.0221 \cdot 10^{-6})^t$$

$$\underline{\gamma}_2^6 = (-1.8852 \cdot 10^{-7},\ \boldsymbol{9.9787 \cdot 10^{-1}},\ 6.5199 \cdot 10^{-2},\ -1.7772 \cdot 10^{-10},\ 8.3742 \cdot 10^{-7})^t$$

$$\underline{\gamma}_3^6 = (1.9589 \cdot 10^{-2},\ 1.0363 \cdot 10^{-6},\ -2.9618 \cdot 10^{-6},\ 2.1302 \cdot 10^{-4},\ \boldsymbol{-9.9981 \cdot 10^{-1}})^t$$

$$\underline{\gamma}_4^6 = (\boldsymbol{9.9981 \cdot 10^{-1}},\ 1.7286 \cdot 10^{-7},\ -6.3812 \cdot 10^{-9},\ -4.1064 \cdot 10^{-6},\ 1.9589 \cdot 10^{-2})^t$$

$$\underline{\gamma}_5^6 = (-6.7154 \cdot 10^{-8},\ 4.1842 \cdot 10^{-14},\ -1.1631 \cdot 10^{-11},\ \boldsymbol{\approx 1},\ 2.1306 \cdot 10^{-4})^t$$

$|\lambda_1^6| = 9.0206{\cdot}10^{-26}$, $|\lambda_2^6| = 6.6226{\cdot}10^{-25}$, $|\lambda_3^6| = 7.0863{\cdot}10^{-15}$, $|\lambda_4^6| = 3.4637{\cdot}10^{-13}$, $|\lambda_5^6| = 8.9347{\cdot}10^{-1}$.

So that, the biological parameters, ordered from least identifiable to most identifiable are:

Q_4, Q_3, Q_1, β, α for y_1 and y_5,

Q_3, Q_4, Q_1, β, α for y_2,

Q_4, Q_1, Q_3, β, α for y_3,

Q_1, Q_3, Q_4, α, β for y_4,

Q_4, Q_3, β, Q_1, α for y_6.

- **Eigenvalues Method**

$$\begin{aligned}
\underline{\gamma}_1 &= (-1.0457 \cdot 10^{-8}, \ -5.2096 \cdot 10^{-2}, \ \mathbf{9.9864 \cdot 10^{-1}}, \ 2.42 \cdot 10^{-16}, \ 5.3782 \cdot 10^{-12})^t \\
\underline{\gamma}_2 &= (-1.3113 \cdot 10^{-7}, \ \mathbf{-9.9864 \cdot 10^{-1}}, \ -5.2096 \cdot 10^{-2}, \ 5.8381 \cdot 10^{-14}, \ 1.9392 \cdot 10^{-11})^t \\
\underline{\gamma}_3 &= (\mathbf{\approx -1}, \ 1.3150 \cdot 10^{-7}, \ -3.6111 \cdot 10^{-9}, \ 5.4246 \cdot 10^{-10}, \ -1.875 \cdot 10^{-6})^t \\
\underline{\gamma}_4 &= (-1.875 \cdot 10^{-6}, \ 1.9893 \cdot 10^{-11}, \ -4.3674 \cdot 10^{-12}, \ -2.12 \cdot 10^{-4}, \ \mathbf{\approx 1})^t \\
\underline{\gamma}_5 &= (1.4498 \cdot 10^{-10}, \ 6.2461 \cdot 10^{-14}, \ 1.8758 \cdot 10^{-15}, \ \mathbf{\approx 1}, \ 2.12 \cdot 10^{-4})^t
\end{aligned}$$

$$|\lambda_1| = 2.8778 \cdot 10^{-13}, \quad |\lambda_2| = 2.6266 \cdot 10^{-12}, \quad |\lambda_3| = 4.0221 \cdot 10^{-1}, \quad |\lambda_4| = 4.037 \cdot 10^{7}, \quad |\lambda_5| = 1.368 \cdot 10^{21}.$$

Consequently, the biological parameters, ordered from least identifiable to most identifiable are:

Q_4, Q_3, Q_1, β, α.

C.2 Therapies

C.2.1 5-populations model

Making the corresponding change of variables from χ_i to κ_i, we give the combinations of original therapy parameters κ_i in Table 14.

The conclusions obtained from the eigenvalues and eigenvectors are discussed in Subsection 6.1 and are as follows:

- For the initial tumor value $\approx 10\%$:
  - ***PCA* Method**
    - y_1 population:

$$\begin{aligned}
\underline{\gamma}_1^1 &= (\mathbf{-7.6699 \cdot 10^{-1}}, \ 6.4166 \cdot 10^{-1})^t \\
\underline{\gamma}_2^1 &= (6.4166 \cdot 10^{-1}, \ \mathbf{7.6699 \cdot 10^{-1}})^t
\end{aligned}$$

$$\left|\lambda_1^1\right| = 3.4272 \cdot 10^{-5}, \quad \left|\lambda_2^1\right| = 5.5529 \cdot 10^{-1}.$$

		κ_1	κ_2
$y_{0_5} \approx 0.1$	(χ_1, χ_2)	$1.9864 \cdot 10^{-1}$	$3.5395 \cdot 10^{-1}$
	$(0, \chi_2)$	$2.5535 \cdot 10^{-1}$	$3.0271 \cdot 10^{-1}$
	$(\tilde{\chi}_1, \chi_2)$	$2.9722 \cdot 10^{-1}$	$2.5961 \cdot 10^{-1}$
$y_{0_5} \approx 0.25$	(χ_1, χ_2)	$3.2139 \cdot 10^{-1}$	$2.395 \cdot 10^{-1}$
	$(0, \chi_2)$	$3.0702 \cdot 10^{-1}$	$2.5631 \cdot 10^{-1}$
	$(\tilde{\chi}_1, \chi_2)$	$2.6385 \cdot 10^{-1}$	$3.0285 \cdot 10^{-1}$
$y_{0_5} \approx 0.5$	(χ_1, χ_2)	$3.8444 \cdot 10^{-1}$	$1.6597 \cdot 10^{-1}$
	$(0, \chi_2)$	$3.4439 \cdot 10^{-1}$	$2.236 \cdot 10^{-1}$
	$(\tilde{\chi}_1, \chi_2)$	$2.4744 \cdot 10^{-1}$	$3.3602 \cdot 10^{-1}$
$y_{0_5} \approx 0.7$	(χ_1, χ_2)	$4.068 \cdot 10^{-1}$	$1.3797 \cdot 10^{-1}$
	$(0, \chi_2)$	$3.6143 \cdot 10^{-1}$	$2.0983 \cdot 10^{-1}$
	$(\tilde{\chi}_1, \chi_2)$	$2.4368 \cdot 10^{-1}$	$3.5119 \cdot 10^{-1}$

Table 14: Optimal values of the original therapy parameters κ_i for the 5-populations model, corresponding values χ_i computed in Table 6.

* y_2 population:

$$\underline{\gamma}_1^2 = (\mathbf{-7.6212 \cdot 10^{-1}}, \quad 6.4743 \cdot 10^{-1})^t$$

$$\underline{\gamma}_2^2 = (6.4743 \cdot 10^{-1}, \quad \mathbf{7.6212 \cdot 10^{-1}})^t$$

$$|\lambda_1^2| = 2.7201 \cdot 10^{-5}, \quad |\lambda_2^2| = 3.0934 \cdot 10^{-2}.$$

* y_3 population:

$$\underline{\gamma}_1^3 = (\mathbf{-7.6699 \cdot 10^{-1}}, \quad 6.4167 \cdot 10^{-1})^t$$

$$\underline{\gamma}_2^3 = (6.4167 \cdot 10^{-1}, \quad \mathbf{7.6699 \cdot 10^{-1}})^t$$

$$|\lambda_1^3| = 2.2037 \cdot 10^{-27}, \quad |\lambda_2^3| = 3.5775 \cdot 10^{-23}.$$

* y_4 population:

$$\underline{\gamma}_1^4 = (\mathbf{-7.6699 \cdot 10^{-1}}, \quad 6.4166 \cdot 10^{-1})^t$$

$$\underline{\gamma}_2^4 = (6.4166 \cdot 10^{-1}, \quad \mathbf{7.6699 \cdot 10^{-1}})^t$$

$$|\lambda_1^4| = 3.7309 \cdot 10^{-45}, \quad |\lambda_2^4| = 6.0375 \cdot 10^{-41}.$$

* y_5 population:

$$\underline{\gamma}_1^5 = (\mathbf{-7.6862 \cdot 10^{-1}}, \quad 6.397 \cdot 10^{-1})^t$$

$$\underline{\gamma}_2^5 = (6.397 \cdot 10^{-1}, \quad \mathbf{7.6862 \cdot 10^{-1}})^t$$

$$|\lambda_1^5| = 1.0488 \cdot 10^{-6}, \quad |\lambda_2^5| = 3.2419 \cdot 10^{-1}.$$

– **Eigenvalues Method**

$$\underline{\gamma}_1 = (\ \mathbf{-7.6436 \cdot 10^{-1}}, \ 6.4479 \cdot 10^{-1} \ )^t$$

$$\underline{\gamma}_2 = (\ 6.4479 \cdot 10^{-1}, \ \mathbf{7.6436 \cdot 10^{-1}} \ )^t$$

$$|\lambda_1| = 3.0176 \cdot 10^{-3}, \quad |\lambda_2| = 3.5512 \cdot 10^{1}.$$

- For the initial tumor value $\approx 25\%$:
  - ***PCA* Method**
    - y_1 population:

$$\underline{\gamma}_1^1 = (\ 6.4381 \cdot 10^{-1}, \ \mathbf{-7.6519 \cdot 10^{-1}} \ )^t$$

$$\underline{\gamma}_2^1 = (\ \mathbf{-7.6519 \cdot 10^{-1}}, \ -6.4381 \cdot 10^{-1} \ )^t$$

$$\left|\lambda_1^1\right| = 1.4496 \cdot 10^{-4}, \quad \left|\lambda_2^1\right| = 9.8528 \cdot 10^{-1}.$$

    - y_2 population:

$$\underline{\gamma}_1^2 = (\ 6.3093 \cdot 10^{-1}, \ \mathbf{-7.7584 \cdot 10^{-1}} \ )^t$$

$$\underline{\gamma}_2^2 = (\ \mathbf{-7.7584 \cdot 10^{-1}}, \ -6.3093 \cdot 10^{-1} \ )^t$$

$$\left|\lambda_1^2\right| = 8.6908 \cdot 10^{-5}, \quad \left|\lambda_2^2\right| = 1.0305 \cdot 10^{-2}.$$

    - y_3 population:

$$\underline{\gamma}_1^3 = (\ 6.4381 \cdot 10^{-1}, \ \mathbf{-7.6519 \cdot 10^{-1}} \ )^t$$

$$\underline{\gamma}_2^3 = (\ \mathbf{-7.6519 \cdot 10^{-1}}, \ -6.4381 \cdot 10^{-1} \ )^t$$

$$\left|\lambda_1^3\right| = 9.3396 \cdot 10^{-27}, \quad \left|\lambda_2^3\right| = 6.3478 \cdot 10^{-23}.$$

    - y_4 population:

$$\underline{\gamma}_1^4 = (\ 6.4381 \cdot 10^{-1}, \ \mathbf{-7.6519 \cdot 10^{-1}} \ )^t$$

$$\underline{\gamma}_2^4 = (\ \mathbf{-7.6519 \cdot 10^{-1}}, \ -6.4381 \cdot 10^{-1} \ )^t$$

$$\left|\lambda_1^4\right| = 1.5761 \cdot 10^{-44}, \quad \left|\lambda_2^4\right| = 1.0713 \cdot 10^{-40}.$$

    - y_5 population:

$$\underline{\gamma}_1^5 = (\ 6.4576 \cdot 10^{-1}, \ \mathbf{-7.6354 \cdot 10^{-1}} \ )^t$$

$$\underline{\gamma}_2^5 = (\ \mathbf{-7.6354 \cdot 10^{-1}}, \ -6.4576 \cdot 10^{-1} \ )^t$$

$$\left|\lambda_1^5\right| = 1.5703 \cdot 10^{-5}, \quad \left|\lambda_2^5\right| = 7.9453 \cdot 10^{-1}.$$

– **Eigenvalues Method**

$$\underline{\gamma}_1 = (\quad 6.4086 \cdot 10^{-1}, \quad \mathbf{-7.6765 \cdot 10^{-1}} \quad)^t$$

$$\underline{\gamma}_2 = (\quad \mathbf{-7.6765 \cdot 10^{-1}}, \quad -6.4086 \cdot 10^{-1} \quad)^t$$

$$|\lambda_1| = 1.0157 \cdot 10^{-2}, \quad |\lambda_2| = 7.3777 \cdot 10^{1}.$$

- For the initial tumor value ≈ 50%:

– ***PCA*** **Method**

∗ y_1 population:

$$\underline{\gamma}_1^1 = (\quad 5.4752 \cdot 10^{-1}, \quad \mathbf{-8.3679 \cdot 10^{-1}} \quad)^t$$

$$\underline{\gamma}_2^1 = (\quad \mathbf{-8.3679 \cdot 10^{-1}}, \quad -5.4752 \cdot 10^{-1} \quad)^t$$

$$\left|\lambda_1^1\right| = 8.9205 \cdot 10^{-4}, \quad \left|\lambda_2^1\right| = 2.4307 \cdot 10^{0}.$$

∗ y_2 population:

$$\underline{\gamma}_1^2 = (\quad \mathbf{-7.9379 \cdot 10^{-1}}, \quad 6.0819 \cdot 10^{-1} \quad)^t$$

$$\underline{\gamma}_2^2 = (\quad 6.0819 \cdot 10^{-1}, \quad \mathbf{7.9379 \cdot 10^{-1}} \quad)^t$$

$$\left|\lambda_1^2\right| = 3.2361 \cdot 10^{-4} \quad \left|\lambda_2^2\right| = 8.2872 \cdot 10^{-4}.$$

∗ y_3 population:

$$\underline{\gamma}_1^3 = (\quad 5.4752 \cdot 10^{-1}, \quad \mathbf{-8.3679 \cdot 10^{-1}} \quad)^t$$

$$\underline{\gamma}_2^3 = (\quad \mathbf{-8.3679 \cdot 10^{-1}}, \quad -5.4752 \cdot 10^{-1} \quad)^t$$

$$\left|\lambda_1^3\right| = 5.7472 \cdot 10^{-26}, \quad \left|\lambda_2^3\right| = 1.566 \cdot 10^{-22}.$$

∗ y_4 population:

$$\underline{\gamma}_1^4 = (\quad 5.4752 \cdot 10^{-1}, \quad \mathbf{-8.3679 \cdot 10^{-1}} \quad)^t$$

$$\underline{\gamma}_2^4 = (\quad \mathbf{-8.3679 \cdot 10^{-1}}, \quad -5.4752 \cdot 10^{-1} \quad)^t$$

$$\left|\lambda_1^4\right| = 9.699 \cdot 10^{-44}, \quad \left|\lambda_2^4\right| = 2.6428 \cdot 10^{-40}.$$

∗ y_5 population:

$$\underline{\gamma}_1^5 = (\quad 5.4919 \cdot 10^{-1}, \quad \mathbf{-8.3570 \cdot 10^{-1}} \quad)^t$$

$$\underline{\gamma}_2^5 = (\quad \mathbf{-8.3570 \cdot 10^{-1}}, \quad -5.4919 \cdot 10^{-1} \quad)^t$$

$$\left|\lambda_1^5\right| = 2.2724 \cdot 10^{-4}, \quad \left|\lambda_2^5\right| = 2.3537 \cdot 10^{0}.$$

– **Eigenvalues Method**

$$\underline{\gamma}_1 = (\quad 5.4456 \cdot 10^{-1}, \quad \mathbf{-8.3872 \cdot 10^{-1}} \quad)^t$$

$$\underline{\gamma}_2 = (\quad \mathbf{-8.3872 \cdot 10^{-1}}, \quad -5.4456 \cdot 10^{-1} \quad)^t$$

$$|\lambda_1| = 4.6011 \cdot 10^{-2}, \quad |\lambda_2| = 1.8569 \cdot 10^{2}.$$

- For the initial tumor value $\approx 70\%$:

– ***PCA*** **Method**

* y_1 population:

$$\underline{\gamma}_1^1 = (\quad 5.0425 \cdot 10^{-1}, \quad \mathbf{-8.6356 \cdot 10^{-1}} \quad)^t$$

$$\underline{\gamma}_2^1 = (\quad \mathbf{-8.6356 \cdot 10^{-1}}, \quad -5.0425 \cdot 10^{-1} \quad)^t$$

$$\left|\lambda_1^1\right| = 1.7339 \cdot 10^{-3}, \quad \left|\lambda_2^1\right| = 2.9859 \cdot 10^{0}.$$

* y_2 population:

$$\underline{\gamma}_1^2 = (\quad \mathbf{-8.9758 \cdot 10^{-1}}, \quad 4.4085 \cdot 10^{-1} \quad)^t$$

$$\underline{\gamma}_2^2 = (\quad 4.4085 \cdot 10^{-1}, \quad \mathbf{8.9758 \cdot 10^{-1}} \quad)^t$$

$$\left|\lambda_1^2\right| = 3.529 \cdot 10^{-4}, \quad \left|\lambda_2^2\right| = 1.353 \cdot 10^{-3}.$$

* y_3 population:

$$\underline{\gamma}_1^3 = (\quad 5.0425 \cdot 10^{-1}, \quad \mathbf{-8.6356 \cdot 10^{-1}} \quad)^t$$

$$\underline{\gamma}_2^3 = (\quad \mathbf{-8.6356 \cdot 10^{-1}}, \quad -5.0425 \cdot 10^{-1} \quad)^t$$

$$\left|\lambda_1^3\right| = 1.1171 \cdot 10^{-25}, \quad \left|\lambda_2^3\right| = 1.9237 \cdot 10^{-22}.$$

* y_4 population:

$$\underline{\gamma}_1^4 = (\quad 5.0425 \cdot 10^{-1}, \quad \mathbf{-8.6356 \cdot 10^{-1}} \quad)^t$$

$$\underline{\gamma}_2^4 = (\quad \mathbf{-8.6356 \cdot 10^{-1}}, \quad -5.0425 \cdot 10^{-1} \quad)^t$$

$$\left|\lambda_1^4\right| = 1.8852 \cdot 10^{-43}, \quad \left|\lambda_2^4\right| = 3.2465 \cdot 10^{-40}.$$

* y_5 population:

$$\underline{\gamma}_1^5 = (\quad 5.0544 \cdot 10^{-1}, \quad \mathbf{-8.6286 \cdot 10^{-1}} \quad)^t$$

$$\underline{\gamma}_2^5 = (\quad \mathbf{-8.6286 \cdot 10^{-1}}, \quad -5.0544 \cdot 10^{-1} \quad)^t$$

$$\left|\lambda_1^5\right| = 5.7514 \cdot 10^{-4}, \quad \left|\lambda_2^5\right| = 3.0856 \cdot 10^{0}.$$

– **Eigenvalues Method**

$$\underline{\gamma}_1 = (\quad 5.0207 \cdot 10^{-1}, \quad \mathbf{-8.6483 \cdot 10^{-1}} \quad)^t$$

$$\underline{\gamma}_2 = (\quad \mathbf{-8.6483 \cdot 10^{-1}}, \quad -5.0207 \cdot 10^{-1} \quad)^t$$

$$|\lambda_1| = 7.9754 \cdot 10^{-2}, \quad |\lambda_2| = 2.2152 \cdot 10^{2}.$$

C.2.2 6-populations model

Making the corresponding change of variables from χ_i to κ_i, we give the combinations of original therapy parameters κ_i in Table 15.

		κ_1	κ_2	κ_3
$y_{0_5} \approx 0.1$	(χ_1, χ_2, χ_3)	$2.2519 \cdot 10^{-1}$	$3.3307 \cdot 10^{-1}$	$9.26 \cdot 10^{-1}$
	$(0, \chi_2, \chi_3)$	$2.9859 \cdot 10^{-1}$	$2.6064 \cdot 10^{-1}$	$9.231 \cdot 10^{-1}$
	$(\tilde{\chi}_1, \chi_2, \chi_3)$	$2.7645 \cdot 10^{-1}$	$2.8373 \cdot 10^{-1}$	$7.8266 \cdot 10^{-2}$
	$(0, 0, \chi_3)$	–	–	–
	$(0, \tilde{\chi}_2, \chi_3)$	$2.8373 \cdot 10^{-1}$	$2.762 \cdot 10^{-1}$	$5.1356 \cdot 10^{-1}$
	$(\tilde{\chi}_1, 0, \chi_3)$	–	–	–
	$(\tilde{\chi}_1, \tilde{\chi}_2, \chi_3)$	$2.9222 \cdot 10^{-1}$	$2.6722 \cdot 10^{-1}$	$5.1296 \cdot 10^{-1}$
$y_{0_5} \approx 0.25$	(χ_1, χ_2, χ_3)	$3.5495 \cdot 10^{-1}$	$2.0242 \cdot 10^{-1}$	$8.1356 \cdot 10^{-1}$
	$(0, \chi_2, \chi_3)$	$3.6446 \cdot 10^{-1}$	$1.8958 \cdot 10^{-1}$	$8.1251 \cdot 10^{-1}$
	$(\tilde{\chi}_1, \chi_2, \chi_3)$	$2.7463 \cdot 10^{-1}$	$2.9729 \cdot 10^{-1}$	$8.2442 \cdot 10^{-1}$
	$(0, 0, \chi_3)$	–	–	–
	$(0, \tilde{\chi}_2, \chi_3)$	$3.4899 \cdot 10^{-1}$	$2.099 \cdot 10^{-1}$	$5.0363 \cdot 10^{-1}$
	$(\tilde{\chi}_1, 0, \chi_3)$	–	–	–
	$(\tilde{\chi}_1, \tilde{\chi}_2, \chi_3)$	$2.5641 \cdot 10^{-1}$	$3.1552 \cdot 10^{-1}$	$5.1576 \cdot 10^{-1}$
$y_{0_5} \approx 0.5$	(χ_1, χ_2, χ_3)	$3.8834 \cdot 10^{-1}$	$1.632 \cdot 10^{-1}$	$7.1235 \cdot 10^{-1}$
	$(0, \chi_2, \chi_3)$	$3.8701 \cdot 10^{-1}$	$1.6525 \cdot 10^{-1}$	$7.126 \cdot 10^{-1}$
	$(\tilde{\chi}_1, \chi_2, \chi_3)$	$2.6561 \cdot 10^{-1}$	$3.1972 \cdot 10^{-1}$	$7.4101 \cdot 10^{-1}$
	$(0, 0, \chi_3)$	–	–	–
	$(0, \tilde{\chi}_2, \chi_3)$	$3.7464 \cdot 10^{-1}$	$1.8278 \cdot 10^{-1}$	$4.9851 \cdot 10^{-1}$
	$(\tilde{\chi}_1, 0, \chi_3)$	–	–	–
	$(\tilde{\chi}_1, \tilde{\chi}_2, \chi_3)$	$2.4899 \cdot 10^{-1}$	$3.3537 \cdot 10^{-1}$	$5.1967 \cdot 10^{-1}$
$y_{0_5} \approx 0.7$	(χ_1, χ_2, χ_3)	$4.5894 \cdot 10^{-1}$	$3.775 \cdot 10^{-2}$	$6.3477 \cdot 10^{-1}$
	$(0, \chi_2, \chi_3)$	$3.9206 \cdot 10^{-1}$	$1.6323 \cdot 10^{-1}$	$6.4344 \cdot 10^{-1}$
	$(\tilde{\chi}_1, \chi_2, \chi_3)$	$2.31 \cdot 10^{-1}$	$3.6649 \cdot 10^{-1}$	$7.1363 \cdot 10^{-1}$
	$(0, 0, \chi_3)$	–	–	–
	$(0, \tilde{\chi}_2, \chi_3)$	$3.9041 \cdot 10^{-1}$	$1.6678 \cdot 10^{-1}$	$5.1187 \cdot 10^{-1}$
	$(\tilde{\chi}_1, 0, \chi_3)$	–	–	–
	$(\tilde{\chi}_1, \tilde{\chi}_2, \chi_3)$	$2.2471 \cdot 10^{-1}$	$3.6974 \cdot 10^{-1}$	$5.2455 \cdot 10^{-1}$

Table 15: Optimal values of the original therapy parameters for the 6-populations model.

The conclusions obtained from the eigenvalues and eigenvectors are studied at Subsection 6.2 and are the following ones:

- For the initial tumor value $\approx 10\%$:
  - ***PCA* Method**
    - y_1 population:

$$\underline{\gamma}_1^1 = (\mathbf{7.4497 \cdot 10^{-1}}, \ -6.6421 \cdot 10^{-1}, \ -6.1959 \cdot 10^{-2})^t$$

$$\underline{\gamma}_2^1 = (9.6691 \cdot 10^{-2}, \ 1.5614 \cdot 10^{-2}, \ \mathbf{9.9519 \cdot 10^{-1}})^t$$

$$\underline{\gamma}_3^1 = (-6.6005 \cdot 10^{-1}, \ \mathbf{-7.4738 \cdot 10^{-1}}, \ 7.5855 \cdot 10^{-2})^t$$

$$\left|\lambda_1^1\right| = 1.6403 \cdot 10^{-5}, \quad \left|\lambda_2^1\right| = 3.437 \cdot 10^{-3}, \quad \left|\lambda_3^1\right| = 9.253 \cdot 10^{-1}.$$

    - y_2 population:

$$\underline{\gamma}_1^2 = (\mathbf{7.3757 \cdot 10^{-1}}, \ -6.7511 \cdot 10^{-1}, \ 1.4357 \cdot 10^{-2})^t$$

$$\underline{\gamma}_2^2 = (4.1287 \cdot 10^{-2}, \ 6.6308 \cdot 10^{-2}, \ \mathbf{9.9694 \cdot 10^{-1}})^t$$

$$\underline{\gamma}_3^2 = (-6.74 \cdot 10^{-1}, \ \mathbf{-7.3473 \cdot 10^{-1}}, \ 7.678 \cdot 10^{-2})^t$$

$$\left|\lambda_1^2\right| = 1.4696 \cdot 10^{-5}, \quad \left|\lambda_2^2\right| = 2.6888 \cdot 10^{-4}, \quad \left|\lambda_3^2\right| = 2.501 \cdot 10^{-2}.$$

    - y_3 population:

$$\underline{\gamma}_1^3 = (\mathbf{7.4497 \cdot 10^{-1}}, \ -6.6421 \cdot 10^{-1}, \ -6.1959 \cdot 10^{-2})^t$$

$$\underline{\gamma}_2^3 = (9.6691 \cdot 10^{-2}, \ 1.5614 \cdot 10^{-2}, \ \mathbf{9.9519 \cdot 10^{-1}})^t$$

$$\underline{\gamma}_3^3 = (-6.6005 \cdot 10^{-1}, \ \mathbf{-7.4738 \cdot 10^{-1}}, \ 7.5855 \cdot 10^{-2})^t$$

$$\left|\lambda_1^3\right| = 4.3597 \cdot 10^{-27}, \quad \left|\lambda_2^3\right| = 9.1356 \cdot 10^{-25}, \quad \left|\lambda_3^3\right| = 2.4594 \cdot 10^{-22}.$$

    - y_4 population:

$$\underline{\gamma}_1^4 = (\mathbf{7.4497 \cdot 10^{-1}}, \ -6.6421 \cdot 10^{-1}, \ -6.1959 \cdot 10^{-2})^t$$

$$\underline{\gamma}_2^4 = (9.6691 \cdot 10^{-2}, \ 1.5614 \cdot 10^{-2}, \ \mathbf{9.9519 \cdot 10^{-1}})^t$$

$$\underline{\gamma}_3^4 = (-6.6005 \cdot 10^{-1}, \ \mathbf{-7.4738 \cdot 10^{-1}}, \ 7.5855 \cdot 10^{-2})^t$$

$$\left|\lambda_1^4\right| = 2.4168 \cdot 10^{-43}, \quad \left|\lambda_2^4\right| = 5.0642 \cdot 10^{-41}, \quad \left|\lambda_3^4\right| = 1.3634 \cdot 10^{-38}.$$

    - y_5 population:

$$\underline{\gamma}_1^5 = (\mathbf{7.4558 \cdot 10^{-1}}, \ -6.5976 \cdot 10^{-1}, \ 9.3935 \cdot 10^{-2})^t$$

$$\underline{\gamma}_2^5 = (7.221 \cdot 10^{-2}, \ -6.0144 \cdot 10^{-2}, \ \mathbf{-9.9557 \cdot 10^{-1}})^t$$

$$\underline{\gamma}_3^5 = (6.6249 \cdot 10^{-1}, \ \mathbf{7.4906 \cdot 10^{-1}}, \ 2.7992 \cdot 10^{-3})^t$$

$\left|\lambda_1^5\right| = 3.6679 \cdot 10^{-7}, \quad \left|\lambda_2^5\right| = 4.5494 \cdot 10^{-5}, \quad \left|\lambda_3^5\right| = 2.3514 \cdot 10^{-1}.$

  * y_6 population:

$$\underline{\gamma}_1^6 = (\mathbf{7.5086 \cdot 10^{-1}}, \quad -6.6029 \cdot 10^{-1}, \quad 1.5129 \cdot 10^{-2})^t$$

$$\underline{\gamma}_2^6 = (9.2076 \cdot 10^{-2}, \quad 1.2733 \cdot 10^{-1}, \quad \mathbf{9.8758 \cdot 10^{-1}})^t$$

$$\underline{\gamma}_3^6 = (-6.5402 \cdot 10^{-1}, \quad \mathbf{-7.4014 \cdot 10^{-1}}, \quad 1.5641 \cdot 10^{-1})^t$$

$\left|\lambda_1^6\right| = 4.3436 \cdot 10^{-12}, \quad \left|\lambda_2^6\right| = 1.8453 \cdot 10^{-6}, \quad \left|\lambda_3^6\right| = 5.9472 \cdot 10^{-1}.$

- **Eigenvalues Method**

$$\underline{\gamma}_1 = (\mathbf{7.4356 \cdot 10^{-1}}, \quad -6.6663 \cdot 10^{-1}, \quad -5.2285 \cdot 10^{-2})^t$$

$$\underline{\gamma}_2 = (6.6641 \cdot 10^{-2}, \quad -3.925 \cdot 10^{-3}, \quad \mathbf{9.9777 \cdot 10^{-1}})^t$$

$$\underline{\gamma}_3 = (-6.6534 \cdot 10^{-1}, \quad \mathbf{-7.4538 \cdot 10^{-1}}, \quad 4.1506 \cdot 10^{-2})^t$$

$|\lambda_1| = 2.3963 \cdot 10^{-3}, \quad |\lambda_2| = 1.1732 \cdot 10^{-1}, \quad |\lambda_3| = 3.9026 \cdot 10^{1}.$

- For the initial tumor value $\approx 25\%$:

  - ***PCA* Method**

    * y_1 population:

$$\underline{\gamma}_1^1 = (5.9245 \cdot 10^{-1}, \quad \mathbf{-7.9011 \cdot 10^{-1}}, \quad -1.5727 \cdot 10^{-1})^t$$

$$\underline{\gamma}_2^1 = (1.4472 \cdot 10^{-1}, \quad -8.7662 \cdot 10^{-2}, \quad \mathbf{9.8558 \cdot 10^{-1}})^t$$

$$\underline{\gamma}_3^1 = (\mathbf{-7.925 \cdot 10^{-1}}, \quad -6.0667 \cdot 10^{-1}, \quad 6.2412 \cdot 10^{-2})^t$$

$\left|\lambda_1^1\right| = 8.0126 \cdot 10^{-5}, \quad \left|\lambda_2^1\right| = 4.0697 \cdot 10^{-3}, \quad \left|\lambda_3^1\right| = 4.9202 \cdot 10^{0}.$

    * y_2 population:

$$\underline{\gamma}_1^2 = (-7.0121 \cdot 10^{-2}, \quad 2.452 \cdot 10^{-1}, \quad \mathbf{9.6693 \cdot 10^{-1}})^t$$

$$\underline{\gamma}_2^2 = (5.9487 \cdot 10^{-1}, \quad \mathbf{-7.6782 \cdot 10^{-1}}, \quad 2.3785 \cdot 10^{-1})^t$$

$$\underline{\gamma}_3^2 = (\mathbf{-8.0076 \cdot 10^{-1}}, \quad -5.9188 \cdot 10^{-1}, \quad 9.2023 \cdot 10^{-2})^t$$

$\left|\lambda_1^2\right| = 2.4856 \cdot 10^{-5}, \quad \left|\lambda_2^2\right| = 8.4818 \cdot 10^{-5}, \quad \left|\lambda_3^2\right| = 1.5232 \cdot 10^{-1}.$

* y_3 population:

$$\underline{\gamma}_1^3 = (\quad 5.9245 \cdot 10^{-1}, \quad \mathbf{-7.9011 \cdot 10^{-1}}, \quad -1.5727 \cdot 10^{-1} \quad)^t$$

$$\underline{\gamma}_2^3 = (\quad 1.4472 \cdot 10^{-1}, \quad -8.7662 \cdot 10^{-2}, \quad \mathbf{9.8558 \cdot 10^{-1}} \quad)^t$$

$$\underline{\gamma}_3^3 = (\quad \mathbf{-7.925 \cdot 10^{-1}}, \quad -6.0667 \cdot 10^{-1}, \quad 6.2412 \cdot 10^{-2} \quad)^t$$

$$\left|\lambda_1^3\right| = 2.1298 \cdot 10^{-26}, \quad \left|\lambda_2^3\right| = 1.0817 \cdot 10^{-24}, \quad \left|\lambda_3^3\right| = 1.3078 \cdot 10^{-21}.$$

* y_4 population:

$$\underline{\gamma}_1^4 = (\quad 5.9245 \cdot 10^{-1}, \quad \mathbf{-7.9011 \cdot 10^{-1}}, \quad -1.5727 \cdot 10^{-1} \quad)^t$$

$$\underline{\gamma}_2^4 = (\quad 1.4472 \cdot 10^{-1}, \quad -8.7662 \cdot 10^{-2}, \quad \mathbf{9.8558 \cdot 10^{-1}} \quad)^t$$

$$\underline{\gamma}_3^4 = (\quad \mathbf{-7.925 \cdot 10^{-1}}, \quad -6.0667 \cdot 10^{-1}, \quad 6.2412 \cdot 10^{-2} \quad)^t$$

$$\left|\lambda_1^4\right| = 1.1806 \cdot 10^{-42}, \quad \left|\lambda_2^4\right| = 5.9964 \cdot 10^{-41}, \quad \left|\lambda_3^4\right| = 7.2495 \cdot 10^{-38}.$$

* y_5 population:

$$\underline{\gamma}_1^5 = (\quad 6.0081 \cdot 10^{-1}, \quad \mathbf{-7.9244 \cdot 10^{-1}}, \quad 1.0518 \cdot 10^{-1} \quad)^t$$

$$\underline{\gamma}_2^5 = (\quad -6.1589 \cdot 10^{-2}, \quad 8.5295 \cdot 10^{-2}, \quad \mathbf{9.9445 \cdot 10^{-1}} \quad)^t$$

$$\underline{\gamma}_3^5 = (\quad \mathbf{7.9702 \cdot 10^{-1}}, \quad 6.0395 \cdot 10^{-1}, \quad -2.44 \cdot 10^{-3} \quad)^t$$

$$\left|\lambda_1^5\right| = 8.1166 \cdot 10^{-6}, \quad \left|\lambda_2^5\right| = 1.1941 \cdot 10^{-3}, \quad \left|\lambda_3^5\right| = 7.787 \cdot 10^{-1}.$$

* y_6 population:

$$\underline{\gamma}_1^6 = (\quad 6.1007 \cdot 10^{-1}, \quad \mathbf{-7.8305 \cdot 10^{-1}}, \quad 1.2105 \cdot 10^{-1} \quad)^t$$

$$\underline{\gamma}_2^6 = (\quad -3.4405 \cdot 10^{-3}, \quad 1.5015 \cdot 10^{-1}, \quad \mathbf{9.8866 \cdot 10^{-1}} \quad)^t$$

$$\underline{\gamma}_3^6 = (\quad \mathbf{-7.9234 \cdot 10^{-1}}, \quad -6.0356 \cdot 10^{-1}, \quad 8.891 \cdot 10^{-2} \quad)^t$$

$$\left|\lambda_1^6\right| = 5.8201 \cdot 10^{-8}, \quad \left|\lambda_2^6\right| = 2.1214 \cdot 10^{-5}, \quad \left|\lambda_3^6\right| = 6.2711 \cdot 10^{0}.$$

– **Eigenvalues Method**

$$\underline{\gamma}_1 = (\quad 5.9829 \cdot 10^{-1}, \quad \mathbf{-7.9703 \cdot 10^{-1}}, \quad -8.2396 \cdot 10^{-2} \quad)^t$$

$$\underline{\gamma}_2 = (\quad 9.0725 \cdot 10^{-2}, \quad -3.4787 \cdot 10^{-2}, \quad \mathbf{9.9527 \cdot 10^{-1}} \quad)^t$$

$$\underline{\gamma}_3 = (\quad \mathbf{-7.9613 \cdot 10^{-1}}, \quad -6.0293 \cdot 10^{-1}, \quad 5.1499 \cdot 10^{-2} \quad)^t$$

$$|\lambda_1| = 1.2521 \cdot 10^{-2}, \quad |\lambda_2| = 3.0559 \cdot 10^{-1}, \quad |\lambda_3| = 2.2073 \cdot 10^{2}.$$

- For the initial tumor value $\approx 50\%$:

– ***PCA* Method**

- y_1 population:

$$\underline{\gamma}_1^1 = (\quad 4.881 \cdot 10^{-1}, \quad \mathbf{-7.8119 \cdot 10^{-1}}, \quad -3.8923 \cdot 10^{-1} \quad)^t$$

$$\underline{\gamma}_2^1 = (\quad 2.5986 \cdot 10^{-1}, \quad -2.9567 \cdot 10^{-1}, \quad \mathbf{9.1927 \cdot 10^{-1}} \quad)^t$$

$$\underline{\gamma}_3^1 = (\quad \mathbf{-8.332 \cdot 10^{-1}}, \quad -5.4984 \cdot 10^{-1}, \quad 5.8683 \cdot 10^{-2} \quad)^t$$

$$\left|\lambda_1^1\right| = 5.3766 \cdot 10^{-4}, \quad \left|\lambda_2^1\right| = 3.3535 \cdot 10^{-3}, \quad \left|\lambda_3^1\right| = 1.5088 \cdot 10^{1}.$$

- y_2 population:

$$\underline{\gamma}_1^2 = (\quad 1.9891 \cdot 10^{-2}, \quad 1.1082 \cdot 10^{-1}, \quad \mathbf{9.9364 \cdot 10^{-1}} \quad)^t$$

$$\underline{\gamma}_2^2 = (\quad 5.4499 \cdot 10^{-1}, \quad \mathbf{-8.3441 \cdot 10^{-1}}, \quad 8.2154 \cdot 10^{-2} \quad)^t$$

$$\underline{\gamma}_3^2 = (\quad \mathbf{-8.3821 \cdot 10^{-1}}, \quad -5.3989 \cdot 10^{-1}, \quad 7.6995 \cdot 10^{-2} \quad)^t$$

$$\left|\lambda_1^2\right| = 3.3572 \cdot 10^{-5}, \quad \left|\lambda_2^2\right| = 3.1525 \cdot 10^{-4}, \quad \left|\lambda_3^2\right| = 8.5983 \cdot 10^{-1}.$$

- y_3 population:

$$\underline{\gamma}_1^3 = (\quad 4.881 \cdot 10^{-1}, \quad \mathbf{-7.8119 \cdot 10^{-1}}, \quad -3.8923 \cdot 10^{-1} \quad)^t$$

$$\underline{\gamma}_2^3 = (\quad 2.5986 \cdot 10^{-1}, \quad -2.9567 \cdot 10^{-1}, \quad \mathbf{9.1927 \cdot 10^{-1}} \quad)^t$$

$$\underline{\gamma}_3^3 = (\quad \mathbf{-8.332 \cdot 10^{-1}}, \quad -5.4984 \cdot 10^{-1}, \quad 5.8683 \cdot 10^{-2} \quad)^t$$

$$\left|\lambda_1^3\right| = 1.4291 \cdot 10^{-25}, \quad \left|\lambda_2^3\right| = 8.9136 \cdot 10^{-25}, \quad \left|\lambda_3^3\right| = 4.0104 \cdot 10^{-21}.$$

- y_4 population:

$$\underline{\gamma}_1^4 = (\quad 4.881 \cdot 10^{-1}, \quad \mathbf{-7.8119 \cdot 10^{-1}}, \quad -3.8924 \cdot 10^{-1} \quad)^t$$

$$\underline{\gamma}_2^4 = (\quad 2.5986 \cdot 10^{-1}, \quad -2.9567 \cdot 10^{-1}, \quad \mathbf{9.1927 \cdot 10^{-1}} \quad)^t$$

$$\underline{\gamma}_3^4 = (\quad \mathbf{-8.332 \cdot 10^{-1}}, \quad -5.4984 \cdot 10^{-1}, \quad 5.8683 \cdot 10^{-2} \quad)^t$$

$$\left|\lambda_1^4\right| = 7.922 \cdot 10^{-42}, \quad \left|\lambda_2^4\right| = 4.9411 \cdot 10^{-41}, \quad \left|\lambda_3^4\right| = 2.2231 \cdot 10^{-37}.$$

- y_5 population:

$$\underline{\gamma}_1^5 = (\quad 5.4182 \cdot 10^{-1}, \quad \mathbf{-8.3954 \cdot 10^{-1}}, \quad -3.9961 \cdot 10^{-2} \quad)^t$$

$$\underline{\gamma}_2^5 = (\quad 7.9395 \cdot 10^{-2}, \quad 3.7916 \cdot 10^{-3}, \quad \mathbf{9.9684 \cdot 10^{-1}} \quad)^t$$

$$\underline{\gamma}_3^5 = (\quad \mathbf{-8.3674 \cdot 10^{-1}}, \quad -5.4328 \cdot 10^{-1}, \quad 6.871 \cdot 10^{-2} \quad)^t$$

$$\left|\lambda_1^5\right| = 2.1986 \cdot 10^{-5}, \quad \left|\lambda_2^5\right| = 9.1635 \cdot 10^{-3}, \quad \left|\lambda_3^5\right| = 3.1967 \cdot 10^{0}.$$

* y_6 population:

$$\underline{\gamma}_1^6 = (5.5314 \cdot 10^{-1}, \mathbf{-8.2648 \cdot 10^{-1}}, 1.0467 \cdot 10^{-1})^t$$

$$\underline{\gamma}_2^6 = (5.3945 \cdot 10^{-3}, 1.292 \cdot 10^{-1}, \mathbf{9.916 \cdot 10^{-1}})^t$$

$$\underline{\gamma}_3^6 = (\mathbf{-8.3307 \cdot 10^{-1}}, -5.4793 \cdot 10^{-1}, 7.5922 \cdot 10^{-2})^t$$

$\left|\lambda_1^6\right| = 2.3142 \cdot 10^{-5}$, $\left|\lambda_2^6\right| = 1.3534 \cdot 10^{-3}$, $\left|\lambda_3^6\right| = 3.5693 \cdot 10^{1}$.

– **Eigenvalues Method**

$$\underline{\gamma}_1 = (5.4025 \cdot 10^{-1}, \mathbf{-8.3559 \cdot 10^{-1}}, -9.963 \cdot 10^{-2})^t$$

$$\underline{\gamma}_2 = (1.0915 \cdot 10^{-1}, -4.7813 \cdot 10^{-2}, \mathbf{9.9287 \cdot 10^{-1}})^t$$

$$\underline{\gamma}_3 = (\mathbf{-8.344 \cdot 10^{-1}}, -5.4727 \cdot 10^{-1}, 6.5374 \cdot 10^{-2})^t$$

$|\lambda_1| = 3.5662 \cdot 10^{-2}$, $|\lambda_2| = 5.9336 \cdot 10^{-1}$, $|\lambda_3| = 9.456 \cdot 10^{2}$.

- For the initial tumor value $\approx 70\%$:

– ***PCA* Method**

* y_1 population:

$$\underline{\gamma}_1^1 = (4.2878 \cdot 10^{-1}, \mathbf{-8.8038 \cdot 10^{-1}}, 2.0267 \cdot 10^{-1})^t$$

$$\underline{\gamma}_2^1 = (-1.9656 \cdot 10^{-2}, 2.1519 \cdot 10^{-1}, \mathbf{9.7637 \cdot 10^{-1}})^t$$

$$\underline{\gamma}_3^1 = (\mathbf{-9.032 \cdot 10^{-1}}, -4.2263 \cdot 10^{-1}, 7.4966 \cdot 10^{-2})^t$$

$\left|\lambda_1^1\right| = 1.5137 \cdot 10^{-3}$, $\left|\lambda_2^1\right| = 6.4814 \cdot 10^{-3}$, $\left|\lambda_3^1\right| = 7.482 \cdot 10^{0}$.

* y_2 population:

$$\underline{\gamma}_1^2 = (6.1463 \cdot 10^{-2}, 5.8931 \cdot 10^{-2}, \mathbf{9.9637 \cdot 10^{-1}})^t$$

$$\underline{\gamma}_2^2 = (4.3531 \cdot 10^{-1}, \mathbf{-8.9989 \cdot 10^{-1}}, 2.6372 \cdot 10^{-2})^t$$

$$\underline{\gamma}_3^2 = (\mathbf{-8.9818 \cdot 10^{-1}}, -4.3211 \cdot 10^{-1}, 8.0963 \cdot 10^{-2})^t$$

$\left|\lambda_1^2\right| = 1.5828 \cdot 10^{-4}$, $\left|\lambda_2^2\right| = 5.2048 \cdot 10^{-4}$, $\left|\lambda_3^2\right| = 2.7998 \cdot 10^{-1}$.

* y_3 population:

$$\underline{\gamma}_1^3 = (4.2878 \cdot 10^{-1}, \mathbf{-8.8038 \cdot 10^{-1}}, 2.0267 \cdot 10^{-1})^t$$

$$\underline{\gamma}_2^3 = (-1.9656 \cdot 10^{-2}, 2.1519 \cdot 10^{-1}, \mathbf{9.7637 \cdot 10^{-1}})^t$$

$$\underline{\gamma}_3^3 = (\mathbf{-9.032 \cdot 10^{-1}}, -4.2263 \cdot 10^{-1}, 7.4966 \cdot 10^{-2})^t$$

$\left|\lambda_1^3\right| = 4.0235 \cdot 10^{-25}$, $\left|\lambda_2^3\right| = 1.7227 \cdot 10^{-24}$, $\left|\lambda_3^3\right| = 1.9887 \cdot 10^{-21}$.

$*$ y_4 population:

$$\underline{\gamma}_1^4 = (\quad 4.2878 \cdot 10^{-1}, \quad \mathbf{-8.8038 \cdot 10^{-1}}, \quad 2.0267 \cdot 10^{-1} \quad)^t$$

$$\underline{\gamma}_2^4 = (\quad -1.9656 \cdot 10^{-2}, \quad 2.1519 \cdot 10^{-1}, \quad \mathbf{9.7637 \cdot 10^{-1}} \quad)^t$$

$$\underline{\gamma}_3^4 = (\quad \mathbf{-9.032 \cdot 10^{-1}}, \quad -4.2263 \cdot 10^{-1}, \quad 7.4966 \cdot 10^{-2} \quad)^t$$

$$\left|\lambda_1^4\right| = 2.2304 \cdot 10^{-41}, \quad \left|\lambda_2^4\right| = 9.5498 \cdot 10^{-41}, \quad \left|\lambda_3^4\right| = 1.1024 \cdot 10^{-37}.$$

$*$ y_5 population:

$$\underline{\gamma}_1^5 = (\quad 4.1117 \cdot 10^{-1}, \quad \mathbf{-9.04 \cdot 10^{-1}}, \quad -1.1714 \cdot 10^{-1} \quad)^t$$

$$\underline{\gamma}_2^5 = (\quad 1.3729 \cdot 10^{-1}, \quad -6.5622 \cdot 10^{-2}, \quad \mathbf{9.8835 \cdot 10^{-1}} \quad)^t$$

$$\underline{\gamma}_3^5 = (\quad \mathbf{-9.0116 \cdot 10^{-1}}, \quad -4.2246 \cdot 10^{-1}, \quad 9.7132 \cdot 10^{-2} \quad)^t$$

$$\left|\lambda_1^5\right| = 1.4612 \cdot 10^{-4}, \quad \left|\lambda_2^5\right| = 1.0907 \cdot 10^{-2}, \quad \left|\lambda_3^5\right| = 2.6253 \cdot 10^{0}.$$

$*$ y_6 population:

$$\underline{\gamma}_1^6 = (\quad 4.3298 \cdot 10^{-1}, \quad \mathbf{-9.0003 \cdot 10^{-1}}, \quad 4.9821 \cdot 10^{-2} \quad)^t$$

$$\underline{\gamma}_2^6 = (\quad 6.3545 \cdot 10^{-2}, \quad 8.561 \cdot 10^{-2}, \quad \mathbf{9.943 \cdot 10^{-1}} \quad)^t$$

$$\underline{\gamma}_3^6 = (\quad \mathbf{-8.9916 \cdot 10^{-1}}, \quad -4.2734 \cdot 10^{-1}, \quad 9.426 \cdot 10^{-2} \quad)^t$$

$$\left|\lambda_1^6\right| = 1.5698 \cdot 10^{-4}, \quad \left|\lambda_2^6\right| = 6.1269 \cdot 10^{-3}, \quad \left|\lambda_3^6\right| = 1.8047 \cdot 10^{1}.$$

– **Eigenvalues Method**

$$\underline{\gamma}_1 = (\quad 4.2536 \cdot 10^{-1}, \quad \mathbf{-9.0453 \cdot 10^{-1}}, \quad -2.9712 \cdot 10^{-2} \quad)^t$$

$$\underline{\gamma}_2 = (\quad 8.5359 \cdot 10^{-2}, \quad 7.4138 \cdot 10^{-3}, \quad \mathbf{9.9632 \cdot 10^{-1}} \quad)^t$$

$$\underline{\gamma}_3 = (\quad \mathbf{-9.0099 \cdot 10^{-1}}, \quad -4.2634 \cdot 10^{-1}, \quad 8.0364 \cdot 10^{-2} \quad)^t$$

$$|\lambda_1| = 5.2747 \cdot 10^{-2}, \quad |\lambda_2| = 7.7509 \cdot 10^{-1}, \quad |\lambda_3| = 3.9441 \cdot 10^{2}.$$

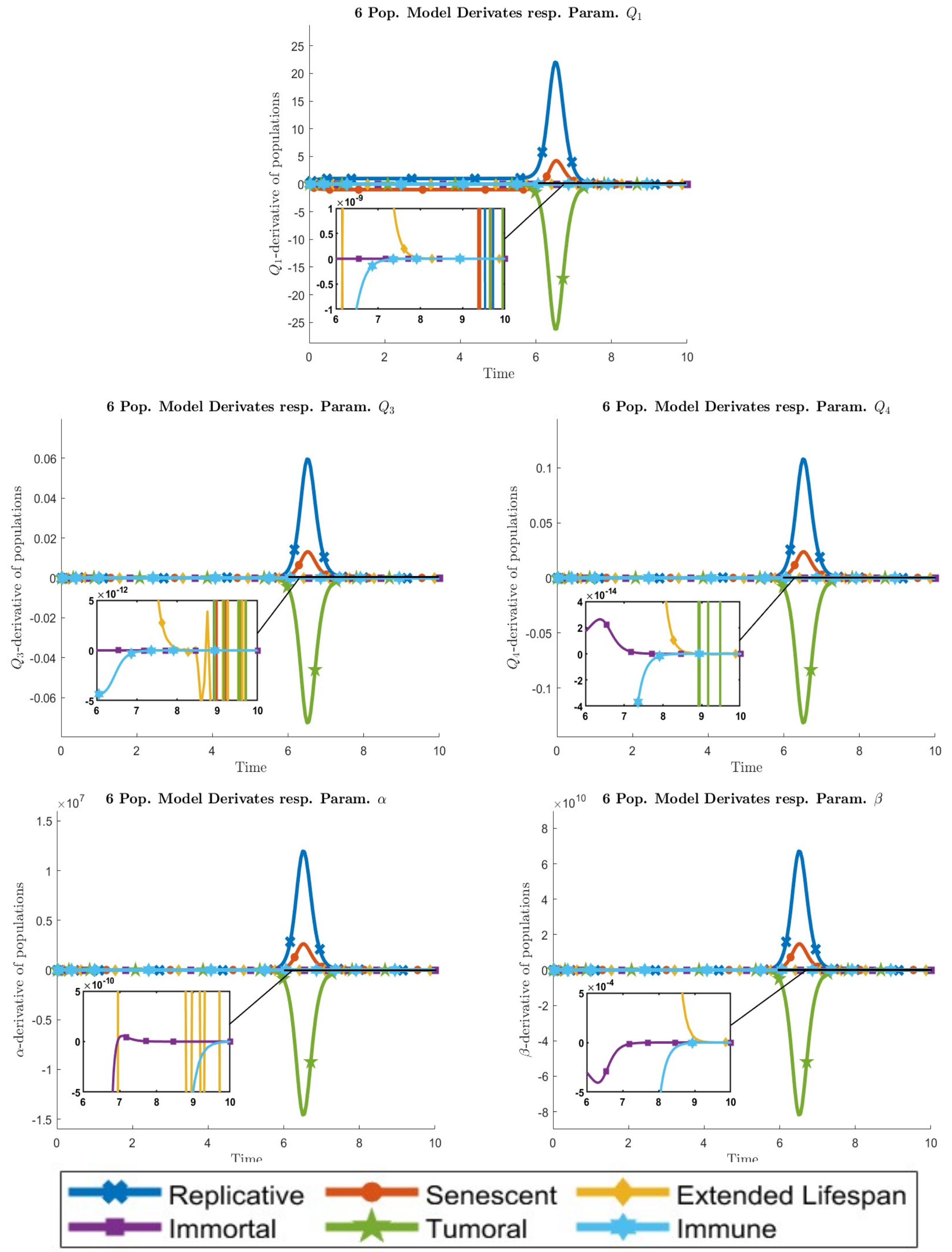

Figure 11: Dynamical behavior of the derivatives of the six cell types for each transmission parameter. The upper graphic is for Q_1, the middle-left one for Q_3, the middle-right one for Q_4, the bottom-left one for α and the bottom-right one for β. Line codes, y_1: blue crossed, y_2: red circled, y_3: yellow diamonded, y_4: purple squared, y_5: green 5 points stared, y_6: cyan 6 points stared.

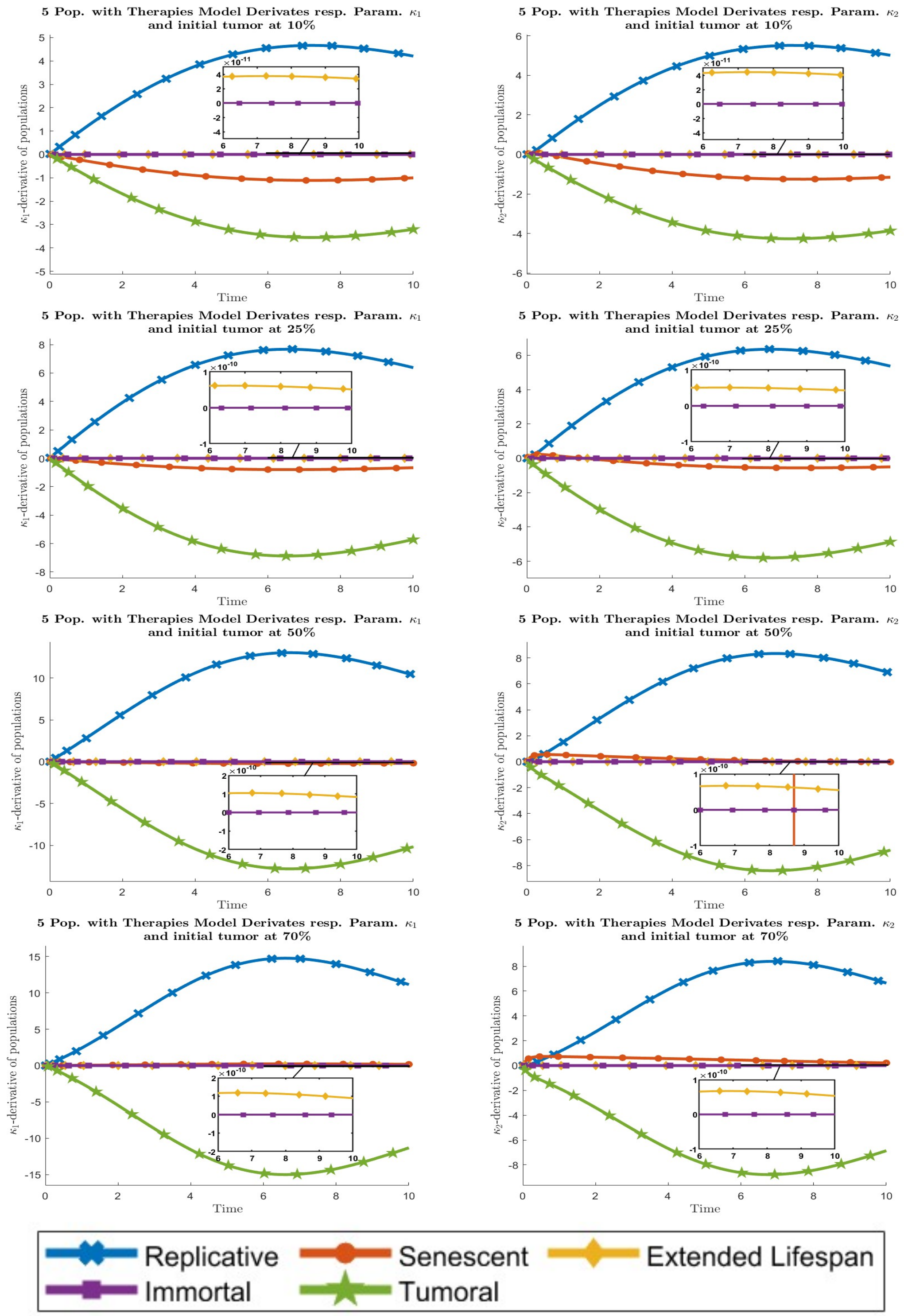

Figure 12: Dynamical behavior of the derivatives of the five cell types with therapies for the optimal values of the transmission parameters (Q_1, α, β). By columns, the left one is for κ_1 and the right one for κ_2. By rows, the graphics are for the initial tumor values $\approx$ 10%, 25%, 50% and 70% respectively from top to bottom. Line codes, y_1: blue crossed, y_2: red circled, y_3: yellow diamonded, y_4: purple squared, y_5: green 5 points stared.

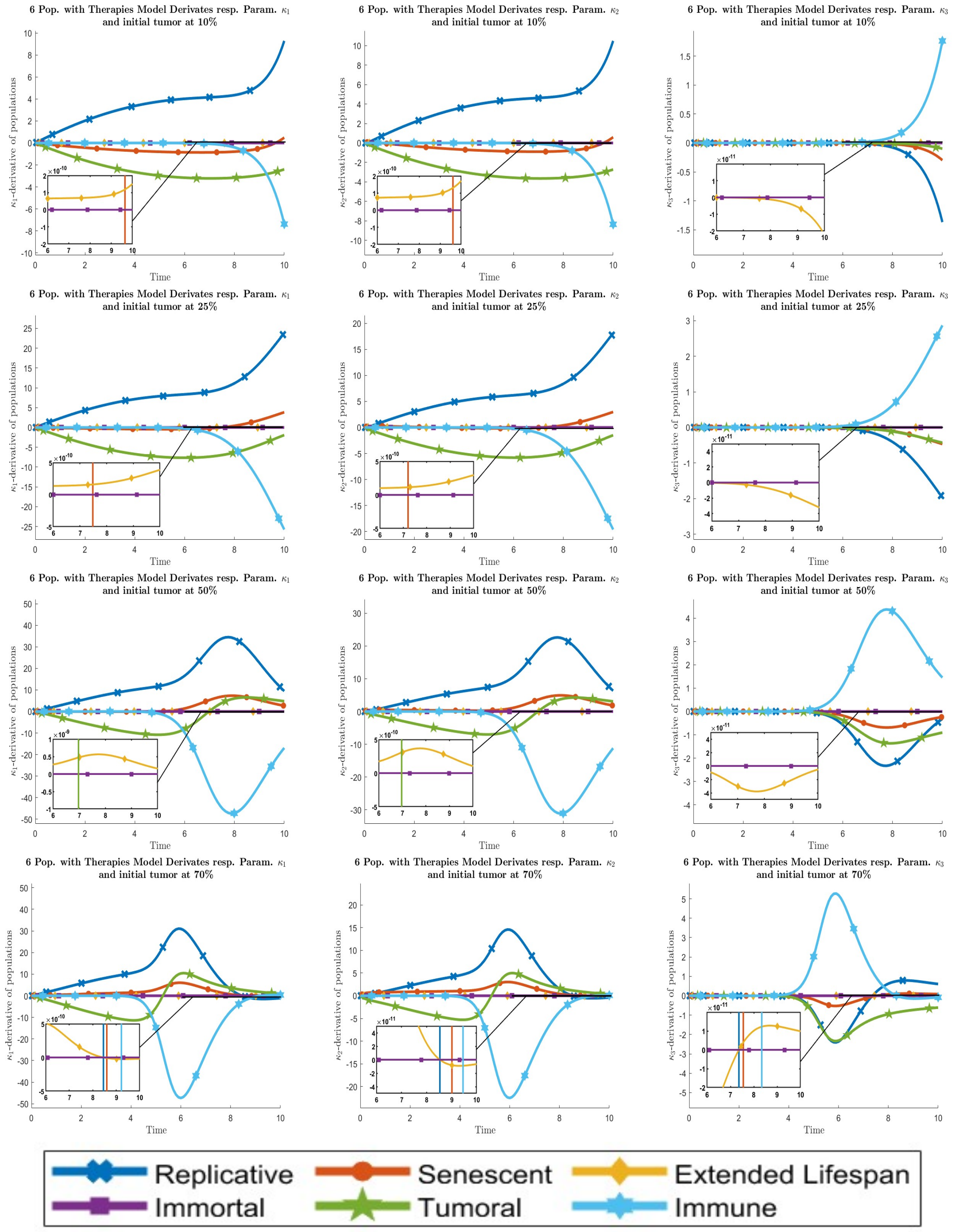

Figure 13: Dynamical behavior of the derivatives of the six cell types with therapies for the optimal values of the transmission parameters (Q_1, α, β). By columns, the left one for κ_1, the middle one for κ_2 and the right one for κ_3. By rows, the graphics are for the initial tumor values $\approx$ 10%, 25%, 50% and 70% respectively from top to bottom. Line codes, y_1: blue crossed, y_2: red circled, y_3: yellow diamonded, y_4: purple squared, y_5: green 5 points stared, y_6: cyan 6 points stared.
